# Clinical variables associated with immune checkpoint inhibitor outcomes in patients with metastatic urothelial carcinoma: a multicentre retrospective cohort study

**DOI:** 10.1136/bmjopen-2023-081480

**Published:** 2024-03-29

**Authors:** Soumaya Labidi, Nicholas Meti, Reeta Barua, Mengqi Li, Jamila Riromar, Di Maria Jiang, Nazanin Fallah-Rad, Srikala S Sridhar, Sonia V Del Rincon, Rossanna C Pezo, Cristiano Ferrario, Susanna Cheng, Adrian G Sacher, April A N Rose

**Affiliations:** 1 Segal Cancer Centre, Jewish General Hospital, Montreal, Québec, Canada; 2 Gerald Bronfman Department of Oncology, McGill University, Montreal, Québec, Canada; 3 St Mary Hospital, Montreal, Quebec, Canada; 4 Toronto East Health Network Michael Garron Hospital, Toronto, Ontario, Canada; 5 Lady Davis Institute for Medical Research, Montreal, Québec, Canada; 6 Division of Experimental Medicine, Faculty of Medicine, McGill University, Montreal, Quebec, Canada; 7 National Oncology Center, The Royal Hospital, Seeb, Muscat, Oman; 8 Medical Oncology, Princess Margaret Hospital Cancer Centre, Toronto, Ontario, Canada; 9 Odette Cancer Center, Sunnybrook Health Sciences Center, Toronto, Ontario, Canada

**Keywords:** urological tumours, adult oncology, clinical reasoning, epidemiology, oncology

## Abstract

**Objectives:**

Immune checkpoint inhibitors (ICIs) are indicated for metastatic urothelial cancer (mUC), but predictive and prognostic factors are lacking. We investigated clinical variables associated with ICI outcomes.

**Methods:**

We performed a multicentre retrospective cohort study of 135 patients who received ICI for mUC, 2016–2021, at three Canadian centres. Clinical characteristics, body mass index (BMI), metastatic sites, neutrophil-to-lymphocyte ratio (NLR), response and survival were abstracted from chart review.

**Results:**

We identified 135 patients and 62% had received ICI as a second-line or later treatment for mUC. A BMI ≥25 was significantly correlated to a higher overall response rate (ORR) (45.4% vs 16.3%, p value=0.020). Patients with BMI ≥30 experienced longer median overall survival (OS) of 24.8 vs 14.4 for 25≤BMI<30 and 8.5 months for BMI <25 (p value=0.012). The ORR was lower in the presence of bone metastases (16% vs 41%, p value=0.006) and liver metastases (16% vs 39%, p value=0.013). Metastatic lymph nodes were correlated with higher ORR (40% vs 20%, p value=0.032). The median OS for bone metastases was 7.3 versus 18 months (p value <0.001). Patients with liver metastases had a median OS of 8.6 versus 15 months (p value=0.006). No difference for lymph nodes metastases (13.5 vs 12.7 months, p value=0.175) was found. NLR ≥4 had worse OS (8.2 vs 17.7 months, p value=0.0001). In multivariate analysis, BMI ≥30, bone metastases, NLR ≥4, performance status ≥2 and line of ICI ≥2 were independent factors for OS.

**Conclusions:**

Our data identified BMI and bone metastases as novel clinical biomarkers that were independently associated with ICI outcomes in mUC. External and prospective validation are warranted.

**STRENGTHS AND LIMITATIONS OF THIS STUDY**

- This is a multicentre cohort study, with a large number of patients, treated with immune checkpoint inhibitors for metastatic urothelial cancer at three Canadian institutions.
- We identified reproductible clinical prognostic factors related with response and survival outcomes, including bone metastases for which there are limited data available from prospective studies.
- The retrospective study design may lead to selection bias related to population studied.

## Introduction

Bladder cancer represents the 10th most common cancer in the world, with approximately 550 000 new cases annually, and accounts for 2.1% of all cancer deaths according to GLOBOCAN 2020.^1^ There is a male predominance and smoking is the most common risk factor. In most cases, urothelial carcinomas arise from the urothelium of the bladder but can also develop from the upper urinary tract (renal pelvis and ureters). Tumours invading the detrusor muscle, that is, muscle invasive tumours, account with upfront metastatic disease in 25% of the cases.^2^ Despite multimodality management for non-metastatic muscle invasive bladder cancer, 50% of these patients will relapse, with distant metastases in most of the cases.^3 4^ Platinum-based chemotherapy remains the standard first-line treatment for metastatic disease.^3 4^ Patients who respond or have stable disease following chemotherapy are eligible for subsequent maintenance therapy with the anti-programmed death ligand 1 (PD-L1) immune checkpoint inhibitor (ICI), Avelumab,^5^ while those who fail to respond to chemotherapy subsequently can receive the anti-programmed death 1 (PD-1) ICI, Pembrolizumab.^6^ Other treatment options in the platinum-refractory setting include Enfortumab Vedotin and Erdafitinib in patients with susceptible Fibroblast Growth Factor Receptor alterations, and chemotherapy with taxanes (Paclitaxel and Docetaxel) or Vinflunine.^7–10^ In the KEYNOTE-045 randomised phase III trial, Pembrolizumab showed a significantly longer overall survival (OS) of 10.3 months versus 7.4 months in patients with platinum refractory metastatic urothelial cancer (mUC) compared with chemotherapy (Docetaxel, Paclitaxel or Vinflunine) (HR: 0.73, 95% CI: 0.59 to 0.91, p value=0.002), with a lower rate of any grade adverse events (60.9% vs 90.2%).^6^ Long-term results confirmed an OS benefit and a favourable safety profile.^11^ The PD-L1 checkpoint inhibitor Atezolizumab was also associated with durable objective responses in the IMvigpr210 phase II trial, with favourable safety profile,^12^ but failed to show survival benefit over chemotherapy in the IMvigor211 phase III trial.^13^ In phase I/II trials CheckMate275 and CheckMate032, Nivolumab, a PD-1 inhibitor, achieved objective responses alone or in combination with Ipilimumab, with a manageable adverse events profile.^14 15^ Despite responses seen with ICI in the platinum-refractory setting, not all patients will derive benefit and there is a lack of valid prognostic and predictive biomarkers.

In this study, we aimed to evaluate if clinical prognostic markers, that have been defined for other cancer types, are relevant to mUC. In fact, several retrospective studies have addressed the relationship between obesity and outcomes in patients receiving ICI. In melanoma, non-small cell lung cancer (NSCLC) and renal cell cancer (RCC), obesity appears to be linked to better progression-free survival (PFS) and OS.^16–18^ And a recent systematic review of 18 retrospective studies across different cancer types, by Indini *et al*, also found an association between high body mass index (BMI) and improved ICI outcomes; yet a strong positive correlation could not be concluded due to the heterogeneity of the studies.^19^ As such, the relationship between BMI and immunotherapy outcomes in urothelial cancer remains poorly understood.

The tumour immune microenvironment of different metastatic locations may also affect the response to ICI.^20 21^ Retrospective studies reported differences in organ-specific responses and organ-specific OS with immunotherapy in several types of cancer.^22–24^

Neutrophil-to-lymphocyte ratio (NLR) is an available marker of systemic response to inflammation, derived from the absolute counts of neutrophils and lymphocytes on a blood count.^25^ It is a well-established poor prognostic factor in several cancers, independently of treatment type.^25–27^

The aim of this retrospective analysis was to assess the association between high BMI, site of metastases, NLR and outcome in terms of response and OS in a population of patients with mUC treated with ICI.

### Patients and methods

#### Patient population, characteristics and outcome

We performed a multicentre retrospective cohort study analysis of patients with mUC, from three Canadian cancer centres: Segal Cancer Center, Jewish General Hospital (JGH), Princess Margaret Cancer Center, University Health Network (PM-UHN) and Odette Cancer Center, Sunnybrook Health Sciences Center (SHSC). Patients with histologically proven mUC, who received at least one dose of anti-PD-1/anti-PD-L1 ICI, between December 2016 and January 2021, were included regardless of gender, age and performance status. ICI at any line of treatment for metastatic disease, alone or in combination with chemotherapy or another ICI, was allowed. Patients’ characteristics were abstracted from chart review. The following clinical characteristics were collected: age, gender, smoking status, comorbidities, primary tumour location and histology, type and line of ICI treatment, number and type of previous treatment lines received and sites of metastases. BMI at diagnosis, prior to ICI treatment and at progression was assessed. We selected three groups according to BMI at start of ICI: BMI <25, 25≤BMI<30 and BMI ≥30. We collected the following outcomes criteria: ICI overall response rate (ORR) and OS. ICI ORR was determined by investigator assessment of radiological response as per Response Evaluation Criteria in Solid Tumours (RECIST) and was the sum of complete response (CR) and partial response (PR). OS was calculated from the start of ICI treatment to death or last follow-up.

### Statistical analysis

Fisher’s exact test and χ^2^ test were used to assess differences in response rates between the predefined groups. OS was assessed using Kaplan-Meier method. Log-rank was used to compare groups and Cox regression models were used to perform univariable and multivariable analysis. P value <0.05 was considered statistically significant. Clinical variables that were associated with a p value <0.05 in the univariate analysis were included in the multivariable model. The final multivariable model included all variables with a p value <0.05 in the multivariate analysis. The survival analysis was performed with IBM SPSS V.23 (IBM Corp, Armonk, New York, USA). Multivariable analyses were performed with Stata Statistical Software/MP Release V.17, College Station, Texas: StataCorp LLC. The figures were created using the GraphPad Prism V.10.1.2 for Windows, GraphPad Software (www.graphpad.com).

### Patient and public involvement

Patients and/or the public were not involved in the design, conduct, reporting and dissemination plans of this research.

## Results

### Patients’ characteristics

We identified 135 patients, who received at least one dose of ICI for mUC. The median age was 70 years (26–91 years). Most of the patients had a primary bladder cancer (n=125, 92.6%). Most patients (n=84, 62%) received ICI as a second-line or later treatment for mUC. The median follow-up period was 14.5 months. Patients’ characteristics for the entire cohort are shown in table 1.

**Table 1:** Patients’ characteristics

Clinical characteristics	N (%)
Age	
≤60 years	28 (20.7%)
>60 years	107 (79.3%)
Gender	
Male	101 (74.8%)
Female	34 (25.2%)
Smoking status	
Ever smoker	69 (51.1%)
Never smoker	60 (44.4%)
Missing data	6 (4.5%)
Primary tumour site	
Bladder	125 (92.6%)
Upper tract	10 (7.4%)
Pathology	
Urothelial	123 (91.1%)
Squamous	7 (5.2%)
Other	5 (3.7%)
ECOG	
0–1	112 (83.0%)
≥2	15 (11.1%)
Missing data	8 (5.9%)
Metastatic sites*	
LN	90 (66.7%)
Lung	48 (35.6%)
Bone	43 (31.9%)
Liver	37 (27.4%)
BMI	
<25	55 (40.7%)
25–30	43 (31.9%)
≥30	23 (17.0%)
Missing data	14 (10.4%)
ICI line	
First	51 (37.8%)
Second or later	84 (62.2%)

*Numbers do not add up to 100% because many patients had multiple sites of metastases.

BMI, body mass index; ECOG, Eastern Cooperative Oncology Group Performance Status Scale; ICI, immune checkpoint inhibitor; LN, lymph node.

BMI data were available for 121 patients. A BMI ≥25 was observed in 48.8% of the patients. Median BMI was 20.9, 26.6 and 34.6 in groups BMI <25, 25≤BMI<30 and BMI ≥30, respectively. At the time ICI treatment was initiated, metastatic sites were found as follows: lymph nodes 66.7% (n=90), lung 35.6% (n=48), bones 31.9% (n=43) and liver 27.4% (n=37).

### The relationship between BMI and ICI response and survival outcomes

**Figure 1:**
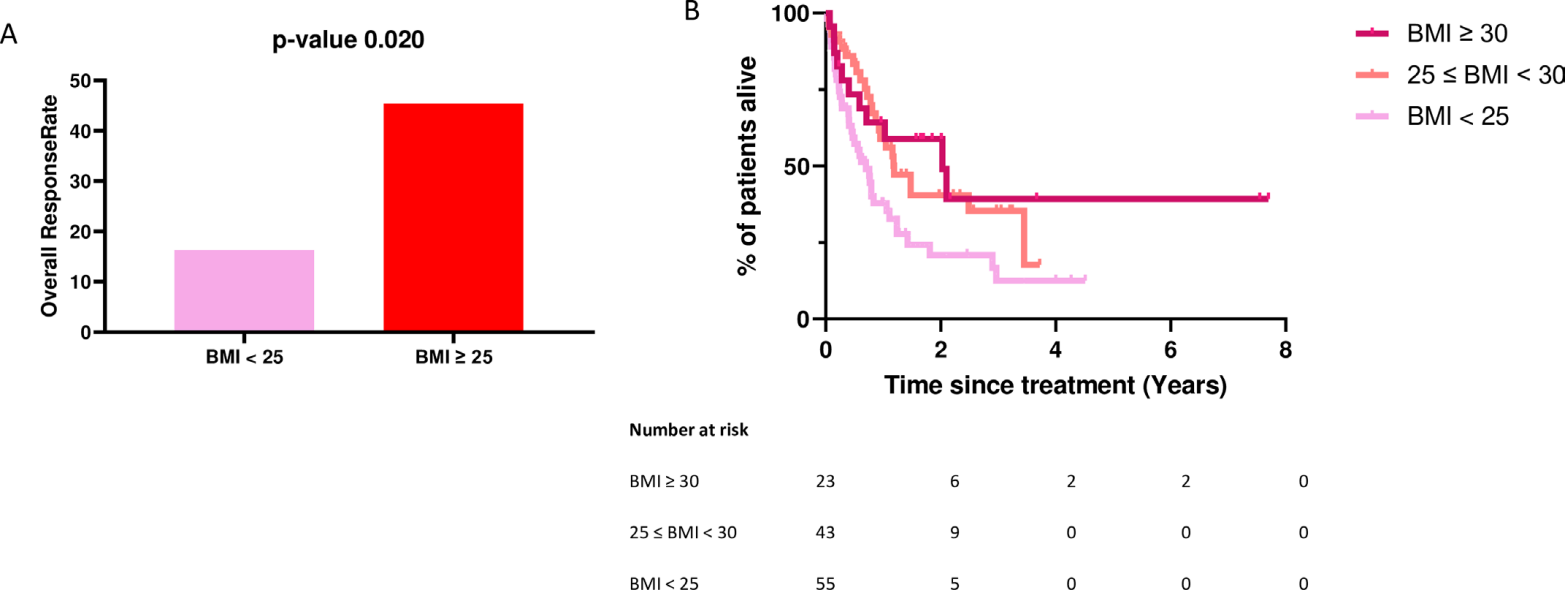
Overall response rate (A) and overall survival (B) according to BMI. BMI, body mass index.

We observed differences in ORR according to the BMI category of the patient. The ORR was 45.4% in the BMI ≥25 group, versus 16.3% in the BMI <25 group (p value=0.020) (figure 1A). In the BMI ≥25 and BMI <25 groups, we observed a higher proportion of CR to ICI (8 CR vs 1, 22 PR vs 8 and 7 SD vs 10) (online supplemental table 1). Patients with BMI ≥30 experienced the longest median OS (24.8 months) compared with those with 25≤BMI<30 (14.4 months) and BMI<25 (8.5 months) (p value=0.012). (figure 1B). BMI ≥30 remained an independent prognostic variable in multivariable analysis (HR=0.40, 95% CI: 0.17 to 0.96, p value=0.040) (table 2). These results were confirmed for patients treated with ICI alone (online supplemental figure 3A).

10.1136/bmjopen-2023-081480.supp4 Supplementary data

10.1136/bmjopen-2023-081480.supp3 Supplementary data

**Table 2:** Univariable and multivariable analysis for OS

OS	Univariable			Multivariable		
	HR	95% CI	P value	HR	95% CI	P value
BMI ≥30 vs <30	0.63	0.45 to 0.88	0.012	0.40	0.17 to 0.96	0.040
Male vs female	0.95	0.58 to 1.57	0.867			
Age ≥60 vs <60	1.88	0.65 to 5.45	0.235			
ECOG ≥2 vs 0–1	1.68	0.86 to 3.29	0.125	2.21	1.02 to 4.78	0.042
ICI line ≥2 vs 1	2.39	1.43 to 3.97	0.001	1.80	1.31 to 2.48	0.000
NLR ≥4 vs <4	2.46	1.53 to 3.94	0.000	2.66	1.58 to 4.49	0.000
Bone metastasis present vs absent	2.38	1.52 to 3.73	0.000	1.98	1.17 to 3.35	0.010
Lung metastasis present vs absent	1.88	1.21 to 2.93	0.005			
Liver metastasis present vs absent	1.92	1.2 to 3.09	0.006			

BMI, body mass index; ECOG, Eastern Cooperative Oncology Group Performance Status Scale; ICI, immune checkpoint inhibitor; NLR, neutrophil-to-lymphocyte ratio; OS, overall survival.

### Metastatic sites’ response and survival outcomes

**Figure 2:**
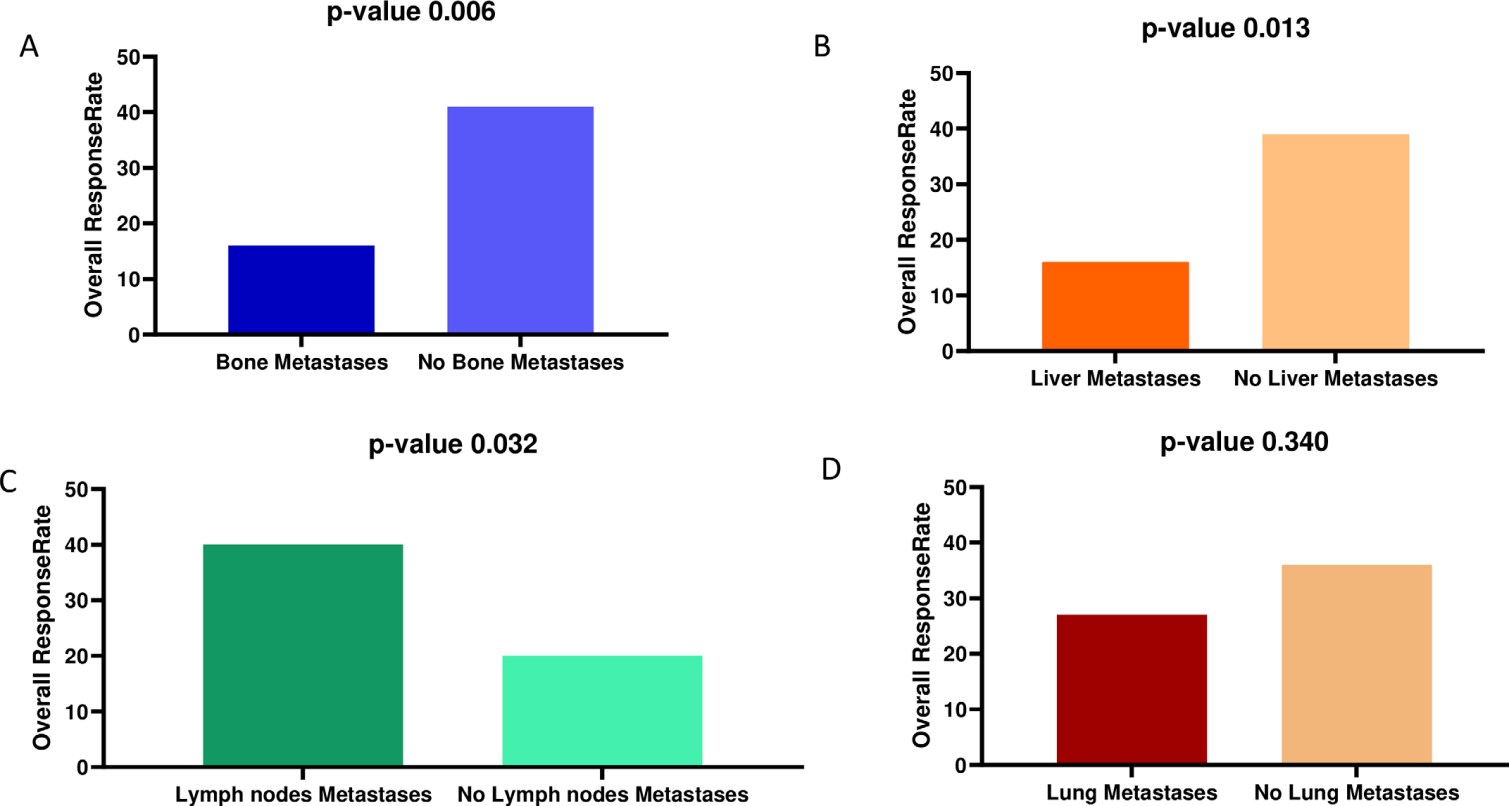
Overall response rate according to metastatic sites: (A) bone, (B) liver, (C) lymph nodes and (D) lung.

**Figure 3:**
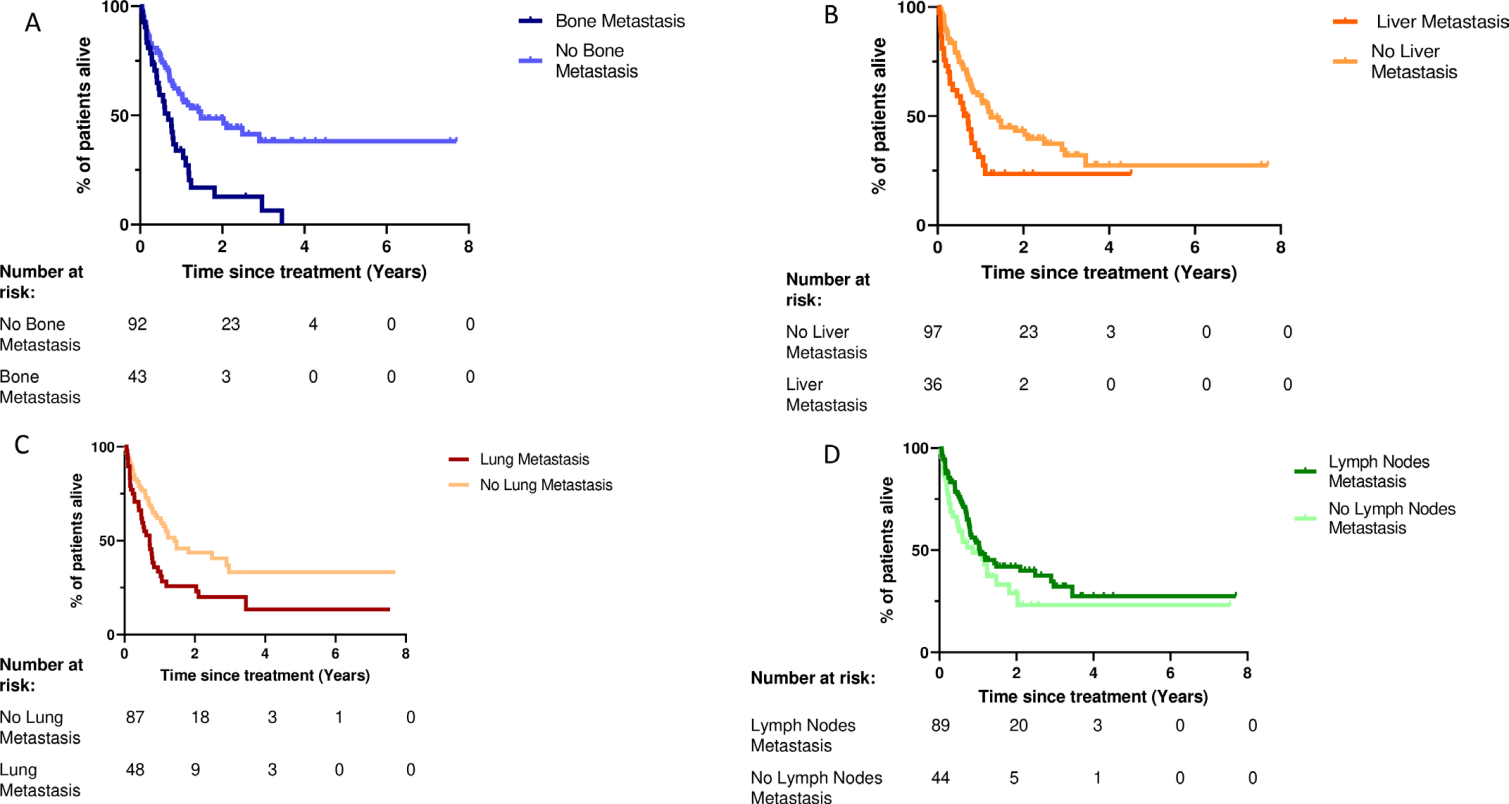
Overall survival according to metastatic sites: (A) bone, (B) liver, (C) lung and (D) lymph nodes.

The ORR for the entire cohort was 32.2%. The ORR was significantly lower in patients with bone metastases (16% vs 41%, p=0.006) (figure 2A, online supplemental figure 3B), or liver metastases (16% vs 39%, p value=0.013) (figure 2B). Conversely, the presence of metastatic disease involving the lymph nodes was significantly correlated with higher ORR (40% vs 20%, p value=0.032) (figure 2C). The difference in ORR for lung metastases was not statistically significant (27% vs 36%, p value=0.340) (figure 2D). Detailed responses are listed in online supplemental table 1. The median OS was evaluated for the entire cohort, for each metastatic site and for each BMI group. In the entire cohort, median OS was 12.7 months. Bone, liver and lung metastases correlated with significantly shorter survival. The median OS for patients with bone metastases was 7.3 months compared with 18 months in the absence of bone metastases (p value <0.001) (figure 3A, online supplemental figure 3B). Patients with liver metastases had a median OS of 8.6 months compared with 15 months (p value=0.006) (figure 3B), and 8.7 months compared with 17.3 months for those with lung metastases (p value=0.004) (figure 3C). The presence of lymph node metastases was not significantly associated with OS of 13.5 months versus 12.7 months (p value=0.175) (figure 3D). Although bone, lung and liver metastases were all associated with significantly worse OS in univariable analyses, bone metastases were the only metastatic site that remained significantly associated with shorter OS in multivariable analysis (HR=1.98, 95% CI: 1.17 to 3.35, p value=0.010) (table 2).

### NLR response and survival outcomes

Next, we evaluated the relationship between the NLR and clinical outcomes with ICI in this cohort. The ORR was not statistically different for patients with NLR ≥4 compared with those with NLR <4, respectively 24.5% and 36.9% (p value=0.141) (online supplemental figures 1 and 3 C). However, NLR <4 was correlated with better OS, with a median of 17.7 months versus 8.2 months for NLR ≥4 (p value <0.001) (online supplemental figures 2 and 3C). The survival benefit associated with a low NLR at the time of initiating ICI therapy was independent of other prognostic variable in multivariable analysis (table 2). We assessed whether there was a relationship between NLR and BMI, but we did not observe any significant correlation (data not shown).

10.1136/bmjopen-2023-081480.supp1 Supplementary data

10.1136/bmjopen-2023-081480.supp2 Supplementary data

## Discussion

ICIs are approved for a wide array of cancers based on tumour type (melanoma, NSCLC, urothelial cancer) and/or in a tumour type agnostic manner based on the presence of molecular biomarkers such as high tumour mutation burden^28–30^ and microsatellite instability.^30–32^ However, in most cases, many patients who are eligible for immunotherapies based on tumour type or biomarker status will either not respond to treatment or will eventually become resistant to treatment. In this analysis, we identified several important clinical biomarkers that associate with better (high BMI) or worse (bone metastases, high NLR) outcomes with ICI in patients with mUC. These biomarkers are easily assessed without any additional costly molecular testing. Moreover, ICIs are expensive, potentially toxic and in mUC only 30% of the patients benefit from ICIs. As such, we believe that these data may be clinically useful to guide treatment decisions.

According to the WHO obesity, classified as a BMI greater than 30 kg/m^2^, is a rising epidemic.^33^ Our data showed strong correlation between elevated BMI and improved outcome in terms of response as well as survival. To the best of our knowledge, the present study is the largest to report data of ICI outcomes correlation with BMI in mUC.^19 34 35^ A favourable prognostic role of high BMI was also reported in RCC, NSCLC and melanoma.^17 34 36–38^ A pooled post-hoc analysis of individual participant data from four prospective trials with Atezolizumab in metastatic NSCLC showed significant difference in survival between normal weight, overweight and obese patients with improved OS for patients with obesity and overweight compared with normal weight.^17^ In a retrospective multicohort analysis of 1918 patients with metastatic melanoma treated with chemotherapy, targeted therapies and ICI, McQuade *et al* studied association between BMI and outcome, as well as interactions between BMI and gender and type of treatment. Obesity was associated with improved OS and PFS with a benefit restricted to patients treated with targeted therapies and ICI.^16^

There is increasing evidence that excess body weight is a modifiable risk factor for several malignancies, including pancreas, kidney, colorectal, postmenopausal breast, ovarian, gallbladder and thyroid cancers.^39^ However, obesity is not an established risk factor for bladder cancer and the impact of obesity on cancer survival is less clear. Some studies have reported that obesity is associated with improved survival in some, but not all, patients with cancer, which is referred to the ‘obesity paradox’.^40^ Numerous studies have shown that obesity can cause immune cell dysfunction,^41–44^ while others argue that the chronic low-grade inflammation underpinning obesity creates a proinflammatory state that synergises with immunotherapy.^45 46^

Preclinical and translational research has provided insight into how immune checkpoint blockade can benefit obese patients. Obesity appears to influence T cell function and phenotype. For example, leptin-associated T cell dysfunction due to obesity has been observed across different species and tumour models and was found to enhance the immune response to anti-PD-1 therapy.^41^ In a mouse study, Shirakawa *et al* reported that obesity caused a preferential increase and accumulation of senescent CD44^hi^CD62L^lo^CD4+ T cells that constitutively express PD-1 and CD153.^47^ Moreover, a study in a colon carcinoma mouse model showed that obesity impaired the infiltration, cytokine production and metabolic activity of tumor-infiltrating CD8 T cells, while anti-PD-1 restored CD8 T cells infiltration, proinflammatory cytokine production and metabolic activity, leading to complete tumour eradication and immune memory.^48^ Obesity may also affect other immune cells in the tumour microenvironment (TME), such as myeloid-derived suppressor cells (MDSCs), which are known to suppress T cell responses. MDSCs can suppress T cell activity through many mechanisms, one of which is expressing PD-L1 to induce T cell exhaustion.^49 50^ In a preclinical study, inhibiting PD-L1 on MDSCs decreased their ability to suppress T cell activity, suggesting that obesity could also accelerate tumour progression by promoting the expression of PD-L1 on MDSCs.^51^ Finally, a preclinical study in a breast cancer model showed that although obesity accelerated tumour progression, anti-PD-1 treatment significantly reduced tumour burden by reshaping the local TME landscape.^52^

The relationship between obesity and ICI is complex and depends on various of factors, including the types of cancer and ICI involved. However, BMI may be an imperfect marker and its evaluation based only on weight prior to ICI start can be limiting. Assessment of the weight loss or change over the time may be a better indicator of disease prognosis. As BMI cannot distinguish body fat from muscle, it may not be the best tool to assess obesity.^53^ Other measurement methods could be used for better assessment such as dual energy X-ray absorptiometry or fat referenced quantitative MRI.^54^

Further studies are needed to understand the molecular mechanisms underlying the interaction between obesity and cancer immunity and to identify potential targets for effective interventions.

Bone metastases are a validated negative prognostic factor in mUC treated with platinum-based chemotherapy.^55 56^ In our study, poor response and survival outcome of bone and liver metastases was significant, while lymph nodes metastases correlated with higher ORR. These results are consistent with published data in the literature for patients treated with ICI.^57–59^ Makrakis *et al* showed lower response rates and shorter OS for bone and liver metastases on retrospective data from 917 mUC treated with ICI as first or ≥ second line, but higher ORR for lymph node-confined metastases.^57^ A retrospective multicentric Japanese study reported shorter ORR for bone metastases in a cohort of patients treated with Pembrolizumab for mUC.^58^ Similar results have also been reported from prospective trials.^60 61^ In the IMvigor210 phase II trial, the ORR on Atezolizumab was 32% for lymph node-confined metastases, and only 8% for liver metastases, data for bone metastases were not reported.^60^ Higher ORR for lymph nodes metastases (47%) compared with liver metastases (23%) was also reported in the KEYNOTE-052 trial.^61^ Prognostic models for patients with mUC treated with ICI identified liver or visceral metastases as poor prognostic factors.^62 63^ Sonpavde *et al* developed a five-factor prognostic model for survival for mUC treated with Atezolizumab, Avelumab or Durvalumab, based on the data from phase I and II trials.^64^ They identified three risk groups based on performance status, liver metastases, platelet count, NLR and lactate dehydrogenase (LDH) levels as prognostic factors.^64^ The 1-year Kaplan-Meier survival estimates of those in the low, intermediate and poor risk groups were 76.2%, 33.8% and 8.6%, respectively.^64^ The authors developed an interactive web-based tool to calculate the expected survival probabilities according to the five factors.^64^ In our study, LDH levels were not uniformly assessed prior to ICI treatment at all sites, and we did not collect data for platelet count. Therefore, we could not compare the prognostic utility of this tool in our cohort of patients. Most prospective trials of ICI in mUC do not use presence/absence of bone metastases as a stratification factor, despite the fact that bone metastases are associated with poor response to chemotherapy and impact quality of life.^55 56 65^ Owari *et al* validated a specific prognostic scoring system, B-FOM, to predict survival for patients with bone metastasis from different genitourinary cancers based on five prognostic factors: primary tumour (prostate, renal or urothelial cancers), poor performance status, visceral metastases, high Glasgow-prognostic score and elevated NLR.^66^ This prediction tool may be helpful to individualise optimal treatment strategy for the patients. A better understanding of the bone microenvironment is crucial to help the development of novel therapeutic strategies and improve outcomes.^67^

The distant organ microenvironment, also known as metastatic microenvironment (MME), plays an important role in site-specific metastases.^68^ For instance, macrophages, which are abundant immune cells in the lungs, can bind to cancer cells via receptor VCAM-1 transmit, signalling a chain of events leading to lung-specific metastases in breast cancer.^69^ It has been demonstrated that myeloid cells can remodel the premetastatic lung from an immune protective state to a state favouring tumour progression, thereby promoting lung metastases.^70^ Furthermore, lung stromal cells can also promote tumour colonisation and metastasis by secreting periostin.^71^ In the liver, there is a significant presence of circulating and resident natural killer (NK) cells, which serve as the primary effectors of liver immune function. This NK cell maintained the breast cancer cells dormant in the liver by secreting interferon-γ (IFN-γ), while sustaining NK cell abundance with interleukin-15 (IL-15)-based immunotherapy succeeded to prevent liver metastases and prolong the survival in preclinical models.^72^ Conversely, bone forms an immunosuppressive environment mainly due to immature NK cells, a small number of cytotoxic T cells, a large number of myeloid progenitors and Treg.^73^ In a human bladder tumour xenografts model, researchers observed a high infiltration of bone marrow-derived host CD11b myeloid cells. They further demonstrated that enhanced tumour-associated in the TME promote an immunosuppressive protumoral myeloid phenotype.^74^ These observations demonstrate how the MME can influence the metastatic potential and outcome of different types of cancer cells in different organs, suggesting that MME might also potentiate site-specific metastases in bladder cancer. Taken together, all these studies suggest the importance of understanding the metastatic potential and outcome of different types of cancer cells in different organs, through which we can further develop more effective therapeutic measures against bladder cancer.

NLR is a well-established poor prognostic factor in several cancers, independently of treatment type.^25–27^ It could be considered as a surrogate marker of chronic inflammation and evasion of immune surveillance.^75^ In our study, high NLR was associated with shorter median OS. A recent study by Valero *et al* showed poor response and survival outcomes, for 1917 patients with high NLR at diagnosis, treated with ICI for multiple cancer types.^75^ Similar results were reported for melanoma and NSCLC.^26 76^ Banna *et al* explored the prognostic and predictive role of NLR and LDH in mUCs treated with ICI and showed significant correlation with PFS and OS for high NLR.^77^ For patients treated with ICI for mUC, a high NLR prior to first-line chemotherapy and second-line pembrolizumab was associated with worse survival outcomes.^78^ NLR may represent an accessible low-cost predictive biomarker.

The present study has some strengths and limitations. It presents a relatively large multicentre cohort of patients with significant prognostic factors. On the other hand, this is a retrospective study, with heterogenous population and an investigator-based response assessment on clinical and radiological chart review. However, the observations made in this analysis are hypothesis generating. The evaluation of obesity and its relationship with ICI response warrants future prospective studies that incorporate better established measures of obesity and metabolic syndrome. Moreover, little is known about how bladder cancer cells interact with the liver and bone TME and this warrants further investigation in preclinical models to develop optimal immunotherapies for patients with bone and liver mUC.

## Conclusion

Our study identified elevated BMI, NLR and presence of bone metastases as potential biomarkers for ICI response and survival in mUC. Obesity was associated with improved survival and response rate, whereas bone metastases and high NLR are associated with lack of response and shorter survival. Prospective validation of these data is warranted, especially in the evolving landscape of therapeutic options for mUC with novel agents and combinations with ICI.^79^

## Supplementary Material

Reviewer comments

Author's manuscript

## Data Availability

All data relevant to the study are included in the article or uploaded as supplementary information.

## References

[1] Sung H , Ferlay J , Siegel RL , et al . Global cancer Statistics 2020: GLOBOCAN estimates of incidence and mortality worldwide for 36 cancers in 185 countries. CA Cancer J Clin 2021;71:209–49. 10.3322/caac.21660 33538338

[2] Humphrey PA , Moch H , Cubilla AL , et al . The 2016 WHO classification of tumours of the urinary system and male genital organs—part B: prostate and bladder tumours. Eur Urol 2016;70:106–19. 10.1016/j.eururo.2016.02.028 26996659

[3] Nadal R , Bellmunt J . Management of metastatic bladder cancer. Cancer Treat Rev 2019;76:10–21. 10.1016/j.ctrv.2019.04.002 31030123

[4] Witjes JA , Bruins HM , Cathomas R , et al . European Association of Urology guidelines on muscle-invasive and metastatic bladder cancer: summary of the 2020 guidelines. Eur Urol 2021;79:82–104. 10.1016/j.eururo.2020.03.055 32360052

[5] Powles T , Park SH , Voog E , et al . Avelumab maintenance therapy for advanced or metastatic urothelial carcinoma. N Engl J Med 2020;383:1218–30. 10.1056/NEJMoa2002788 32945632

[6] Bellmunt J , de Wit R , Vaughn DJ , et al . Pembrolizumab as second-line therapy for advanced urothelial carcinoma. N Engl J Med 2017;376:1015–26. 10.1056/NEJMoa1613683 28212060 PMC5635424

[7] McCaffrey JA , Hilton S , Mazumdar M , et al . Phase II trial of Docetaxel in patients with advanced or metastatic transitional-cell carcinoma. J Clin Oncol 1997;15:1853–7. 10.1200/JCO.1997.15.5.1853 9164195

[8] Dreicer R , Gustin DM , See WA , et al . Paclitaxel in advanced urothelial carcinoma: its role in patients with renal insufficiency and as salvage therapy. J Urol 1996;156:1606–8. 10.1016/s0022-5347(01)65459-4 8863548

[9] Ko Y-J , Canil CM , Mukherjee SD , et al . Nanoparticle albumin-bound paclitaxel for second-line treatment of metastatic urothelial carcinoma: a single group, Multicentre, phase 2 study. Lancet Oncol 2013;14:769–76. 10.1016/S1470-2045(13)70162-1 23706985

[10] Bellmunt J , Fougeray R , Rosenberg JE , et al . Long-term survival results of a randomized phase III trial of Vinflunine plus best supportive care versus best supportive care alone in advanced urothelial carcinoma patients after failure of platinum-based chemotherapy. Ann Oncol 2013;24:1466–72. 10.1093/annonc/mdt007 23419284

[11] Fradet Y , Bellmunt J , Vaughn DJ , et al . Randomized phase III KEYNOTE-045 trial of Pembrolizumab versus paclitaxel, Docetaxel, or Vinflunine in recurrent advanced urothelial cancer: results of >2 years of follow-up. Ann Oncol 2019;30:970–6. 10.1093/annonc/mdz127 31050707 PMC6594457

[12] Rosenberg JE , Hoffman-Censits J , Powles T , et al . Atezolizumab in patients with locally advanced and metastatic urothelial carcinoma who have progressed following treatment with platinum-based chemotherapy: a single-arm, Multicentre, phase 2 trial. Lancet 2016;387:1909–20. 10.1016/S0140-6736(16)00561-4 26952546 PMC5480242

[13] Powles T , Durán I , van der Heijden MS , et al . Atezolizumab versus chemotherapy in patients with platinum-treated locally advanced or metastatic urothelial carcinoma (Imvigor211): a Multicentre, open-label, phase 3 randomised controlled trial. Lancet 2018;391:748–57. 10.1016/S0140-6736(17)33297-X 29268948

[14] Sharma P , Retz M , Siefker-Radtke A , et al . Nivolumab in metastatic urothelial carcinoma after platinum therapy (Checkmate 275): a Multicentre, single-arm, phase 2 trial. Lancet Oncol 2017;18:312–22. 10.1016/S1470-2045(17)30065-7 28131785

[15] Sharma P , Siefker-Radtke A , de Braud F , et al . Nivolumab alone and with Ipilimumab in previously treated metastatic urothelial carcinoma: Checkmate 032 Nivolumab 1 mg/kg plus Ipilimumab 3 mg/kg expansion cohort results. J Clin Oncol 2019;37:1608–16. 10.1200/JCO.19.00538 31100038 PMC6879315

[16] McQuade JL , Daniel CR , Hess KR , et al . Association of body-mass index and outcomes in patients with metastatic Melanoma treated with targeted therapy, Immunotherapy, or chemotherapy: a retrospective, Multicohort analysis. Lancet Oncol 2018;19:310–22. 10.1016/S1470-2045(18)30078-0 29449192 PMC5840029

[17] Kichenadasse G , Miners JO , Mangoni AA , et al . Association between body mass index and overall survival with immune Checkpoint inhibitor therapy for advanced non-small cell lung cancer. JAMA Oncol 2020;6:512–8. 10.1001/jamaoncol.2019.5241 31876896 PMC6990855

[18] Albiges L , Hakimi AA , Xie W , et al . Body mass index and metastatic renal cell carcinoma: clinical and biological correlations. J Clin Oncol 2016;34:3655–63. 10.1200/JCO.2016.66.7311 27601543 PMC5065111

[19] Indini A , Rijavec E , Ghidini M , et al . Impact of BMI on survival outcomes of Immunotherapy in solid tumors: A systematic review. Int J Mol Sci 2021;22:2628. 10.3390/ijms22052628 33807855 PMC7961496

[20] Ganesh K , Massagué J . Targeting metastatic cancer. Nat Med 2021;27:34–44. 10.1038/s41591-020-01195-4 33442008 PMC7895475

[21] Oliver AJ , Lau PKH , Unsworth AS , et al . Tissue-dependent tumor Microenvironments and their impact on Immunotherapy responses. Front Immunol 2018;9:70. 10.3389/fimmu.2018.00070 29445373 PMC5797771

[22] Furubayashi N , Negishi T , Sakamoto N , et al . Organ-specific tumor response to Pembrolizumab in advanced urothelial carcinoma after platinum-based chemotherapy. Onco Targets Ther 2021;14:1981–8. 10.2147/OTT.S299724 33776447 PMC7987306

[23] Tumeh PC , Hellmann MD , Hamid O , et al . Liver metastasis and treatment outcome with anti-PD-1 Monoclonal antibody in patients with Melanoma and NSCLC. Cancer Immunol Res 2017;5:417–24. 10.1158/2326-6066.CIR-16-0325 28411193 PMC5749922

[24] Schmid S , Diem S , Li Q , et al . Organ-specific response to Nivolumab in patients with non-small cell lung cancer (NSCLC). Cancer Immunol Immunother 2018;67:1825–32. 10.1007/s00262-018-2239-4 30171269 PMC11028265

[25] Guthrie GJK , Charles KA , Roxburgh CSD , et al . The systemic inflammation-based neutrophil-lymphocyte ratio: experience in patients with cancer. Crit Rev Oncol Hematol 2013;88:218–30. 10.1016/j.critrevonc.2013.03.010 23602134

[26] Cedrés S , Torrejon D , Martínez A , et al . Neutrophil to lymphocyte ratio (NLR) as an indicator of poor prognosis in stage IV non-small cell lung cancer. Clin Transl Oncol 2012;14:864–9. 10.1007/s12094-012-0872-5 22855161

[27] Templeton AJ , McNamara MG , Šeruga B , et al . Prognostic role of neutrophil-to-lymphocyte ratio in solid tumors: a systematic review and meta-analysis. J Natl Cancer Inst 2014;106:dju124. 10.1093/jnci/dju124 24875653

[28] Kim SI , Cassella CR , Byrne KT . Tumor burden and Immunotherapy: impact on immune infiltration and therapeutic outcomes. Front Immunol 2020;11:629722. 10.3389/fimmu.2020.629722 33597954 PMC7882695

[29] Samstein RM , Lee C-H , Shoushtari AN , et al . Tumor mutational load predicts survival after Immunotherapy across multiple cancer types. Nat Genet 2019;51:202–6. 10.1038/s41588-018-0312-8 30643254 PMC6365097

[30] Palmeri M , Mehnert J , Silk AW , et al . Real-world application of tumor mutational burden-high (TMB-high) and Microsatellite instability (MSI) CONFIRMS their utility as Immunotherapy biomarkers. ESMO Open 2022;7:100336. 10.1016/j.esmoop.2021.100336 34953399 PMC8717431

[31] O’Malley DM , Bariani GM , Cassier PA , et al . Pembrolizumab in patients with Microsatellite instability-high advanced endometrial cancer: results from the KEYNOTE-158 study. J Clin Oncol 2022;40:752–61. 10.1200/JCO.21.01874 34990208 PMC8887941

[32] Maio M , Ascierto PA , Manzyuk L , et al . Pembrolizumab in Microsatellite instability high or mismatch repair deficient cancers: updated analysis from the phase II KEYNOTE-158 study. Ann Oncol 2022;33:929–38. 10.1016/j.annonc.2022.05.519 35680043

[33] Jensen MD , Ryan DH , Apovian CM , et al . AHA/ACC/TOS guideline for the management of overweight and obesity in adults: a report of the American college of cardiology/American heart Association task force on practice guidelines and the obesity society. Circulation 2014;129(25 Suppl 2):S102–38. 10.1161/01.cir.0000437739.71477.ee 24222017 PMC5819889

[34] Cortellini A , Bersanelli M , Buti S , et al . A multicenter study of body mass index in cancer patients treated with anti-PD-1/PD-L1 immune Checkpoint inhibitors: when overweight becomes favorable. J Immunother Cancer 2019;7:57. 10.1186/s40425-019-0527-y 30813970 PMC6391761

[35] Yoo S-K , Chowell D , Valero C , et al . Outcomes among patients with or without obesity and with cancer following treatment with immune Checkpoint blockade. JAMA Netw Open 2022;5:e220448. 10.1001/jamanetworkopen.2022.0448 35226089 PMC8886536

[36] Martini DJ , Kline MR , Liu Y , et al . Adiposity may predict survival in patients with advanced stage cancer treated with Immunotherapy in phase 1 clinical trials. Cancer 2020;126:575–82. 10.1002/cncr.32576 31648379

[37] Rutkowski P , Indini A , De Luca M , et al . Body mass index (BMI) and outcome of metastatic Melanoma patients receiving targeted therapy and Immunotherapy: a multicenter International retrospective study. J Immunother Cancer 2020;8:e001117. 10.1136/jitc-2020-001117 33203662 PMC7674105

[38] Richtig G , Hoeller C , Wolf M , et al . Body mass index may predict the response to Ipilimumab in metastatic Melanoma: an observational multi-centre study. PLOS ONE 2018;13:e0204729. 10.1371/journal.pone.0204729 30273398 PMC6166940

[39] Lauby-Secretan B , Scoccianti C , Loomis D , et al . Body fatness and cancer—viewpoint of the IARC working group. N Engl J Med 2016;375:794–8. 10.1056/NEJMsr1606602 27557308 PMC6754861

[40] Lennon H , Sperrin M , Badrick E , et al . The obesity paradox in cancer: a review. Curr Oncol Rep 2016;18:56. 10.1007/s11912-016-0539-4 27475805 PMC4967417

[41] Wang Z , Aguilar EG , Luna JI , et al . Paradoxical effects of obesity on T cell function during tumor progression and PD-1 Checkpoint blockade. Nat Med 2019;25:141–51. 10.1038/s41591-018-0221-5 30420753 PMC6324991

[42] Rebeles J , Green WD , Alwarawrah Y , et al . Obesity-induced changes in T-cell metabolism are associated with impaired memory T-cell response to influenza and are not reversed with weight loss. J Infect Dis 2019;219:1652–61. 10.1093/infdis/jiy700 30535161 PMC6473176

[43] Gibson JT , Orlandella RM , Turbitt WJ , et al . Obesity-associated myeloid-derived Suppressor cells promote apoptosis of tumor-infiltrating Cd8 T cells and Immunotherapy resistance in breast cancer. Front Immunol 2020;11:590794. 10.3389/fimmu.2020.590794 33123173 PMC7573510

[44] Ringel AE , Drijvers JM , Baker GJ , et al . Obesity shapes metabolism in the tumor Microenvironment to suppress anti-tumor immunity. Cell 2020;183:1848–66. 10.1016/j.cell.2020.11.009 33301708 PMC8064125

[45] Wang Z , Monjazeb AM , Murphy WJ . The complicated effects of obesity on cancer and Immunotherapy. Immunotherapy 2019;11:11–4. 10.2217/imt-2018-0133 30702013

[46] Fang H , Wu Y , Chen L , et al . Regulating the obesity-related tumor Microenvironment to improve cancer Immunotherapy. ACS Nano 2023;17:4748–63. 10.1021/acsnano.2c11159 36809912

[47] Shirakawa K , Yan X , Shinmura K , et al . Obesity accelerates T cell Senescence in murine visceral Adipose tissue. J Clin Invest 2016;126:4626–39. 10.1172/JCI88606 27820698 PMC5127667

[48] Dyck L , Prendeville H , Raverdeau M , et al . Suppressive effects of the obese tumor Microenvironment on Cd8 T cell infiltration and Effector function. J Exp Med 2022;219:e20210042. 10.1084/jem.20210042 35103755 PMC8932531

[49] Lau J , Cheung J , Navarro A , et al . Tumour and host cell PD-L1 is required to mediate suppression of anti-tumour immunity in mice. Nat Commun 2017;8:14572. 10.1038/ncomms14572 28220772 PMC5321797

[50] Weber R , Fleming V , Hu X , et al . Myeloid-derived Suppressor cells hinder the anti-cancer activity of immune Checkpoint inhibitors. Front Immunol 2018;9:1310. 10.3389/fimmu.2018.01310 29942309 PMC6004385

[51] Hale M , Itani F , Buchta CM , et al . Obesity triggers enhanced MDSC accumulation in murine renal tumors via elevated local production of Ccl2. PLoS One 2015;10:e0118784. 10.1371/journal.pone.0118784 25769110 PMC4358922

[52] Pingili AK , Chaib M , Sipe LM , et al . Immune Checkpoint blockade Reprograms systemic immune landscape and tumor Microenvironment in obesity-associated breast cancer. Cell Rep 2021;35:109285. 10.1016/j.celrep.2021.109285 34161764 PMC8574993

[53] Arnett DK , Blumenthal RS , Albert MA , et al . ACC/AHA guideline on the primary prevention of cardiovascular disease: A report of the American college of cardiology/American heart Association task force on clinical practice guidelines. J Am Coll Cardiol 2019;74:e177–232. 10.1016/j.jacc.2019.03.010 30894318 PMC7685565

[54] Borga M , West J , Bell JD , et al . Advanced body composition assessment: from body mass index to body composition profiling. J Investig Med 2018;66:1–9. 10.1136/jim-2018-000722 PMC5992366 29581385

[55] Alqaisi HA , Stecca C , Veitch ZW , et al . The Prognostic impact of bone metastasis in patients with metastatic urothelial carcinoma treated with first-line platinum-based chemotherapy. Ther Adv Med Oncol 2022;14:17588359221094879. 10.1177/17588359221094879 35520101 PMC9066632

[56] Necchi A , Sonpavde G , Lo Vullo S , et al . Nomogram-based prediction of overall survival in patients with metastatic urothelial carcinoma receiving first-line platinum-based chemotherapy: retrospective international study of invasive/advanced cancer of the Urothelium (RISC). Eur Urol 2017;71:281–9. 10.1016/j.eururo.2016.09.042 27726966 PMC5576985

[57] Makrakis D , Talukder R , Lin GI , et al . Association between sites of metastasis and outcomes with immune Checkpoint inhibitors in advanced urothelial carcinoma. Clin Genitourin Cancer 2022;20:e440–52. 10.1016/j.clgc.2022.06.001 35778337 PMC10257151

[58] Shimizu T , Miyake M , Nishimura N , et al . Organ-specific and mixed responses to Pembrolizumab in patients with Unresectable or metastatic urothelial carcinoma: A multicenter retrospective study. Cancers (Basel) 2022;14:1735. 10.3390/cancers14071735 35406508 PMC8997142

[59] Raggi D , Giannatempo P , Marandino L , et al . Role of bone metastases in patients receiving Immunotherapy for pre-treated urothelial carcinoma: the Multicentre, retrospective meet-URO-1 bone study. Clin Genitourin Cancer 2022;20:155–64. 10.1016/j.clgc.2021.12.008 35000876

[60] Balar AV , Galsky MD , Rosenberg JE , et al . Atezolizumab as first-line treatment in cisplatin-ineligible patients with locally advanced and metastatic urothelial carcinoma: a single-arm, Multicentre, phase 2 trial. Lancet 2017;389:67–76. 10.1016/S0140-6736(16)32455-2 27939400 PMC5568632

[61] Balar AV , Castellano D , O’Donnell PH , et al . First-line Pembrolizumab in cisplatin-ineligible patients with locally advanced and Unresectable or metastatic urothelial cancer (KEYNOTE-052): a Multicentre, single-arm, phase 2 study. Lancet Oncol 2017;18:1483–92. 10.1016/S1470-2045(17)30616-2 28967485

[62] Nassar AH , Mouw KW , Jegede O , et al . A model combining clinical and Genomic factors to predict response to PD-1/PD-L1 blockade in advanced urothelial carcinoma. Br J Cancer 2020;122:555–63. 10.1038/s41416-019-0686-0 31857723 PMC7028947

[63] Khaki AR , Li A , Diamantopoulos LN , et al . A new Prognostic model in patients with advanced urothelial carcinoma treated with first-line immune Checkpoint inhibitors. Eur Urol Oncol 2021;4:464–72. 10.1016/j.euo.2020.12.006 33423945 PMC8169524

[64] Sonpavde G , Manitz J , Gao C , et al . Five-factor Prognostic model for survival of post-platinum patients with metastatic urothelial carcinoma receiving PD-L1 inhibitors. J Urol 2020;204:1173–9. 10.1097/JU.0000000000001199 32552295 PMC7655635

[65] Stellato M , Santini D , Cursano MC , et al . Bone metastases from urothelial carcinoma. the dark side of the moon. J Bone Oncol 2021;31:100405. 10.1016/j.jbo.2021.100405 34934613 PMC8661045

[66] Owari T , Miyake M , Nakai Y , et al . External validation of a Genitourinary cancer-specific Prognostic scoring system to predict survival for patients with bone metastasis (modified B-FOM scoring model): comparison with other scoring models in terms of accuracy. J Bone Oncol 2021;26:100344. 10.1016/j.jbo.2020.100344 33384916 PMC7770480

[67] Reinstein ZZ , Pamarthy S , Sagar V , et al . Overcoming immunosuppression in bone metastases. Crit Rev Oncol Hematol 2017;117:114–27. 10.1016/j.critrevonc.2017.05.004 28600175

[68] Liu Q , Zhang H , Jiang X , et al . “Factors involved in cancer metastasis: a better understanding to “seed and soil” hypothesis”. Mol Cancer 2017;16:176. 10.1186/s12943-017-0742-4 29197379 PMC5712107

[69] Chen Q , Zhang XH-F , Massagué J . Macrophage binding to receptor VCAM-1 transmits survival signals in breast cancer cells that invade the lungs. Cancer Cell 2011;20:538–49. 10.1016/j.ccr.2011.08.025 22014578 PMC3293160

[70] Yan HH , Pickup M , Pang Y , et al . Gr-1+ Cd11B+ myeloid cells tip the balance of immune protection to tumor promotion in the Premetastatic lung. Cancer Res 2010;70:6139–49. 10.1158/0008-5472.CAN-10-0706 20631080 PMC4675145

[71] Malanchi I , Santamaria-Martínez A , Susanto E , et al . Interactions between cancer stem cells and their niche govern metastatic Colonization. Nature 2012;481:85–9. 10.1038/nature10694 22158103

[72] Correia AL , Guimaraes JC , Auf der Maur P , et al . Hepatic Stellate cells suppress NK cell-sustained breast cancer dormancy. Nature 2021;594:566–71. 10.1038/s41586-021-03614-z 34079127

[73] Zhao E , Xu H , Wang L , et al . Bone marrow and the control of immunity. Cell Mol Immunol 2012;9:11–9. 10.1038/cmi.2011.47 22020068 PMC3251706

[74] Eruslanov E , Daurkin I , Vieweg J , et al . Aberrant Pge2 metabolism in bladder tumor Microenvironment promotes immunosuppressive phenotype of tumor-infiltrating myeloid cells. Int Immunopharmacol 2011;11:848–55. 10.1016/j.intimp.2011.01.033 21315786 PMC3241976

[75] Valero C , Lee M , Hoen D , et al . Pretreatment neutrophil-to-lymphocyte ratio and mutational burden as biomarkers of tumor response to immune Checkpoint inhibitors. Nat Commun 2021;12:729. 10.1038/s41467-021-20935-9 33526794 PMC7851155

[76] Capone M , Giannarelli D , Mallardo D , et al . Baseline neutrophil-to-lymphocyte ratio (NLR) and derived NLR could predict overall survival in patients with advanced Melanoma treated with Nivolumab. J Immunother Cancer 2018;6:74. 10.1186/s40425-018-0383-1 30012216 PMC6048712

[77] Banna GL , Di Quattro R , Malatino L , et al . Neutrophil-to-lymphocyte ratio and lactate dehydrogenase as biomarkers for urothelial cancer treated with Immunotherapy. Clin Transl Oncol 2020;22:2130–5. 10.1007/s12094-020-02337-3 32232716

[78] Kobayashi T , Ito K , Kojima T , et al . Pre-Pembrolizumab neutrophil-to-lymphocyte ratio (NLR) predicts the efficacy of second-line Pembrolizumab treatment in urothelial cancer regardless of the pre-Chemo NLR. Cancer Immunol Immunother 2022;71:461–71. 10.1007/s00262-021-03000-8 34235546 PMC10992102

[79] Hoimes CJ , Flaig TW , Milowsky MI , et al . Enfortumab Vedotin plus Pembrolizumab in previously untreated advanced urothelial cancer. J Clin Oncol 2023;41:22–31. 10.1200/JCO.22.01643 36041086 PMC10476837

