# Supplementary material for: Clinical variables associated with immune checkpoint inhibitor outcomes in patients with metastatic urothelial carcinoma: a multicentre retrospective cohort study
Source: BMJ Open. 2024 Mar 29;14(3):e081480. doi: 10.1136/bmjopen-2023-081480 (PMC10982788; doi:10.1136/bmjopen-2023-081480)
Supplement: Supplementary data [file bmjopen-2023-081480supp004.pdf]

	BMI[a] ≥ 25	BMI[a]< 25	Lymphnode	Lung	Bone	Liver
ORR[b] (CR[c]+PR[d]), %	45.4	16.3	40	27	16	16
CR, n (%)	8 (12.1)	1 (1.8)	7 (7.77%)	2 (4.16%)	1 (2.1%)	1 (2.5%)
PR, n (%)	22 (33.3)	8 (14.5)	29 (32.2%)	11 (22.9%)	6 (13.9%)	5 (13.5%)
SD[e], n (%)	7 (10.6)	10 (18.1)	10 (11.1%)	3 (6.25%)	6 (13.9%)	3 (8.1%)
PD[f], n (%)	24 (43.9)	36 (65.4)	44 (48.8%)	32 (66.6%)	30 (69.7%)	28 (75.6%)

[a]Body mass index [b]Overall response rate [c]Complete response [d]Partial response [e]Stable disease [f]Progressive disease

Supplemental Table: Responses according to clinical variables [a]BMI and metastatic sites
