# Supplementary material for: Clinical variables associated with immune checkpoint inhibitor outcomes in patients with metastatic urothelial carcinoma: a multicentre retrospective cohort study
Source: BMJ Open. 2024 Mar 29;14(3):e081480. doi: 10.1136/bmjopen-2023-081480 (PMC10982788; doi:10.1136/bmjopen-2023-081480)
Supplement: Supplementary data [file bmjopen-2023-081480supp003.pdf]

BMJ Publishing Group Limited (BMJ) disclaims all liability and responsibility arising from any reliance placed on this supplemental material which has been supplied by the author(s)

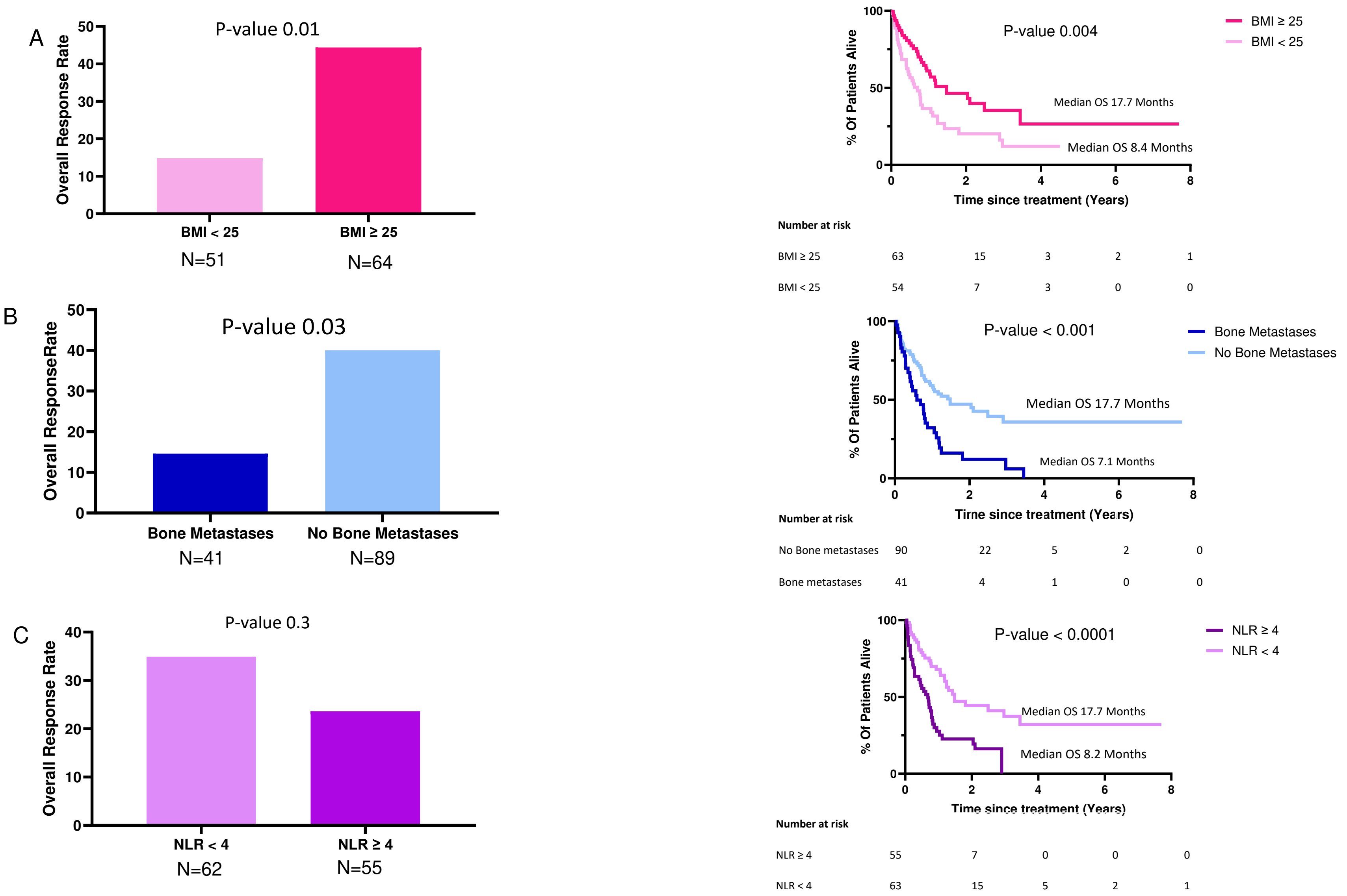

Supplemental Figure 3 ORR and OS according to BMI (A) Bone metastases (B) and NLR (C) in the cohort of patients treated with IO alone
