# Supplementary figures and images for: Clinical variables associated with immune checkpoint inhibitor outcomes in patients with metastatic urothelial carcinoma: a multicentre retrospective cohort study
Source: BMJ Open. 2024 Mar 29;14(3):e081480. doi: 10.1136/bmjopen-2023-081480 (PMC10982788; doi:10.1136/bmjopen-2023-081480)

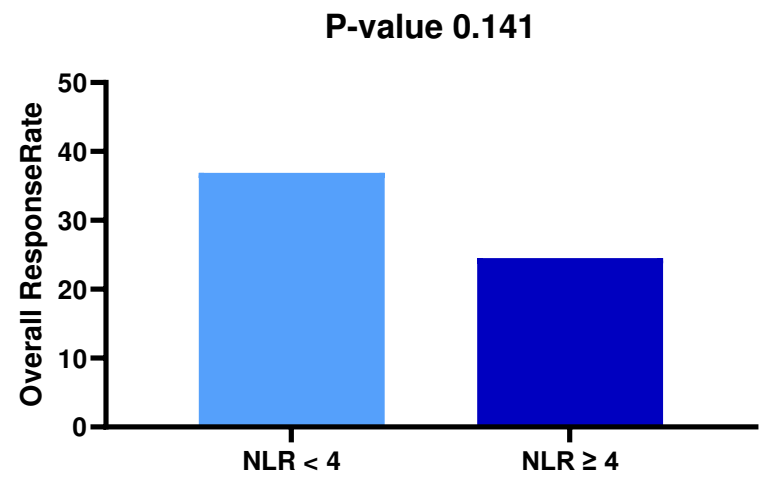

Supplemental Figure 1 Overall response rate according to neutrophil-to-lymphocytes ratio (NLR)

Supplement: Supplementary data [file bmjopen-2023-081480supp001.pdf]

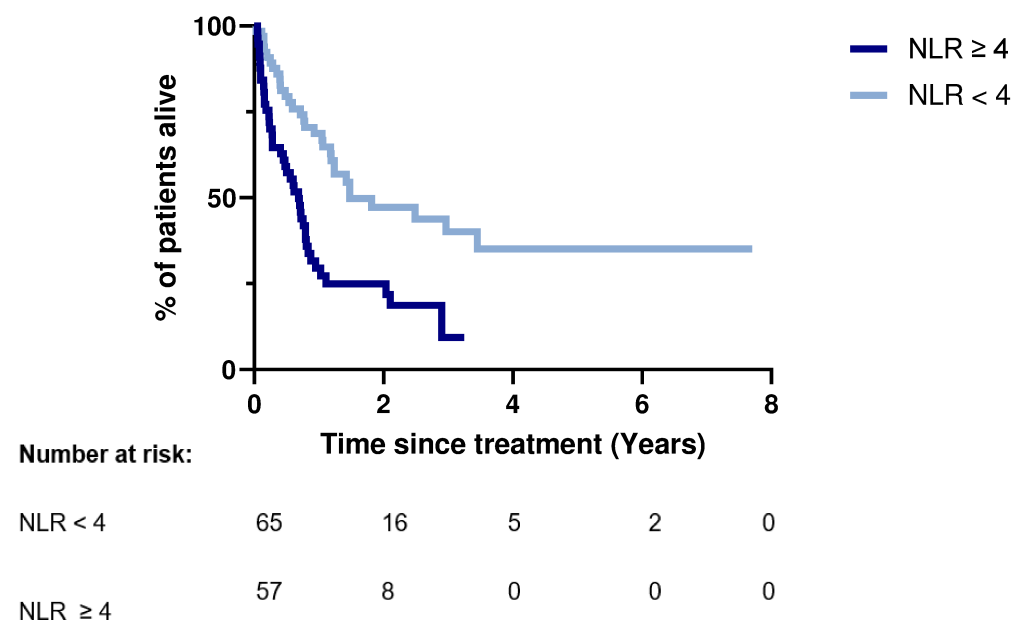

Supplemental Figure 2 Overall survival according to NLR

Supplement: Supplementary data [file bmjopen-2023-081480supp002.pdf]
