# Supplementary material for: Clinical variables associated with immune checkpoint inhibitor outcomes in patients with metastatic urothelial carcinoma: a multicentre retrospective cohort study
Source: BMJ Open. 2024 Mar 29;14(3):e081480. doi: 10.1136/bmjopen-2023-081480 (PMC10982788; doi:10.1136/bmjopen-2023-081480)
Supplement: Author's manuscript [file bmjopen-2023-081480.draft_revisions.pdf]

BMJ Open is committed to open peer review. As part of this commitment we make the peer review history of every article we publish publicly available.

When an article is published we post the peer reviewers' comments and the authors' responses online. We also post the versions of the paper that were used during peer review. These are the versions that the peer review comments apply to.

The versions of the paper that follow are the versions that were submitted during the peer review process. They are not the versions of record or the final published versions. They should not be cited or distributed as the published version of this manuscript.

BMJ Open is an open access journal and the full, final, typeset and author-corrected version of record of the manuscript is available on our site with no access controls, subscription charges or pay-per-view fees (http://bmjopen.bmj.com).

If you have any questions on BMJ Open's open peer review process please email info.bmjopen@bmj.com

BMJ Open

Clinical variables associated with immune checkpoint inhibitor outcomes in patients with metastatic urothelial carcinoma

Journal:	*BMJ Open*
Manuscript ID	bmjopen-2023-081480
Article Type:	Original research
Date Submitted by the Author:	28-Oct-2023

Complete List of Authors:	Labidi, Soumaya; Jewish General Hospital<br>Meti, Nicholas; McGill University<br>Barua, Reeta; Toronto East Health Network Michael Garron Hospital<br>Li, Mengqi; Lady Davis Institute for Medical Research; McGill University<br>Riromar, Jamila; The Royal Hospital, National Oncology Center; University Health Network<br>Jiang, Di Maria; University Health Network; University of Toronto<br>Fallah-Rad, Nazanin; University Health Network; University of Toronto<br>Sridhar, Srikala ; Princess Margaret Hospital Cancer Centre, Medical Oncology<br>Del Rincon, Sonia V.; Lady Davis Institute for Medical Research; McGill University, Oncology<br>Pezo, Rossanna C.; Sunnybrook Health Sciences Centre; University of Toronto<br>Ferrario, Cristiano; Jewish General Hospital; McGill University<br>Cheng, Susanna; Sunnybrook Health Sciences Centre; University of Toronto<br>Sacher, Adrian; Princess Margaret Hospital Cancer Centre, Medical Oncology<br>Rose, April; Jewish General Hospital, Segal Cancer Centre; McGill University, Oncology

Keywords:	Urological tumours < ONCOLOGY, Adult oncology < ONCOLOGY, Clinical Reasoning, EPIDEMIOLOGY, ONCOLOGY

SCHOLARONE™
Manuscripts

I, the Submitting Author has the right to grant and does grant on behalf of all authors of the Work (as defined in the below author licence), an exclusive licence and/or a non-exclusive licence for contributions from authors who are: i) UK Crown employees; ii) where BMJ has agreed a CC-BY licence shall apply, and/or iii) in accordance with the terms applicable for US Federal Government officers or employees acting as part of their official duties; on a worldwide, perpetual, irrevocable, royalty-free basis to BMJ Publishing Group Ltd ("BMJ") its licensees and where the relevant Journal is co-owned by BMJ to the co-owners of the Journal, to publish the Work in this journal and any other BMJ products and to exploit all rights, as set out in our licence.

The Submitting Author accepts and understands that any supply made under these terms is made by BMJ to the Submitting Author unless you are acting as an employee on behalf of your employer or a postgraduate student of an affiliated institution which is paying any applicable article publishing charge ("APC") for Open Access articles. Where the Submitting Author wishes to make the Work available on an Open Access basis (and intends to pay the relevant APC), the terms of reuse of such Open Access shall be governed by a Creative Commons licence – details of these licences and which Creative Commons licence will apply to this Work are set out in our licence referred to above.

Other than as permitted in any relevant BMJ Author's Self Archiving Policies, I confirm this Work has not been accepted for publication elsewhere, is not being considered for publication elsewhere and does not duplicate material already published. I confirm all authors consent to publication of this Work and authorise the granting of this licence.

For peer review only

Clinical variables associated with immune checkpoint inhibitor outcomes in patients with metastatic urothelial carcinoma

Soumaya Labidi[1,2], Nicholas Meti[3], Reeta Barua[4], Mengqi Li[5,6], Jamila Riromar[7], Di Maria Jiang[8], Nazanin Fallah-Rad[8], Srikala S. Sridhar[8], Sonia V. del Rincon[1,5,6], Rossanna C. Pezo[9], Cristiano Ferrario[1,2], Susanna Cheng[9], Adrian G. Sacher[8]*, April A. N. Rose[1,2,5,6]*

1. Gerald Bronfman Department of Oncology, McGill University, Montréal, QC. Canada
2. Segal Cancer Center, Jewish General Hospital. Montréal, QC, Canada
3. St. Mary's Hospital, Montréal, QC. Canada
4. Michael Garron Hospital. Toronto East Health Network. Toronto, ON. Canada
5. Lady Davis Institute, Jewish General Hospital, Montréal, QC, Canada
6. Division of Experimental Medicine, Faculty of Medicine, McGill University, Montréal, QC, Canada
7. Royal Hospital National Oncology Center, Sultanat, Oman
8. Princess Margaret Cancer Center. Toronto, ON, Canada
9. Odette Cancer Center. Sunnybrook Health Sciences Center. Toronto, ON, Canada

*Equal contribution

Corresponding Author:

April Rose, MD, PhD, FRCPC

3755 Chemin de la Côte-Sainte-Catherine

Office E-444

Montreal, QC, Canada H3T 1E2

Phone: (514) 340-8222 xt 27343

Email: april.rose@mcgill.ca

Running head: Immunotherapy outcomes in urothelial cancer

For peer review only - http://bmjopen.bmj.com/site/about/guidelines.xhtml

Abstract

Objectives: Immune checkpoint inhibitors (ICI) are indicated for metastatic urothelial cancer (mUC). Predictive and prognostic factors are lacking. We investigated clinical variables associated with ICI outcomes.

Methods: We performed a multicenter retrospective cohort study of 135 patients who received ICI for mUC, 2016-2021, at 3 Canadian centres. Clinical characteristics, BMI, metastatic sites, neutrophil-to-lymphocyte ratio (NLR), response, and survival were abstracted from chart review.

Results: A BMI ≥ 25 was significantly correlated to a higher overall response rate (ORR) (45.4% vs 16.3%, P = 0.020). Patients with BMI ≥ 30 experienced longer median OS 24.8 vs. 14.4 for 25 ≤ BMI < 30 and 8.5 months for BMI < 25 (P = 0.012). The ORR was lower in the presence of bone metastasis 16% vs 41% P = 0.006, and liver metastasis 16% vs 39% P = 0.013. Metastatic lymphnodes was correlated with higher ORR 40% vs 20% P = 0.032. The median OS for bone metastasis was 7.3 vs 18 months (P < 0.001). Patients with liver metastasis had a median OS of 8.6 vs 15 months (P = 0.006). No difference for lymph nodes metastasis (13.5 vs 12.7 months P = 0.175). NLR ≥ 4 had worse OS (8.2 vs 17.7 months P = 0.0001). In multivariate analysis, BMI ≥ 30, bone metastasis and NLR ≥ 4 were independent factors for OS.

Conclusions: Our data identified BMI and bone metastasis as novel clinical biomarkers that were independently associated with ICI outcomes in mUC. External and prospective validation are warranted.

For peer review only - http://bmjopen.bmj.com/site/about/guidelines.xhtml

Introduction

Bladder cancer represents the 10th most common cancer in the world, with approximately 550000 new cases annually, and accounts for 2.1% of all cancer deaths according to GLOBOCAN 2018 [1]. There is a male predominance, and smoking is the most common risk factor [1, 2]. Urothelial carcinomas arise from the urothelium of the bladder in most cases but can also develop from the upper urinary tract (renal pelvis and ureters). Tumors invading the detrusor muscle, ie., muscle invasive tumors, account with upfront metastatic disease for 25% of the cases [3]. Despite multimodality management for non-metastatic muscle invasive bladder cancer, 50% of these patients will relapse, with distant metastasis in most of the cases [4, 5]. Platinum-based chemotherapy remains the standard first line treatment for metastatic disease [4, 5] . Patients who respond or have stable disease following chemotherapy are eligible for subsequent maintenance therapy with the anti-PD-L1 immune checkpoint inhibitor, Avelumab [6], while those who fail to respond to chemotherapy subsequently can receive the anti-PD-1 immune checkpoint inhibitor, Pembrolizumab [7]. Other treatment options in the platinum-refractory setting include Enfortumab Vedotin, Erdafitinib in patients with susceptible FGFR alterations, and chemotherapy with taxanes (Paclitaxel, Docetaxel) or Vinflunine [8-11]. In the KEYNOTE-045 randomized phase 3 trial, Pembrolizumab showed a significantly longer overall survival of 10.3 months vs 7.4 months in patients with platinum refractory mUC compared to chemotherapy (Docetaxel, Paclitaxel, Vinflunine) (HR 0.73; 95%CI, 0.59 to 0.91 P = 0.002), with a lower rate of any grade adverse events (60.9% vs 90.2%) [7]. Long term results confirmed an overall survival benefit and a favorable safety profile [12]. The PD-L1 checkpoint inhibitor Atezolizumab was also associated with durable objective responses in a phase 2 trial, with favorable safety profile, [13] but failed to show survival benefit over chemotherapy in a phase 3 trial [14]. In phase 1/2 trials, Nivolumab, a PD-1 inhibitor, achieved objective responses alone or in combination with Ipilimumab, with a

For peer review only - http://bmjopen.bmj.com/site/about/guidelines.xhtml

manageable adverse events profile [15, 16]. Despite responses seen with ICI in the platinum-refractory setting, not all patients will derive benefit, and there is a lack of valid prognostic and predictive biomarkers.

In this study, we aimed to evaluate if clinical prognostic markers, that have been defined for other cancer types, are relevant to mUC. In fact, several retrospective studies have addressed the relationship between obesity and outcomes in patients receiving ICI. In melanoma, non-small cell lung cancer (NSCLC) and renal cell cancer (RCC), obesity appears to be linked to better PFS and OS.[17] [18, 19]. And a recent systematic review of 18 retrospective studies across different cancer types, also found an association between high BMI and improved ICI outcomes; yet a strong positive correlation could not be concluded due to the heterogeneity of the studies [20]. As such, the relationship between BMI and immunotherapy outcomes in urothelial cancer remains poorly understood.

The tumor immune microenvironment of different metastatic locations may also affect the response to ICI [21, 22]. Retrospective studies reported differences in organ specific responses and organ specific overall survival with immunotherapy in several types of cancer [23-25].

Neutrophil-to-lymphocyte ratio (NLR) is an available marker of systemic response to inflammation, derived from the absolute counts of neutrophils and lymphocytes on a blood count [26]. It is a well-established poor prognostic factor in several cancers, independently of treatment type [26-28].

The aim of this retrospective analysis was to assess the association between high BMI, site of metastasis, NLR and outcome in terms of response and overall survival in a population of patients with mUC treated with ICI.

Patients and methods

Patient population, characteristics, and outcome

For peer review only - http://bmjopen.bmj.com/site/about/guidelines.xhtml

We performed a multi-center retrospective cohort study analysis of mUC patients, from 3 Canadian cancer centers: Segal Cancer Center, Jewish General Hospital (JGH); Princess Margaret Cancer Center, University Health Network (PM-UHN) and Odette Cancer Center, Sunnybrook Health Sciences Center (SHSC). Patients with histologically proven metastatic urothelial cancer, who received at least one dose of anti-PD1/L1 ICI, between December 2016 and January 2021, were included regardless of gender, age, and performance status. ICI at any line of treatment for metastatic disease, alone or in combination with chemotherapy or another ICI, was allowed. Patients' characteristics were abstracted from chart review. The following clinical characteristics were collected: age, gender, smoking status, comorbidities, primary tumor location and histology, type and line of ICI treatment, number and type of previous lines received, and sites of metastases. BMI at diagnosis, prior to ICI treatment and at progression was assessed. We selected 3 groups according to BMI at start of ICI: BMI < 25, $25 \leq$ BMI $<$ 30 and BMI ≥ 30. We collected the following outcomes criteria: ICI overall response rate (ORR), and overall survival (OS). ICI ORR was determined by investigator assessment of radiologic response as per RECIST criteria and was the sum of complete (CR) and partial response (PR). OS was calculated from the start of ICI treatment to death or last follow-up.

Ethics approval

Ethics approval for this study was obtained from the Clinical Trials Ontario (CTO Project ID: 2067) after review by the University Health Network Research Ethics Board on August 19, 2020, for PM-UHN and SHSC sites. Ethics approval for the JGH site was approved by the CIUSSS West Central Montreal REB (Project 2022-2888) on October 28, 2022.

Statistical analysis

Fisher's exact test and chi square test were used to assess differences in response rates between the predefined groups. OS was assessed using Kaplan Meier. Log rank was used to compare

For peer review only - http://bmjopen.bmj.com/site/about/guidelines.xhtml

groups and Cox regression models were used to perform univariable and multivariable analysis. The cut-off significant p value $P < 0.05$. Factors in the univariate analysis with a p value <0.05 and p value between [0.05-0.2] we included in the multivariate analysis.

Results

Patients' characteristics

We identified 135 patients, who received at least one dose of ICI for mUC. The median age was 70 years (26-91). Most of the patients had a primary bladder cancer (92.5%). Most patients (n=84, 62%) received ICI as a second line or later treatment for mUC. The median follow-up period was 14.5 months. Patients' characteristics for the entire cohort are shown in **Table 1**.

BMI data were available for 121 patients. Fifty five percent of the patients had a BMI ≥ 25. Median BMI was 20.9, 26.6 and 34.6 in groups BMI < 25, 25 ≤ BMI < 30 and BMI ≥ 30 respectively. At the time ICI treatment was initiated, metastatic sites were found as follows: lymph nodes 66.7% (n=90), lung 35.6% (n=48), bones 31.9% (n=43) and liver 27.4% (n=37).

The relationship between BMI and ICI response and survival outcomes

We observed differences in ORR according to the BMI category of the patient. The ORR was 45.4% in the BMI ≥ 25 group, versus 16.3% in the BMI < 25 group (P = 0.020) (**Figure 1a**) In the BMI ≥ 25 and BMI < 25 groups, we observed a higher proportion of complete responses to ICI (8 CR vs 1, 22 PR vs 8 and 7 stable disease (SD) vs 10 (**Supplemental table**). Patients with BMI ≥ 30 experienced the longest median OS (24.8 months) compared with those with 25 ≤ BMI < 30 (14.4 months) and BMI < 25 (8.5 months) (P = 0.012). (**Figure 1b**). BMI≥ 30 remained an independent prognostic variable in multivariable analysis (HR = 0.40; 95% CI 0.17-0.96; P=0.040) (**Table 2**).

Metastatic sites' response and survival outcomes

For peer review only - http://bmjopen.bmj.com/site/about/guidelines.xhtml

The overall response rate (ORR) for the entire cohort was 32.2%. The ORR was significantly lower in patients with bone metastasis 16% vs 41% (P = 0.006) (**Figure 2a**), or liver metastasis 16% vs 39% (P = 0.013) (**Figure 2b**). Conversely, the presence of metastatic disease involving the lymph nodes was significantly correlated with higher ORR 40% vs 20% (P = 0.032) (**Figure 2c**). The difference in ORR for lung metastasis was not statistically significant 27% vs 36% (P = 0.340) (**Figure 2d**). Detailed responses are listed in **supplemental table**. The median OS was evaluated for the entire cohort, for each metastatic site and for each BMI group. In the entire cohort, median OS was 12.7 months. Bone, liver and lung metastasis correlated with significantly shorter survival. The median OS for patients with bone metastasis was 7.3 months vs 18 months in the absence of bone metastasis (P < 0.001) (**Figure 3a**). Patients with liver metastasis had a median OS of 8.6 months compared to 15 months (P = 0.006) (**Figure 3b**), and 8.7 months compared to 17.3 months for those with lung metastasis (P = 0.004) (**Figure 3c**). The presence of lymph node metastases was not significantly associated with OS: 13.5 months vs 12.7 months (P = 0.175) (**Figure 3d**). Although bone, lung, and liver metastasis were all associated with significantly worse survival in univariable analyses, bone metastasis was the only metastatic site that remained significantly associated with shorter overall survival in multi-variable analysis (HR = 1.98; 95%CI 1.17-3.35; P = 0.010) (**Table 2**).

NLR response and survival outcomes

Next, we evaluated the relationship between the neutrophil-to-lymphocyte ratio (NLR) and clinical outcomes with ICI in this cohort. The ORR was not statistically different for patients with NLR ≥ 4 compared to those with NLR < 4, respectively 24.5% and 36.9% (P = 0.141) (**Supplemental Figure 1**). However, NLR < 4 was correlated with better OS, with a median of 17.7 months versus 8.2 months for NLR ≥ 4 (P < 0.001) (**Supplemental Figure 2**). The survival benefit associated with a low NLR at the time of initiating ICI therapy was independent of other prognostic variable in multivariable analysis (**Table 2**). We assessed whether there

For peer review only - http://bmjopen.bmj.com/site/about/guidelines.xhtml

was a relationship between NLR and BMI, but we did not observe any significant correlation (data not shown).

Discussion

ICI are approved for a wide-array of cancers based on tumor type (melanoma, NSCLC, urothelial cancer) and/or in a tumor type agnostic manner based on the presence of molecular biomarkers such as high tumor mutation burden (TMB) [29-31] and microsatellite instability [31-33]. However, in most cases, many patients who are eligible for immunotherapies based on tumor type or biomarker status will either not respond to treatment or will eventually become resistant to treatment. In this analysis, we identified several important clinical biomarkers that associate with better (high BMI) or worse (bone metastases, high NLR) outcomes with ICI in patients with mUC. These biomarkers are easily assessed without any additional costly molecular testing. Moreover, ICIs are expensive, potentially toxic, and in mUC only 30% of the patients benefit from ICIs. As such, we believe that these data may be clinically useful to guide treatment decisions.

According to the World Health Organization (WHO) obesity, classified as a body mass index (BMI) greater than 30 kg/m^2, is a rising epidemic [34]. Our data showed strong correlation between elevated BMI, and improved outcome in terms of response as well as survival. To the best of our knowledge, the present study is the largest to report data of ICI outcomes correlation with BMI in mUC [20, 35, 36]. A favorable prognostic role of high BMI was also reported in RCC, NSCLC and melanoma [18, 35, 37-39]. A pooled post hoc analysis of individual participant data from 4 prospective trials with Atezolizumab in metastatic NSCLC showed significant difference in survival between normal weight, overweight and obese patients with improved OS for patients with obesity, and overweight compared to normal weight. [18] In a retrospective multicohort analysis of 1918 metastatic melanoma patients treated with

For peer review only - http://bmjopen.bmj.com/site/about/guidelines.xhtml

chemotherapy, targeted therapies and ICI, McQuade et al studied association between BMI and outcome, as well as interactions between BMI and gender, and type of treatment. Obesity was associated with improved OS and PFS with a benefit restricted to patients treated with targeted therapies and ICI [17].

There is increasing evidence that excess body weight is a modifiable risk factor for several malignancies, including pancreas, kidney, colorectal, postmenopausal breast, ovarian, gallbladder and thyroid cancers [40]. However, obesity is not an established risk factor for bladder cancer, and the impact of obesity on cancer survival is less clear. Some studies have reported that obesity is associated with improved survival in some, but not all, patients with cancer, which is referred to the "obesity paradox" [41]. Numerous studies have shown that obesity can cause immune cell dysfunction [42-45], while others argue that the chronic low-grade inflammation underpinning obesity creates a pro-inflammatory state that synergizes with immunotherapy [46, 47].

Pre-clinical and translational research has provided insight into how immune checkpoint blockade can benefit obese patients. Obesity appears to influence T cell function and phenotype. For example, leptin-associated T cell dysfunction due to obesity has been observed across different species and tumor models, and was found to enhance the immune response to anti-PD-1 therapy [42]. Another mouse study reported that obesity caused a preferential increase and accumulation of senescent $CD44^{hi}CD62L^{lo}CD4+$ T cells that constitutively express PD-1 and CD153 [48]. Moreover, a study in a colon carcinoma mouse model showed that obesity impaired the infiltration, cytokine production and metabolic activity of tumor-infiltrating CD8 T cells, while anti-PD-1 restored CD8 T cells infiltration, proinflammatory cytokine production and metabolic activity, leading to complete tumor eradication and immune memory [49]. Obesity may also affect other immune cells in the TME, such as myeloid-derived suppressor cells (MDSCs), which are known to suppress T cell responses. MDSCs can suppress

For peer review only - http://bmjopen.bmj.com/site/about/guidelines.xhtml

T cell activity through many mechanisms, one of which is expressing PD-L1 to induce T cell exhaustion [50, 51]. In a pre-clinical study, inhibiting PD-L1 on MDSCs decreased their ability to suppress T cell activity, suggesting that obesity could also accelerate tumor progression by promoting the expression of PD-L1 on MDSCs [52]. Finally, a study in a breast cancer model showed that although obesity accelerated tumor progression, anti-PD-1 treatment significantly reduced tumor burden by reshaping the local TME landscape [53].

The relationship between obesity and ICI is complex and depends on various of factors, including the types of cancer and ICI involved. However, BMI may be an imperfect marker and its evaluation based only on weight prior to ICI start can be limiting. Assessment of the weight loss or change over the time may be a better indicator of disease prognosis. As BMI cannot distinguish body fat from muscle, it may not be the best tool to assess obesity. Other measurement methods could be used for better assessment such as dual energy x-ray absorptiometry or fat referenced quantitative MRI [54].

Further studies are needed to understand the molecular mechanisms underlying the interaction between obesity and cancer immunity, and to identify potential targets for effective interventions.

Bone metastasis are a validated negative prognostic factor in mUC treated with platinum based chemotherapy [55, 56]. In our study, poor response and survival outcome of bone and liver metastasis was significant, while lymph nodes metastasis correlated with higher ORR. These results are consistent with published data in the literature for patients treated with ICI [57-59]. Makrakis et al. showed lower response rates and shorter OS for bone and liver metastasis on retrospective data from 917 mUC treated with ICI as 1st or ≥ 2nd line, but higher ORR for lymph node confined metastasis [57]. A retrospective multicentric Japanese study reported shorter ORR for bone metastasis in a cohort of patients treated with Pembrolizumab for mUC [58].

For peer review only

Similar results have also been reported from prospective trials [60, 61]. In the IMvigor 210 phase 2 trial, the ORR on Atezolizumab was 32% for lymph nodes confined metastasis, and only 8% for liver metastasis, data for bone metastasis were not reported [60]. Higher ORR for lymph nodes metastasis (47%) compared to liver metastasis (23%) was also reported in the Keynote 052 trial [61]. Prognostic models for mUC patients treated with ICI identified liver or visceral metastases as poor prognostic factors [62, 63]. However, most prospective trials of ICI in mUC do not use presence/absence of bone metastases as a stratification factor, despite the fact that bone metastases are associated with poor response to chemotherapy, and impact quality of life [55, 56, 64]. Owari et al validated a specific prognostic scoring system, B-FOM, to predict survival for patients with bone metastasis from different genito-urinary cancers, based on 5 prognostic factors: primary tumor (prostate, renal or urothelial cancers), poor performance status, visceral metastasis, high Glasgow-prognostic score and elevated neutrophil-to-lymphocyte ratio [65]. This prediction tool may be helpful to individualize optimal treatment strategy for the patients. A better understanding of the bone microenvironment is crucial to help the development of novel therapeutic strategies and improve outcomes [66].

The distant organ microenvironment, also known as metastatic microenvironment (MME), plays an important role in site-specific metastasis [67]. For instance, macrophages, which are abundant immune cells in the lungs, can bind to cancer cells via receptor VCAM-1 transmit, signaling a chain of events leading to lung-specific metastasis in breast cancer [68]. It has been demonstrated that myeloid cells can remodel the pre-metastatic lung from an immune protective state to a state favoring tumor progression, thereby promoting lung metastasis [69]. Furthermore, lung stromal cells can also promote tumor colonization and metastasis by secreting periostin [70]. In the liver, there is a significant presence of circulating and resident NK cells, which serve as the primary effectors of liver immune function. This NK cell

For peer review only - http://bmjopen.bmj.com/site/about/guidelines.xhtml

maintained the breast cancer cells dormant in the liver by secreting IFNγ, while sustaining NK cell abundance with IL15-based immunotherapy succeeded to prevent liver metastasis and prolong the survival in preclinical models [71]. Conversely, bone forms an immunosuppressive environment mainly due to immature NK cells and a small number of cytotoxic T cells, and a large number of myeloid progenitors and Treg [72]. In a human bladder tumor xenografts model, researchers observed a high infiltration of bone marrow-derived host CD11b myeloid cells. They further demonstrated that enhanced tumor-associated in the TME promote an immunosuppressive pro-tumoral myeloid phenotype [73]. These observations demonstrate how the MME can influence the metastatic potential and outcome of different types of cancer cells in different organs, suggesting that MME might also potentiate site-specific metastasis in bladder cancer. Taken together, all these studies suggest the importance of understanding the metastatic potential and outcome of different types of cancer cells in different organs, through which we can further develop more effective therapeutic measures against bladder cancer.

NLR is a well-established poor prognostic factor in several cancers, independently of treatment type [26-28]. It could be considered as a surrogate marker of chronic inflammation and evasion of immune surveillance [74]. In our study, high NLR was associated with shorter median OS. A recent study by Valero et al showed poor response and survival outcomes, for 1917 patients with high NLR at diagnosis, treated with ICI for multiple cancer types [74]. Similar results were reported for melanoma and NSCLC [27, 75]. Banna et al explored the prognostic and predictive role of NLR and LDH in metastatic urothelial cancers treated with ICI, and showed significant correlation with PFS and OS for high NLR [76]. For patients treated with ICI for mUC, a high NLR prior to first line chemotherapy and second line pembrolizumab was associated with worse survival outcomes [77]. NLR may represent an accessible low-cost predictive biomarker.

For peer review only - http://bmjopen.bmj.com/site/about/guidelines.xhtml

The present study has some strengths and limitations. It presents a relatively large multi-center cohort of patients with significant prognostic factors. On the other hand, this is a retrospective study, with heterogenous population, and an investigator-based response assessment on clinical and radiological chart review. However, these observations made in this analysis are hypothesis generating. The evaluation of obesity and its relationship with ICI response warrants future prospective studies that incorporate better established measures of obesity and metabolic syndrome. Moreover, little is known about how bladder cancer cells interact with the liver and bone tumor immune microenvironments and this warrants further investigation in preclinical models to develop optimal immunotherapies for patients with bone and liver mUC.

Conclusion

Our study identified elevated BMI, NLR and presence of bone metastases as potential biomarkers for ICI response and survival in mUC. Obesity was associated with improved survival and response rate, whereas bone metastases and high NLR are associated with lack of response and shorter survival. Prospective validation of these data is warranted, especially in the evolving landscape of therapeutic options for mUC with novel agents and combinations with ICI [78].

For peer review only

REFERENCES

1. Bray, F., et al., *Global cancer statistics 2018: GLOBOCAN estimates of incidence and mortality worldwide for 36 cancers in 185 countries.* CA: A Cancer Journal for Clinicians, 2018. **68**(6): p. 394-424.
2. Burger, M., et al., *Epidemiology and risk factors of urothelial bladder cancer.* Eur Urol, 2013. **63**(2): p. 234-41.
3. Humphrey, P.A., et al., *The 2016 WHO Classification of Tumours of the Urinary System and Male Genital Organs—Part B: Prostate and Bladder Tumours.* European Urology, 2016. **70**(1): p. 106-119.
4. Nadal, R. and J. Bellmunt, *Management of metastatic bladder cancer.* Cancer Treatment Reviews, 2019. **76**: p. 10-21.
5. Witjes, J.A., et al., *European Association of Urology Guidelines on Muscle-invasive and Metastatic Bladder Cancer: Summary of the 2020 Guidelines.* Eur Urol, 2021. **79**(1): p. 82-104.
6. Powles, T., et al., *Avelumab Maintenance Therapy for Advanced or Metastatic Urothelial Carcinoma.* N Engl J Med, 2020. **383**(13): p. 1218-1230.
7. Bellmunt, J., et al., *Pembrolizumab as Second-Line Therapy for Advanced Urothelial Carcinoma.* N Engl J Med, 2017. **376**(11): p. 1015-1026.
8. McCaffrey, J.A., et al., *Phase II trial of docetaxel in patients with advanced or metastatic transitional-cell carcinoma.* J Clin Oncol, 1997. **15**(5): p. 1853-7.
9. Dreicer, R., et al., *Paclitaxel in advanced urothelial carcinoma: its role in patients with renal insufficiency and as salvage therapy.* J Urol, 1996. **156**(5): p. 1606-8.
10. Ko, Y.J., et al., *Nanoparticle albumin-bound paclitaxel for second-line treatment of metastatic urothelial carcinoma: a single group, multicentre, phase 2 study.* Lancet Oncol, 2013. **14**(8): p. 769-76.
11. Bellmunt, J., et al., *Long-term survival results of a randomized phase III trial of vinflunine plus best supportive care versus best supportive care alone in advanced urothelial carcinoma patients after failure of platinum-based chemotherapy.* Ann Oncol, 2013. **24**(6): p. 1466-72.
12. Fradet, Y., et al., *Randomized phase III KEYNOTE-045 trial of pembrolizumab versus paclitaxel, docetaxel, or vinflunine in recurrent advanced urothelial cancer: results of >2 years of follow-up.* Ann Oncol, 2019. **30**(6): p. 970-976.
13. Rosenberg, J.E., et al., *Atezolizumab in patients with locally advanced and metastatic urothelial carcinoma who have progressed following treatment with platinum-based chemotherapy: a single-arm, multicentre, phase 2 trial.* Lancet, 2016. **387**(10031): p. 1909-20.
14. Powles, T., et al., *Atezolizumab versus chemotherapy in patients with platinum-treated locally advanced or metastatic urothelial carcinoma (IMvigor211): a multicentre, open-label, phase 3 randomised controlled trial.* Lancet, 2018. **391**(10122): p. 748-757.
15. Sharma, P., et al., *Nivolumab in metastatic urothelial carcinoma after platinum therapy (CheckMate 275): a multicentre, single-arm, phase 2 trial.* Lancet Oncol, 2017. **18**(3): p. 312-322.
16. Sharma, P., et al., *Nivolumab Alone and With Ipilimumab in Previously Treated Metastatic Urothelial Carcinoma: CheckMate 032 Nivolumab 1 mg/kg Plus Ipilimumab 3 mg/kg Expansion Cohort Results.* J Clin Oncol, 2019. **37**(19): p. 1608-1616.
17. McQuade, J.L., et al., *Association of body-mass index and outcomes in patients with metastatic melanoma treated with targeted therapy, immunotherapy, or chemotherapy: a retrospective, multicohort analysis.* Lancet Oncol, 2018. **19**(3): p. 310-322.
18. Kichenadasse, G., et al., *Association Between Body Mass Index and Overall Survival With Immune Checkpoint Inhibitor Therapy for Advanced Non-Small Cell Lung Cancer.* JAMA Oncol, 2020. **6**(4): p. 512-518.

For peer review only - http://bmjopen.bmj.com/site/about/guidelines.xhtml

19. Albiges, L., et al., *Body Mass Index and Metastatic Renal Cell Carcinoma: Clinical and Biological Correlations.* J Clin Oncol, 2016. **34**(30): p. 3655-3663.
20. Indini, A., et al., *Impact of BMI on Survival Outcomes of Immunotherapy in Solid Tumors: A Systematic Review.* Int J Mol Sci, 2021. **22**(5).
21. Ganesh, K. and J. Massague, *Targeting metastatic cancer.* Nat Med, 2021. **27**(1): p. 34-44.
22. Oliver, A.J., et al., *Tissue-Dependent Tumor Microenvironments and Their Impact on Immunotherapy Responses.* Front Immunol, 2018. **9**: p. 70.
23. Furubayashi, N., et al., *Organ-Specific Tumor Response to Pembrolizumab in Advanced Urothelial Carcinoma After Platinum-Based Chemotherapy.* Onco Targets Ther, 2021. **14**: p. 1981-1988.
24. Tumeh, P.C., et al., *Liver Metastasis and Treatment Outcome with Anti-PD-1 Monoclonal Antibody in Patients with Melanoma and NSCLC.* Cancer Immunol Res, 2017. **5**(5): p. 417-424.
25. Schmid, S., et al., *Organ-specific response to nivolumab in patients with non-small cell lung cancer (NSCLC).* Cancer Immunol Immunother, 2018. **67**(12): p. 1825-1832.
26. Guthrie, G.J., et al., *The systemic inflammation-based neutrophil-lymphocyte ratio: experience in patients with cancer.* Crit Rev Oncol Hematol, 2013. **88**(1): p. 218-30.
27. Cedres, S., et al., *Neutrophil to lymphocyte ratio (NLR) as an indicator of poor prognosis in stage IV non-small cell lung cancer.* Clin Transl Oncol, 2012. **14**(11): p. 864-9.
28. Templeton, A.J., et al., *Prognostic role of neutrophil-to-lymphocyte ratio in solid tumors: a systematic review and meta-analysis.* J Natl Cancer Inst, 2014. **106**(6): p. dju124.
29. Kim, S.I., C.R. Cassella, and K.T. Byrne, *Tumor Burden and Immunotherapy: Impact on Immune Infiltration and Therapeutic Outcomes.* Front Immunol, 2020. **11**: p. 629722.
30. Samstein, R.M., et al., *Tumor mutational load predicts survival after immunotherapy across multiple cancer types.* Nat Genet, 2019. **51**(2): p. 202-206.
31. Palmeri, M., et al., *Real-world application of tumor mutational burden-high (TMB-high) and microsatellite instability (MSI) confirms their utility as immunotherapy biomarkers.* ESMO Open, 2022. **7**(1): p. 100336.
32. O'Malley, D.M., et al., *Pembrolizumab in Patients With Microsatellite Instability-High Advanced Endometrial Cancer: Results From the KEYNOTE-158 Study.* J Clin Oncol, 2022. **40**(7): p. 752-761.
33. Maio, M., et al., *Pembrolizumab in microsatellite instability high or mismatch repair deficient cancers: updated analysis from the phase II KEYNOTE-158 study.* Ann Oncol, 2022. **33**(9): p. 929-938.
34. Jensen, M.D., et al., *2013 AHA/ACC/TOS guideline for the management of overweight and obesity in adults: a report of the American College of Cardiology/American Heart Association Task Force on Practice Guidelines and The Obesity Society.* Circulation, 2014. **129**(25 Suppl 2): p. S102-38.
35. Cortellini, A., et al., *A multicenter study of body mass index in cancer patients treated with anti-PD-1/PD-L1 immune checkpoint inhibitors: when overweight becomes favorable.* Journal for ImmunoTherapy of Cancer, 2019. **7**(1).
36. Yoo, S.K., et al., *Outcomes Among Patients With or Without Obesity and With Cancer Following Treatment With Immune Checkpoint Blockade.* JAMA Netw Open, 2022. **5**(2): p. e220448.
37. Martini, D.J., et al., *Adiposity may predict survival in patients with advanced stage cancer treated with immunotherapy in phase 1 clinical trials.* Cancer, 2020. **126**(3): p. 575-582.
38. Rutkowski, P., et al., *Body mass index (BMI) and outcome of metastatic melanoma patients receiving targeted therapy and immunotherapy: a multicenter international retrospective study.* Journal for ImmunoTherapy of Cancer, 2020. **8**(2): p. e001117.
39. Richtig, G., et al., *Body mass index may predict the response to ipilimumab in metastatic melanoma: An observational multi-centre study.* PLOS ONE, 2018. **13**(10): p. e0204729.

For peer review only - http://bmjopen.bmj.com/site/about/guidelines.xhtml

40. Lauby-Secretan, B., et al., *Body fatness and cancer—viewpoint of the IARC Working Group.* New England journal of medicine, 2016. **375**(8): p. 794-798.
41. Lennon, H., et al., *The obesity paradox in cancer: a review.* Current oncology reports, 2016. **18**: p. 1-8.
42. Wang, Z., et al., *Paradoxical effects of obesity on T cell function during tumor progression and PD-1 checkpoint blockade.* Nature medicine, 2019. **25**(1): p. 141-151.
43. Rebeles, J., et al., *Obesity-induced changes in T-cell metabolism are associated with impaired memory T-cell response to influenza and are not reversed with weight loss.* The Journal of Infectious Diseases, 2019. **219**(10): p. 1652-1661.
44. Gibson, J.T., et al., *Obesity-associated myeloid-derived suppressor cells promote apoptosis of tumor-infiltrating CD8 T cells and immunotherapy resistance in breast cancer.* Frontiers in immunology, 2020. **11**: p. 590794.
45. Ringel, A.E., et al., *Obesity shapes metabolism in the tumor microenvironment to suppress anti-tumor immunity.* Cell, 2020. **183**(7): p. 1848-1866. e26.
46. Wang, Z., A.M. Monjazeb, and W.J. Murphy, *The complicated effects of obesity on cancer and immunotherapy*. 2019, Future Medicine. p. 11-14.
47. Fang, H., et al., *Regulating the Obesity-Related Tumor Microenvironment to Improve Cancer Immunotherapy.* ACS nano, 2023. **17**(5): p. 4748-4763.
48. Shirakawa, K., et al., *Obesity accelerates T cell senescence in murine visceral adipose tissue.* The Journal of clinical investigation, 2016. **126**(12): p. 4626-4639.
49. Dyck, L., et al., *Suppressive effects of the obese tumor microenvironment on CD8 T cell infiltration and effector function.* Journal of Experimental Medicine, 2022. **219**(3).
50. Lau, J., et al., *Tumour and host cell PD-L1 is required to mediate suppression of anti-tumour immunity in mice.* Nature communications, 2017. **8**(1): p. 14572.
51. Weber, R., et al., *Myeloid-derived suppressor cells hinder the anti-cancer activity of immune checkpoint inhibitors.* Frontiers in immunology, 2018. **9**: p. 1310.
52. Hale, M., et al., *Obesity triggers enhanced MDSC accumulation in murine renal tumors via elevated local production of CCL2.* PloS one, 2015. **10**(3): p. e0118784.
53. Pingili, A.K., et al., *Immune checkpoint blockade reprograms systemic immune landscape and tumor microenvironment in obesity-associated breast cancer.* Cell reports, 2021. **35**(12): p. 109285.
54. Borga, M., et al., *Advanced body composition assessment: from body mass index to body composition profiling.* J Investig Med, 2018. **66**(5): p. 1-9.
55. Alqaisi, H.A., et al., *The prognostic impact of bone metastasis in patients with metastatic urothelial carcinoma treated with first-line platinum-based chemotherapy.* Ther Adv Med Oncol, 2022. **14**: p. 17588359221094879.
56. Necchi, A., et al., *Nomogram-based Prediction of Overall Survival in Patients with Metastatic Urothelial Carcinoma Receiving First-line Platinum-based Chemotherapy: Retrospective International Study of Invasive/Advanced Cancer of the Urothelium (RISC).* Eur Urol, 2017. **71**(2): p. 281-289.
57. Makrakis, D., et al., *Association Between Sites of Metastasis and Outcomes With Immune Checkpoint Inhibitors in Advanced Urothelial Carcinoma.* Clin Genitourin Cancer, 2022. **20**(5): p. e440-e452.
58. Shimizu, T., et al., *Organ-Specific and Mixed Responses to Pembrolizumab in Patients with Unresectable or Metastatic Urothelial Carcinoma: A Multicenter Retrospective Study.* Cancers (Basel), 2022. **14**(7).
59. Raggi, D., et al., *Role of Bone Metastases in Patients Receiving Immunotherapy for Pre-Treated Urothelial Carcinoma: The Multicentre, Retrospective Meet-URO-1 Bone Study.* Clin Genitourin Cancer, 2022. **20**(2): p. 155-164.

For peer review only - http://bmjopen.bmj.com/site/about/guidelines.xhtml

60. Balar, A.V., et al., *Atezolizumab as first-line treatment in cisplatin-ineligible patients with locally advanced and metastatic urothelial carcinoma: a single-arm, multicentre, phase 2 trial.* Lancet, 2017. **389**(10064): p. 67-76.
61. Balar, A.V., et al., *First-line pembrolizumab in cisplatin-ineligible patients with locally advanced and unresectable or metastatic urothelial cancer (KEYNOTE-052): a multicentre, single-arm, phase 2 study.* Lancet Oncol, 2017. **18**(11): p. 1483-1492.
62. Nassar, A.H., et al., *A model combining clinical and genomic factors to predict response to PD-1/PD-L1 blockade in advanced urothelial carcinoma.* Br J Cancer, 2020. **122**(4): p. 555-563.
63. Khaki, A.R., et al., *A New Prognostic Model in Patients with Advanced Urothelial Carcinoma Treated with First-line Immune Checkpoint Inhibitors.* Eur Urol Oncol, 2021. **4**(3): p. 464-472.
64. Stellato, M., et al., *Bone metastases from urothelial carcinoma. The dark side of the moon.* J Bone Oncol, 2021. **31**: p. 100405.
65. Owari, T., et al., *External validation of a genitourinary cancer-specific prognostic scoring system to predict survival for patients with bone metastasis (modified B-FOM scoring model): Comparison with other scoring models in terms of accuracy.* J Bone Oncol, 2021. **26**: p. 100344.
66. Reinstein, Z.Z., et al., *Overcoming immunosuppression in bone metastases.* Crit Rev Oncol Hematol, 2017. **117**: p. 114-127.
67. Liu, Q., et al., *Factors involved in cancer metastasis: a better understanding to "seed and soil" hypothesis.* Molecular Cancer, 2017. **16**(1): p. 176.
68. Chen, Q., X.H.-F. Zhang, and J. Massagué, *Macrophage binding to receptor VCAM-1 transmits survival signals in breast cancer cells that invade the lungs.* Cancer cell, 2011. **20**(4): p. 538-549.
69. Yan, H.H., et al., *Gr-1+ CD11b+ myeloid cells tip the balance of immune protection to tumor promotion in the premetastatic lung.* Cancer research, 2010. **70**(15): p. 6139-6149.
70. Malanchi, I., et al., *Interactions between cancer stem cells and their niche govern metastatic colonization.* Nature, 2012. **481**(7379): p. 85-89.
71. Correia, A.L., et al., *Hepatic stellate cells suppress NK cell-sustained breast cancer dormancy.* Nature, 2021. **594**(7864): p. 566-571.
72. Zhao, E., et al., *Bone marrow and the control of immunity.* Cellular & molecular immunology, 2012. **9**(1): p. 11-19.
73. Eruslanov, E., et al., *Aberrant PGE2 metabolism in bladder tumor microenvironment promotes immunosuppressive phenotype of tumor-infiltrating myeloid cells.* International immunopharmacology, 2011. **11**(7): p. 848-855.
74. Valero, C., et al., *Pretreatment neutrophil-to-lymphocyte ratio and mutational burden as biomarkers of tumor response to immune checkpoint inhibitors.* Nat Commun, 2021. **12**(1): p. 729.
75. Capone, M., et al., *Baseline neutrophil-to-lymphocyte ratio (NLR) and derived NLR could predict overall survival in patients with advanced melanoma treated with nivolumab.* J Immunother Cancer, 2018. **6**(1): p. 74.
76. Banna, G.L., et al., *Neutrophil-to-lymphocyte ratio and lactate dehydrogenase as biomarkers for urothelial cancer treated with immunotherapy.* Clin Transl Oncol, 2020. **22**(11): p. 2130-2135.
77. Kobayashi, T., et al., *Pre-pembrolizumab neutrophil-to-lymphocyte ratio (NLR) predicts the efficacy of second-line pembrolizumab treatment in urothelial cancer regardless of the pre-chemo NLR.* Cancer Immunol Immunother, 2022. **71**(2): p. 461-471.
78. Hoimes, C.J., et al., *Enfortumab Vedotin Plus Pembrolizumab in Previously Untreated Advanced Urothelial Cancer.* J Clin Oncol, 2023. **41**(1): p. 22-31.

For peer review only

For peer review only - http://bmjopen.bmj.com/site/about/guidelines.xhtml

	N (%)
Age	
Age ≤ 60 years	28 (20.7%)
Age > 60 years	107 (79.3%)
Gender	
Male	101 (74.8%)
Female	34 (25.2%)
Ever smoker	69 (51.9%)
Primary	
Bladder	125 (92.5%)
Upper tract	10 (7.4%)
Pathology	
Urothelial	123 (91.1%)
Squamous	7 (5.1%)
Other	5 (3.7%)
ECOG	
0-1	112 (83%)
≥ 2	15 (11.1%)
Missing data	8 (5.9%)
Metastatic sites	
LN	90 (66.7%)
Lung	48 (35.6%)
Bone	43 (31.9%)
Liver	37 (27.4%)
BMI n=121	
< 25	55 (45.4%)
25-30	43 (35.5%)
≥ 30	23 (19%)
ICI Line	
First	51 (37.8%)
Second or later	84 (62.2%)

Table 1. Patients' characteristics

For peer review only

For peer review only - http://bmjopen.bmj.com/site/about/guidelines.xhtml

OS	Univariable			Multivariable		
	HR	95% CI	p value	HR	95% CI	p value
BMI ≥ 30 vs < 30	0.63	0.45-0.88	0.012	0.40	0.17-0.96	0.040
Male vs Female	0.95	0.58-1.57	0.867			
Age ≥ 60 vs < 60	1.88	0.65-5.45	0.235			
ECOG ≥ 2 vs 0-1	1.68	0.86-3.29	0.125	2.21	1.02-4.78	0.042
ICI Line 1 vs ≥ 2	2.39	1.43-3.97	0.001	1.80	1.31-2.48	0.000
NLR < 4 vs ≥ 4	2.46	1.53-3.94	0.000	2.66	1.58-4.49	0.000
Bone metastasis Yes vs No	2.38	1.52-3.73	0.000	1.98	1.17-3.35	0.010
Lung metastasis Yes vs No	1.88	1.21-2.93	0.005			
Liver metastasis Yes vs No	1.92	1.2-3.09	0.006			

Table 2. Uni- and Multivariable analysis for OS

For peer review only

For peer review only - http://bmjopen.bmj.com/site/about/guidelines.xhtml

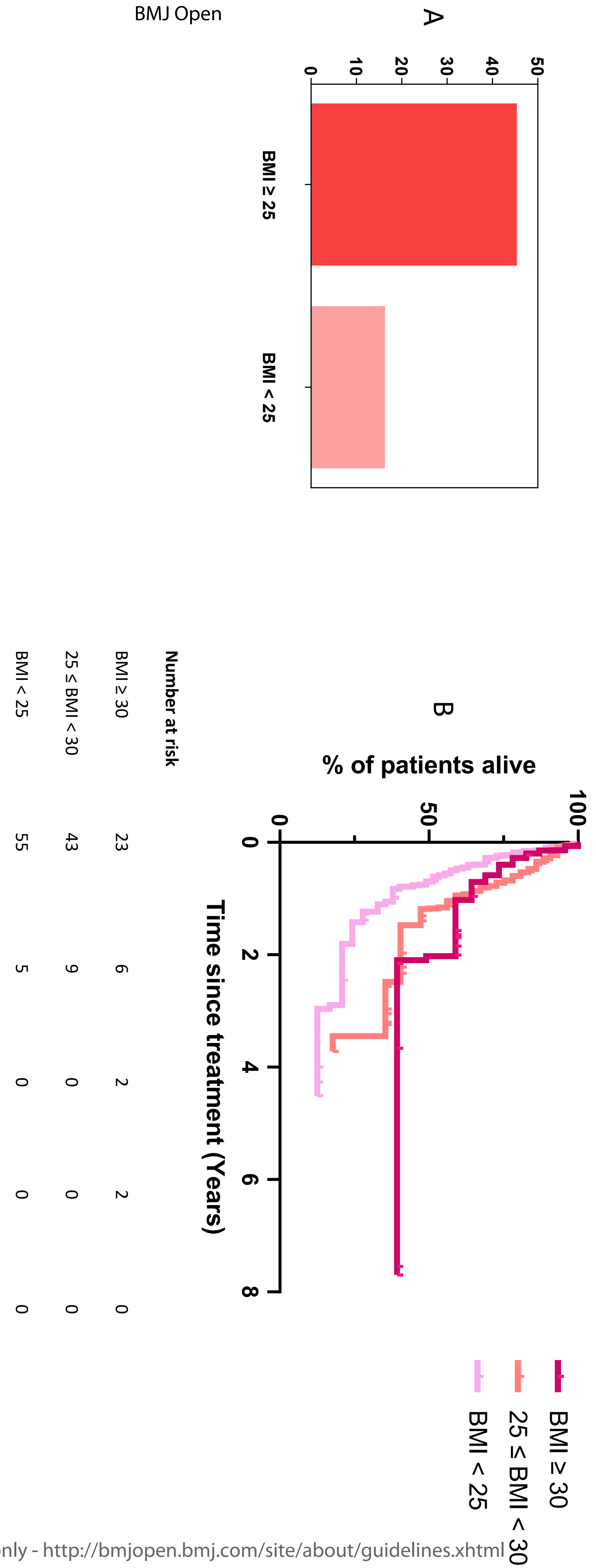

Figure 1. Overall response rate and overall survival according to BMI groups

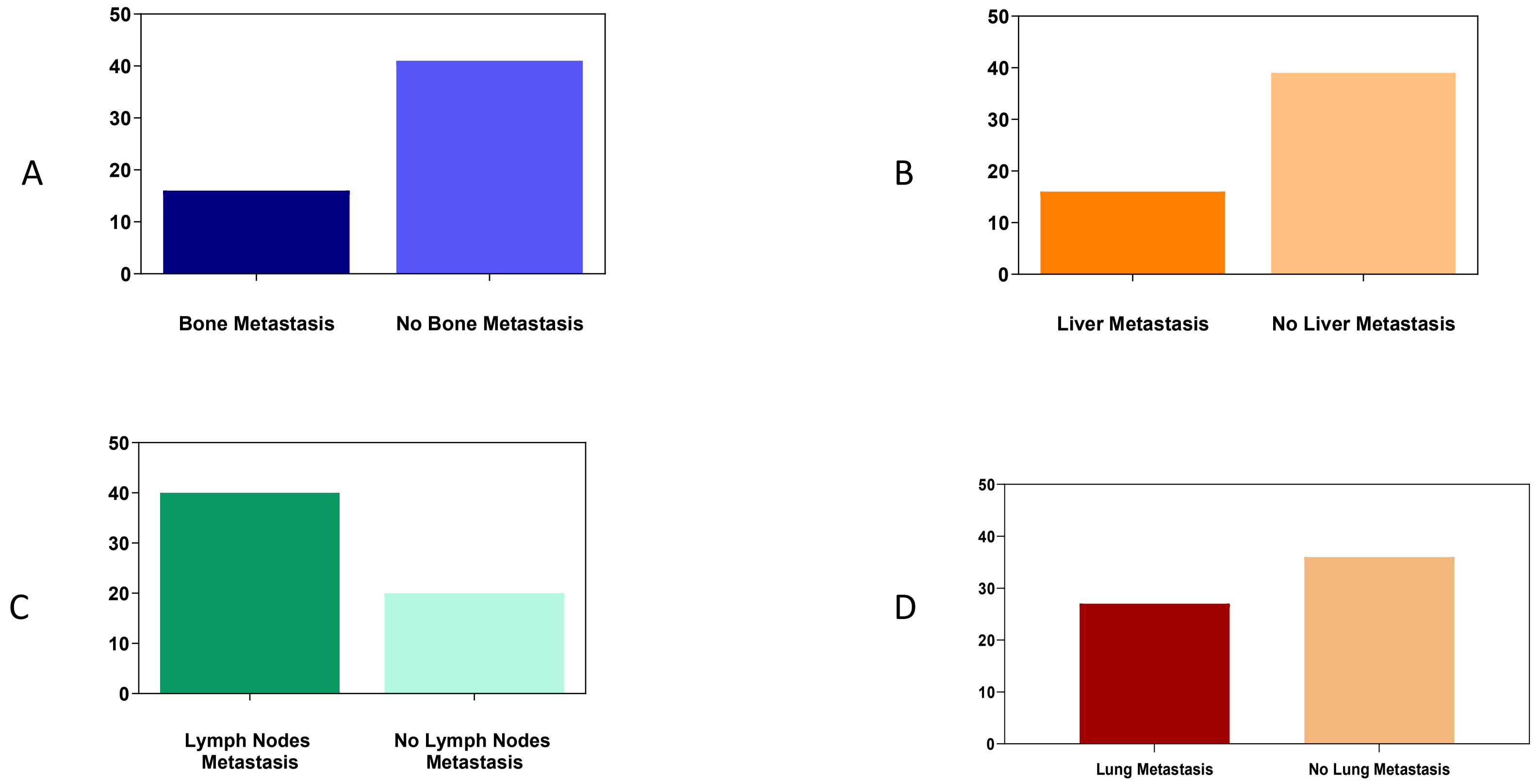

Figure 2. Overall response rate according to metastatic sites

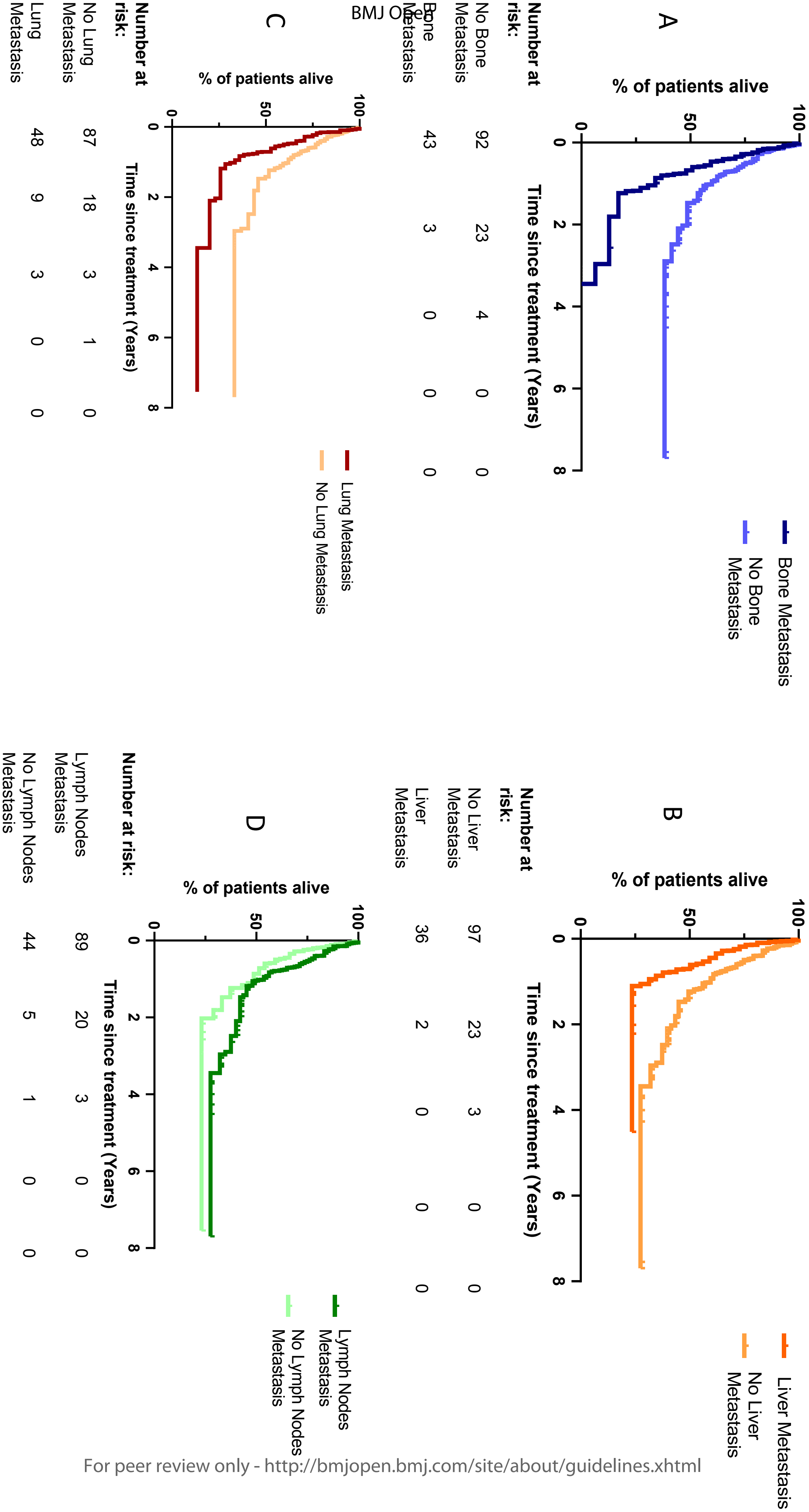

Figure 3 Overall survival according to metastatic sites

	BMI ≥ 25	BMI < 25	Lymphnode	Lung	Bone	Liver
ORR (CR+PR), %	45.4	16.3	40	27	16	16
CR, n (%)	8 (12.1)	1 (1.8)	7 (7.77%)	2 (4.16%)	1 (2.1%)	1 (2.5%)
PR, n (%)	22 (33.3)	8 (14.5)	29 (32.2%)	11 (22.9%)	6 (13.9%)	5 (13.5%)
SD, n (%)	7 (10.6)	10 (18.1)	10 (11.1%)	3 (6.25%)	6 (13.9%)	3 (8.1%)
PD, n (%)	29 (43.9)	36 (65.4)	44 (48.8%)	32 (66.6%)	30 (69.7%)	28 (75.6%)

Supplemental Table. Responses according to clinical variables of BMI and metastatic sites

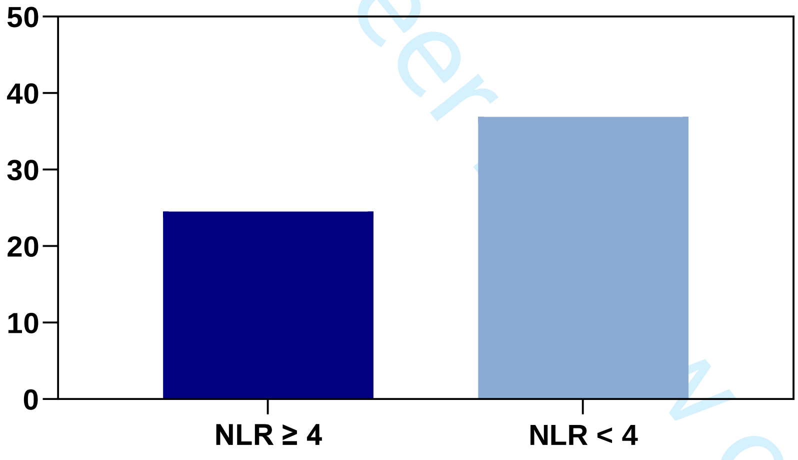

Supplemental Figure 1 Overall response rate according to NLR

For peer review only

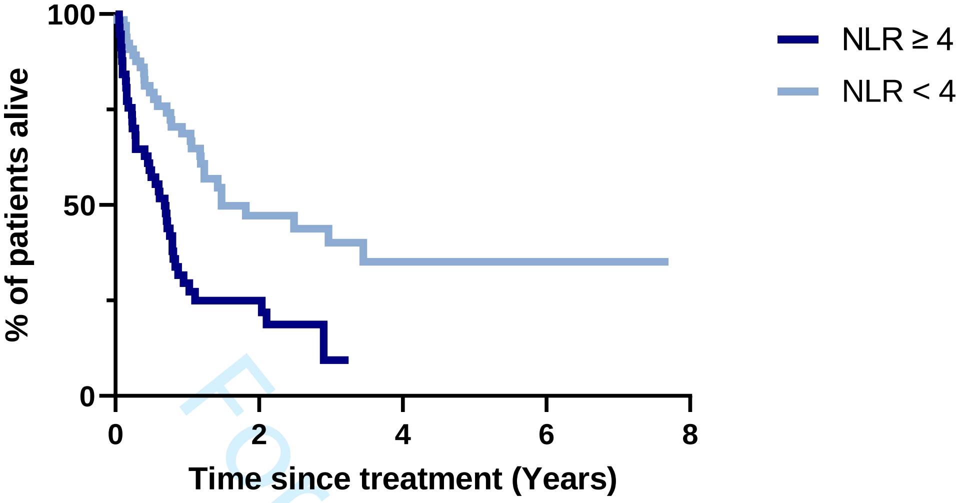

Number at risk:

NLR < 4	65	16	5	2	0
NLR ≥ 4	57	8	0	0	0

Supplemental Figure 2 Overall response rate according to NLR

For peer review only

STROBE Statement—Checklist of items that should be included in reports of ***cohort studies***

		Item No	Recommendation	
**Title and abstract**	✓	1	(*a*) Indicate the study's design with a commonly used term in the title or the abstract	
			(*b*) Provide in the abstract an informative and balanced summary of what was done and what was found	
**Introduction**				Abstract - Page 2
Background/rationale	✓	2	Explain the scientific background and rationale for the investigation being reported	
Objectives	✓	3	State specific objectives, including any prespecified hypotheses	
**Methods**				Page 3-4
Study design	✓	4	Present key elements of study design early in the paper	Page 5
Setting	✓	5	Describe the setting, locations, and relevant dates, including periods of recruitment, exposure, follow-up, and data collection	Page 5
Participants	✓	6	(*a*) Give the eligibility criteria, and the sources and methods of selection of participants. Describe methods of follow-up	Page 5
			(*b*) For matched studies, give matching criteria and number of exposed and unexposed	N/A
Variables	✓	7	Clearly define all outcomes, exposures, predictors, potential confounders, and effect modifiers. Give diagnostic criteria, if applicable	Page 5
Data sources/ measurement	✓	8*	For each variable of interest, give sources of data and details of methods of assessment (measurement). Describe comparability of assessment methods if there is more than one group	Page 5
Bias	✓	9	Describe any efforts to address potential sources of bias	Page 13
Study size	✓	10	Explain how the study size was arrived at	Page 5
Quantitative variables	✓	11	Explain how quantitative variables were handled in the analyses. If applicable, describe which groupings were chosen and why	Page 5
Statistical methods	✓	12	(*a*) Describe all statistical methods, including those used to control for confounding	
			(*b*) Describe any methods used to examine subgroups and interactions	Page 5 & 6
			(*c*) Explain how missing data were addressed	
			(*d*) If applicable, explain how loss to follow-up was addressed	
			(*e*) Describe any sensitivity analyses	
**Results**				
Participants	✓	13*	(a) Report numbers of individuals at each stage of study—eg numbers potentially eligible, examined for eligibility, confirmed eligible, included in the study, completing follow-up, and analysed	Page 5
			(b) Give reasons for non-participation at each stage	
			(c) Consider use of a flow diagram	
Descriptive data	✓	14*	(a) Give characteristics of study participants (eg demographic, clinical, social) and information on exposures and potential confounders	Page 6
			(b) Indicate number of participants with missing data for each variable of interest	Page 18
			(c) Summarise follow-up time (eg, average and total amount)	Page 6
Outcome data	✓	15*	Report numbers of outcome events or summary measures over time	Page 6-7
Main results	✓	16	(*a*) Give unadjusted estimates and, if applicable, confounder-adjusted estimates and their precision (eg, 95% confidence interval). Make clear which confounders were adjusted for and why they were included	Page 19
			(*b*) Report category boundaries when continuous variables were categorized	Page 19
			(*c*) If relevant, consider translating estimates of relative risk into absolute risk for a meaningful time period	N/A

For peer review only

For peer review only - http://bmjopen.bmj.com/site/about/guidelines.xhtml

Other analyses	✓	17	Report other analyses done—eg analyses of subgroups and interactions, and sensitivity analyses Page 6-7
**Discussion**			
Key results	✓	18	Summarise key results with reference to study objectives Page 8-12
Limitations	✓	19	Discuss limitations of the study, taking into account sources of potential bias or imprecision. Discuss both direction and magnitude of any potential bias Page 13
Interpretation	✓	20	Give a cautious overall interpretation of results considering objectives, limitations, multiplicity of analyses, results from similar studies, and other relevant evidence Page 8-13
Generalisability	✓	21	Discuss the generalisability (external validity) of the study results Page 13
**Other information**			
Funding	✓	22	Give the source of funding and the role of the funders for the present study and, if applicable, for the original study on which the present article is based Page 18

*Give information separately for exposed and unexposed groups.

Note: An Explanation and Elaboration article discusses each checklist item and gives methodological background and published examples of transparent reporting. The STROBE checklist is best used in conjunction with this article (freely available on the Web sites of PLoS Medicine at http://www.plosmedicine.org/, Annals of Internal Medicine at http://www.annals.org/, and Epidemiology at http://www.epidem.com/). Information on the STROBE Initiative is available at http://www.strobe-statement.org.

For peer review only

For peer review only - http://bmjopen.bmj.com/site/about/guidelines.xhtml

BMJ Open

Clinical variables associated with immune checkpoint inhibitor outcomes in patients with metastatic urothelial carcinoma: A multicenter retrospective cohort study

Journal:	*BMJ Open*
Manuscript ID	bmjopen-2023-081480.R1
Article Type:	Original research
Date Submitted by the Author:	03-Feb-2024

Complete List of Authors:	Labidi, Soumaya; Jewish General Hospital, Segal Cancer Centre; McGill University<br>Meti, Nicholas; McGill University<br>Barua, Reeta; Toronto East Health Network Michael Garron Hospital<br>Li, Mengqi; Lady Davis Institute for Medical Research; McGill University<br>Riromar, Jamila; The Royal Hospital, National Oncology Center; University Health Network<br>Jiang, Di Maria; University Health Network; University of Toronto<br>Fallah-Rad, Nazanin; University Health Network; University of Toronto<br>Sridhar, Srikala ; Princess Margaret Hospital Cancer Centre, Medical Oncology<br>Del Rincon, Sonia V.; Lady Davis Institute for Medical Research; McGill University, Oncology<br>Pezo, Rossanna C.; Sunnybrook Health Sciences Centre; University of Toronto<br>Ferrario, Cristiano; Jewish General Hospital, Segal Cancer Centre; McGill University<br>Cheng, Susanna; Sunnybrook Health Sciences Centre; University of Toronto<br>Sacher, Adrian; Princess Margaret Hospital Cancer Centre, Medical Oncology<br>Rose, April; Jewish General Hospital, Segal Cancer Centre; McGill University, Oncology

**Primary Subject Heading**:	Oncology
Secondary Subject Heading:	Oncology, Urology
Keywords:	Urological tumours < ONCOLOGY, Adult oncology < ONCOLOGY, Clinical Reasoning, EPIDEMIOLOGY, ONCOLOGY

SCHOLARONE™ Manuscripts

I, the Submitting Author has the right to grant and does grant on behalf of all authors of the Work (as defined in the below author licence), an exclusive licence and/or a non-exclusive licence for contributions from authors who are: i) UK Crown employees; ii) where BMJ has agreed a CC-BY licence shall apply, and/or iii) in accordance with the terms applicable for US Federal Government officers or employees acting as part of their official duties; on a worldwide, perpetual, irrevocable, royalty-free basis to BMJ Publishing Group Ltd ("BMJ") its licensees and where the relevant Journal is co-owned by BMJ to the co-owners of the Journal, to publish the Work in this journal and any other BMJ products and to exploit all rights, as set out in our licence.

The Submitting Author accepts and understands that any supply made under these terms is made by BMJ to the Submitting Author unless you are acting as an employee on behalf of your employer or a postgraduate student of an affiliated institution which is paying any applicable article publishing charge ("APC") for Open Access articles. Where the Submitting Author wishes to make the Work available on an Open Access basis (and intends to pay the relevant APC), the terms of reuse of such Open Access shall be governed by a Creative Commons licence – details of these licences and which Creative Commons licence will apply to this Work are set out in our licence referred to above.

Other than as permitted in any relevant BMJ Author's Self Archiving Policies, I confirm this Work has not been accepted for publication elsewhere, is not being considered for publication elsewhere and does not duplicate material already published. I confirm all authors consent to publication of this Work and authorise the granting of this licence.

For peer review only

Clinical variables associated with immune checkpoint inhibitor outcomes in patients with metastatic urothelial carcinoma: A multicenter retrospective cohort study

Soumaya Labidi[1,2], Nicholas Meti[3], Reeta Barua[4], Mengqi Li[5,6], Jamila Riromar[7], Di Maria Jiang[8], Nazanin Fallah-Rad[8], Srikala S. Sridhar[8], Sonia V. del Rincon[1,5,6], Rossanna C. Pezo[9], Cristiano Ferrario[1,2], Susanna Cheng[9], Adrian G. Sacher[8]*, April A. N. Rose[1,2,5,6]*

1. Gerald Bronfman Department of Oncology, McGill University, Montréal, QC. Canada
2. Segal Cancer Center, Jewish General Hospital. Montréal, QC, Canada
3. St. Mary's Hospital, Montréal, QC. Canada
4. Michael Garron Hospital. Toronto East Health Network. Toronto, ON. Canada
5. Lady Davis Institute, Jewish General Hospital, Montréal, QC, Canada
6. Division of Experimental Medicine, Faculty of Medicine, McGill University, Montréal, QC, Canada
7. Royal Hospital National Oncology Center, Sultanat, Oman
8. Princess Margaret Cancer Center. Toronto, ON, Canada
9. Odette Cancer Center. Sunnybrook Health Sciences Center. Toronto, ON, Canada

*Equal contribution

Corresponding Author:

April Rose, MD, PhD, FRCPC

3755 Chemin de la Côte-Sainte-Catherine

Office E-444

Montreal, QC, Canada H3T 1E2

Phone: (514) 340-8222 xt 27343

Email: april.rose@mcgill.ca

Running head: Immunotherapy outcomes in urothelial cancer

For peer review only

For peer review only - http://bmjopen.bmj.com/site/about/guidelines.xhtml

Abstract

Objectives: Immune checkpoint inhibitors (ICI) are indicated for metastatic urothelial cancer (mUC), but predictive and prognostic factors are lacking. We investigated clinical variables associated with ICI outcomes.

Methods: We performed a multicenter retrospective cohort study of 135 patients who received ICI for mUC, 2016-2021, at 3 Canadian centres. Clinical characteristics, body mass index (BMI), metastatic sites, neutrophil-to-lymphocyte ratio (NLR), response, and survival were abstracted from chart review.

Results: We identified 135 patients and 62% had received ICI as a second line or later treatment for mUC. A BMI ≥ 25 was significantly correlated to a higher overall response rate (ORR) (45.4% vs 16.3%, P = 0.020). Patients with BMI ≥ 30 experienced longer median overall survival (OS) 24.8 vs. 14.4 for 25 ≤ BMI < 30 and 8.5 months for BMI < 25 (P-value = 0.012). The ORR was lower in the presence of bone metastases 16% vs 41% P-value = 0.006, and liver metastases 16% vs 39% P-value = 0.013. Metastatic lymphnodes were correlated with higher ORR 40% vs 20% P = 0.032. The median OS for bone metastases was 7.3 vs 18 months (P-value < 0.001). Patients with liver metastases had a median OS of 8.6 vs 15 months (P-value = 0.006). No difference for lymph nodes metastases (13.5 vs 12.7 months P-value = 0.175). NLR ≥ 4 had worse OS (8.2 vs 17.7 months P-value = 0.0001). In multivariate analysis, BMI ≥ 30, bone metastases and NLR ≥ 4, performance status ≥ 2 and line of ICI ≥ 2 were independent factors for OS.

Conclusions: Our data identified BMI and bone metastasis as novel clinical biomarkers that were independently associated with ICI outcomes in mUC. External and prospective validation are warranted.

For peer review only - http://bmjopen.bmj.com/site/about/guidelines.xhtml

Strengths and limitations

- This is a multicenter cohort study, with a large number of patients, treated with ICI for mUC at 3 Canadian institutions.
- We identified reproductible clinical prognostic factors related with response and survival outcomes – including bone metastases for which there is limited data available from prospective studies.
- The retrospective study design may lead to selection bias related to population studied.

For peer review only - http://bmjopen.bmj.com/site/about/guidelines.xhtml

Introduction

Bladder cancer represents the 10th most common cancer in the world, with approximately 550,000 new cases annually, and accounts for 2.1% of all cancer deaths according to GLOBOCAN 2020[1]. There is a male predominance and smoking is the most common risk factor. In most cases, urothelial carcinomas arise from the urothelium of the bladder but can also develop from the upper urinary tract (renal pelvis and ureters). Tumors invading the detrusor muscle, ie., muscle invasive tumors, account with upfront metastatic disease in 25% of the cases [2]. Despite multimodality management for non-metastatic muscle invasive bladder cancer, 50% of these patients will relapse, with distant metastasis in most of the cases [3, 4]. Platinum-based chemotherapy remains the standard first line treatment for metastatic disease [3, 4] . Patients who respond or have stable disease following chemotherapy are eligible for subsequent maintenance therapy with the anti-PD-L1 immune checkpoint inhibitor, Avelumab [5], while those who fail to respond to chemotherapy subsequently can receive the anti-PD-1 immune checkpoint inhibitor, Pembrolizumab [6]. Other treatment options in the platinum-refractory setting include Enfortumab Vedotin and Erdafitinib in patients with susceptible Fibroblast Growth Factor Receptor (FGFR) alterations, and chemotherapy with taxanes (Paclitaxel and Docetaxel) or Vinflunine [7-10]. In the KEYNOTE-045 randomized phase 3 trial, Pembrolizumab showed a significantly longer OS of 10.3 months vs 7.4 months in patients with platinum refractory mUC compared to chemotherapy (Docetaxel, Paclitaxel or Vinflunine) (Hazard ratio HR 0.73; 95%Confidence interval CI, 0.59 to 0.91 P-value = 0.002), with a lower rate of any grade adverse events (60.9% vs 90.2%) [6]. Long term results confirmed an OS benefit and a favorable safety profile [11]. The programmed death ligand 1 (PD-L1) checkpoint inhibitor Atezolizumab was also associated with durable objective responses in the IMvigpr210 phase 2 trial, with favorable safety profile, [12] but failed to show survival benefit over chemotherapy in the IMvigor211 phase 3 trial [13]. In phase 1/2 trials

For peer review only

CheckMate275 and CheckMate032, Nivolumab, a programmed death 1 (PD-1) inhibitor, achieved objective responses alone or in combination with Ipilimumab, with a manageable adverse events profile [14, 15]. Despite responses seen with ICI in the platinum-refractory setting, not all patients will derive benefit and there is a lack of valid prognostic and predictive biomarkers.

In this study, we aimed to evaluate if clinical prognostic markers, that have been defined for other cancer types, are relevant to mUC. In fact, several retrospective studies have addressed the relationship between obesity and outcomes in patients receiving ICI. In melanoma, non-small cell lung cancer (NSCLC) and renal cell cancer (RCC), obesity appears to be linked to better progression free survival (PFS) and OS [16-18]. And a recent systematic review of 18 retrospective studies across different cancer types, by Indini at al., also found an association between high BMI and improved ICI outcomes; yet a strong positive correlation could not be concluded due to the heterogeneity of the studies [19]. As such, the relationship between BMI and immunotherapy outcomes in urothelial cancer remains poorly understood.

The tumor immune microenvironment of different metastatic locations may also affect the response to ICI [20, 21]. Retrospective studies reported differences in organ specific responses and organ specific OS with immunotherapy in several types of cancer [22-24].

Neutrophil-to-lymphocyte ratio (NLR) is an available marker of systemic response to inflammation, derived from the absolute counts of neutrophils and lymphocytes on a blood count [25]. It is a well-established poor prognostic factor in several cancers, independently of treatment type [25-27].

The aim of this retrospective analysis was to assess the association between high BMI, site of metastasis, NLR and outcome in terms of response and OS in a population of patients with mUC treated with ICI.

For peer review only - http://bmjopen.bmj.com/site/about/guidelines.xhtml

Patients and methods

Patient population, characteristics and outcome

We performed a multi-center retrospective cohort study analysis of mUC patients, from 3 Canadian cancer centers: Segal Cancer Center, Jewish General Hospital (JGH); Princess Margaret Cancer Center, University Health Network (PM-UHN) and Odette Cancer Center, Sunnybrook Health Sciences Center (SHSC). Patients with histologically proven metastatic urothelial cancer, who received at least one dose of anti-PD1/L1 ICI, between December 2016 and January 2021, were included regardless of gender, age and performance status. ICI at any line of treatment for metastatic disease, alone or in combination with chemotherapy oranother ICI, was allowed. Patients' characteristics were abstracted from chart review. The following clinical characteristics were collected: age, gender, smoking status, comorbidities, primary tumor location and histology, type and line of ICI treatment, number and type of previous treatment lines received, and sites of metastases. BMI at diagnosis, prior to ICI treatment and at progression was assessed. We selected 3 groups according to BMI at start of ICI: BMI < 25, $25 \leq$ BMI < 30 and BMI ≥ 30. We collected the following outcomes criteria: ICI ORR and OS. ICI ORR was determined by investigator assessment of radiologic response as per Response evaluation criteria in solid tumors (RECIST) and was the sum of complete (CR) and partial response (PR). OS was calculated from the start of ICI treatment to death or last follow-up.

Ethics approval

Ethics approval for this study was obtained from the Clinical Trials Ontario (CTO Project ID: 2067) after review by the UHN Research Ethics Board (REB) on August 19, 2020, for PM-UHN and SHSC sites. Ethics approval for the JGH site was approved by the integrated university health and social services centres (CIUSSS) West Central Montreal REB (Project 2022-2888) on October 28, 2022.

For peer review only - http://bmjopen.bmj.com/site/about/guidelines.xhtml

Statistical analysis

Fisher's exact test and chi square test were used to assess differences in response rates between the predefined groups. OS was assessed using Kaplan Meier. Log-rank was used to compare groups and Cox regression models were used to perform univariable and multivariable analysis. P-value < 0.05 were considered statistically significant. Clinical variables that were associated with a p-value<0.05 in the univariate analysis were included in the multivariable model. The final multivariable model included all variables with a p-value <0.05 in the multivariate analysis.

Patient and public involvement

Patients and/or the public were not involved in the design, conduct, reporting and dissemination plans of this research.

Results

Patients' characteristics

We identified 135 patients, who received at least one dose of ICI for mUC. The median age was 70 years (26-91 years). Most of the patients had a primary bladder cancer (n=125, 92.5%). Most patients (n=84, 62%) received ICI as a second line or later treatment for mUC. The median follow-up period was 14.5 months. Patients' characteristics for the entire cohort are shown in **Table 1**.

BMI data were available for 121 patients. 48.8% of the patients had a BMI ≥ 25. Median BMI was 20.9, 26.6 and 34.6 in groups BMI < 25, 25 ≤ BMI < 30 and BMI ≥ 30 respectively. At the time ICI treatment was initiated, metastatic sites were found as follows: lymph nodes 66.7% (n=90), lung 35.6% (n=48), bones 31.9% (n=43) and liver 27.4% (n=37).

The relationship between BMI and ICI response and survival outcomes

For peer review only - http://bmjopen.bmj.com/site/about/guidelines.xhtml

We observed differences in ORR according to the BMI category of the patient. The ORR was 45.4% in the BMI ≥ 25 group, versus 16.3% in the BMI < 25 group (P-value = 0.020) (**Figure 1a**) In the BMI ≥ 25 and BMI < 25 groups, we observed a higher proportion of CR to ICI (8 CR vs 1, 22 PR vs 8 and 7 SD vs 10) (**Supplemental table**). Patients with BMI ≥ 30 experienced the longest median OS (24.8 months) compared with those with 25 ≤ BMI < 30 (14.4 months) and BMI < 25 (8.5 months) (P-value = 0.012). (**Figure 1b**). BMI≥ 30 remained an independent prognostic variable in multivariable analysis (HR = 0.40; 95% CI 0.17-0.96; P-value=0.040) (**Table 2**).

Metastatic sites' response and survival outcomes

The ORR for the entire cohort was 32.2%. The ORR was significantly lower in patients with bone metastases 16% vs 41% (P = 0.006) (**Figure 2a**), or liver metastases 16% vs 39% (P-value = 0.013) (**Figure 2b**). Conversely, the presence of metastatic disease involving the lymph nodes was significantly correlated with higher ORR 40% vs 20% (P-value = 0.032) (**Figure 2c**). The difference in ORR for lung metastases was not statistically significant 27% vs 36% (P-value = 0.340) (**Figure 2d**). Detailed responses are listed in **supplemental table**. The median OS was evaluated for the entire cohort, for each metastatic site and for each BMI group. In the entire cohort, median OS was 12.7 months. Bone, liver and lung metastases correlated with significantly shorter survival. The median OS for patients with bone metastases was 7.3 months vs 18 months in the absence of bone metastases (P-value < 0.001) (**Figure 3a**). Patients with liver metastases had a median OS of 8.6 months compared to 15 months (P-value= 0.006) (**Figure 3b**), and 8.7 months compared to 17.3 months for those with lung metastases (P-value = 0.004) (**Figure 3c**). The presence of lymph node metastases was not significantly associated with OS: 13.5 months vs 12.7 months (P-value = 0.175) (**Figure 3d**). Although bone, lung and liver metastases were all associated with significantly worse OS in univariable analyses, bone

For peer review only - http://bmjopen.bmj.com/site/about/guidelines.xhtml

metastases was the only metastatic site that remained significantly associated with shorter OS in multi-variable analysis (HR = 1.98; 95%CI 1.17-3.35; P-value = 0.010) (**Table 2**).

NLR response and survival outcomes

Next, we evaluated the relationship between the NLR and clinical outcomes with ICI in this cohort. The ORR was not statistically different for patients with NLR ≥ 4 compared to those with NLR < 4, respectively 24.5% and 36.9% (P-value = 0.141) (**Supplemental Figure 1**). However, NLR < 4 was correlated with better OS, with a median of 17.7 months versus 8.2 months for NLR ≥ 4 (P-value < 0.001) (**Supplemental Figure 2**). The survival benefit associated with a low NLR at the time of initiating ICI therapy was independent of other prognostic variable in multivariable analysis (**Table 2**). We assessed whether there was a relationship between NLR and BMI, but we did not observe any significant correlation (data not shown).

Discussion

ICI are approved for a wide-array of cancers based on tumor type (melanoma, NSCLC, urothelial cancer) and/or in a tumor type agnostic manner based on the presence of molecular biomarkers such as high tumor mutation burden [28-30] and microsatellite instability [30-32]. However, in most cases, many patients who are eligible for immunotherapies based on tumor type or biomarker status will either not respond to treatment or will eventually become resistant to treatment. In this analysis, we identified several important clinical biomarkers that associate with better (high BMI) or worse (bone metastases, high NLR) outcomes with ICI in patients with mUC. These biomarkers are easily assessed without any additional costly molecular testing. Moreover, ICIs are expensive, potentially toxic and in mUC only 30% of the patients benefit from ICIs. As such, we believe that these data may be clinically useful to guide treatment decisions.

For peer review only - http://bmjopen.bmj.com/site/about/guidelines.xhtml

According to the World Health Organization (WHO) obesity, classified as a BMI greater than 30 kg/m^2, is a rising epidemic [33]. Our data showed strong correlation between elevated BMI and improved outcome in terms of response as well as survival. To the best of our knowledge, the present study is the largest to report data of ICI outcomes correlation with BMI in mUC [19, 34, 35]. A favorable prognostic role of high BMI was also reported in RCC, NSCLC and melanoma [17, 34, 36-38]. A pooled post hoc analysis of individual participant data from 4 prospective trials with Atezolizumab in metastatic NSCLC showed significant difference in survival between normal weight, overweight and obese patients with improved OS for patients with obesity and overweight compared to normal weight. [17] In a retrospective multicohort analysis of 1918 metastatic melanoma patients treated with chemotherapy, targeted therapies and ICI, McQuade et al studied association between BMI and outcome, as well as interactions between BMI and gender and type of treatment. Obesity was associated with improved OS and PFS with a benefit restricted to patients treated with targeted therapies and ICI [16].

There is increasing evidence that excess body weight is a modifiable risk factor for several malignancies, including pancreas, kidney, colorectal, postmenopausal breast, ovarian, gallbladder and thyroid cancers [39]. However, obesity is not an established risk factor for bladder cancer and the impact of obesity on cancer survival is less clear. Some studies have reported that obesity is associated with improved survival in some, but not all, patients with cancer, which is referred to the "obesity paradox" [40]. Numerous studies have shown that obesity can cause immune cell dysfunction [41-44], while others argue that the chronic low-grade inflammation underpinning obesity creates a pro-inflammatory state that synergizes with immunotherapy [45, 46].

Pre-clinical and translational research has provided insight into how immune checkpoint blockade can benefit obese patients. Obesity appears to influence T cell function and phenotype. For example, leptin-associated T cell dysfunction due to obesity has been observed

For peer review only - http://bmjopen.bmj.com/site/about/guidelines.xhtml

across different species and tumor models and was found to enhance the immune response to anti-PD-1 therapy [41]. In a mouse study, Shirakawa et al. reported that obesity caused a preferential increase and accumulation of senescent $CD44^{hi}CD62L^{lo}CD4+$ T cells that constitutively express PD-1 and CD153 [47]. Moreover, a study in a colon carcinoma mouse model showed that obesity impaired the infiltration, cytokine production and metabolic activity of tumor-infiltrating CD8 T cells, while anti-PD-1 restored CD8 T cells infiltration, proinflammatory cytokine production and metabolic activity, leading to complete tumor eradication and immune memory [48]. Obesity may also affect other immune cells in the tumor microenvironment (TME), such as myeloid-derived suppressor cells (MDSCs), which are known to suppress T cell responses. MDSCs can suppress T cell activity through many mechanisms, one of which is expressing PD-L1 to induce T cell exhaustion [49, 50]. In a pre-clinical study, inhibiting PD-L1 on MDSCs decreased their ability to suppress T cell activity, suggesting that obesity could also accelerate tumor progression by promoting the expression of PD-L1 on MDSCs [51]. Finally, a preclinical study in a breast cancer model showed that although obesity accelerated tumor progression, anti-PD-1 treatment significantly reduced tumor burden by reshaping the local TME landscape [52].

The relationship between obesity and ICI is complex and depends on various of factors, including the types of cancer and ICI involved. However, BMI may be an imperfect marker and its evaluation based only on weight prior to ICI start can be limiting. Assessment of the weight loss or change over the time may be a better indicator of disease prognosis. As BMI cannot distinguish body fat from muscle, it may not be the best tool to assess obesity [53]. Other measurement methods could be used for better assessment such as dual energy x-ray absorptiometry or fat referenced quantitative MRI [54].

For peer review only - http://bmjopen.bmj.com/site/about/guidelines.xhtml

Further studies are needed to understand the molecular mechanisms underlying the interaction between obesity and cancer immunity and to identify potential targets for effective interventions.

Bone metastases are a validated negative prognostic factor in mUC treated with platinum based chemotherapy [55, 56]. In our study, poor response and survival outcome of bone and liver metastases was significant, while lymph nodes metastases correlated with higher ORR. These results are consistent with published data in the literature for patients treated with ICI [57-59]. Makrakis et al. showed lower response rates and shorter OS for bone and liver metastases on retrospective data from 917 mUC treated with ICI as 1st or ≥ 2nd line, but higher ORR for lymph node confined metastases [57]. A retrospective multicentric Japanese study reported shorter ORR for bone metastases in a cohort of patients treated with Pembrolizumab for mUC [58]. Similar results have also been reported from prospective trials [60, 61]. In the IMvigor210 phase 2 trial, the ORR on Atezolizumab was 32% for lymph nodes confined metastases, and only 8% for liver metastases, data for bone metastases were not reported [60]. Higher ORR for lymph nodes metastases (47%) compared to liver metastases (23%) was also reported in the Keynote-052 trial [61]. Prognostic models for mUC patients treated with ICI identified liver or visceral metastases as poor prognostic factors [62, 63]. Sonpavde et al. developed a five-factor prognostic model for survival for mUC treated with Atezolizumab, Avelumab or Durvalumab, based on the data from phase 1 and 2 trials. [64] They identified 3 risk groups based on performance status, liver metastases, platelet count, NLR and lactate dehydrogenase levels (LDH) as prognostic factors. [64] The 1-year Kaplan-Meier survival estimates of those in the low, intermediate and poor risk groups were 76.2%, 33.8 % and 8.6%, respectively. [64] The authors developed an interactive web-based tool to calculate the expected survival probabilities according to the five-factors. [64] In our study, LDH levels were not uniformly assessed prior to ICI treatment at all sites, and we did not collect data for platelet count. Therefore, we could

For peer review only - http://bmjopen.bmj.com/site/about/guidelines.xhtml

not compare the prognostic utility of this tool in our cohort of patients. Most prospective trials of ICI in mUC do not use presence/absence of bone metastases as a stratification factor, despite the fact that bone metastases are associated with poor response to chemotherapy and impact quality of life [55, 56, 65]. Owari et al validated a specific prognostic scoring system, B-FOM, to predict survival for patients with bone metastasis from different genito-urinary cancers based on 5 prognostic factors: primary tumor (prostate, renal or urothelial cancers), poor performance status, visceral metastases, high Glasgow-prognostic score and elevated NLR [66]. This prediction tool may be helpful to individualize optimal treatment strategy for the patients. A better understanding of the bone microenvironment is crucial to help the development of novel therapeutic strategies and improve outcomes [67].

The distant organ microenvironment, also known as metastatic microenvironment (MME), plays an important role in site-specific metastases [68]. For instance, macrophages, which are abundant immune cells in the lungs, can bind to cancer cells via receptor VCAM-1 transmit, signaling a chain of events leading to lung-specific metastases in breast cancer [69]. It has been demonstrated that myeloid cells can remodel the pre-metastatic lung from an immune protective state to a state favoring tumor progression, thereby promoting lung metastases [70]. Furthermore, lung stromal cells can also promote tumor colonization and metastasis by secreting periostin [71]. In the liver, there is a significant presence of circulating and resident natural killer (NK) cells, which serve as the primary effectors of liver immune function. This NK cell maintained the breast cancer cells dormant in the liver by secreting Interferonγ (IFNγ), while sustaining NK cell abundance with Interleukine15 (IL15) based immunotherapy succeeded to prevent liver metastases and prolong the survival in preclinical models [72]. Conversely, bone forms an immunosuppressive environment mainly due to immature NK cells, a small number of cytotoxic T cells, a large number of myeloid progenitors and Treg [73]. In a human bladder tumor xenografts model, researchers observed a high infiltration of bone

For peer review only - http://bmjopen.bmj.com/site/about/guidelines.xhtml

marrow-derived host CD11b myeloid cells. They further demonstrated that enhanced tumor-associated in the TME promote an immunosuppressive pro-tumoral myeloid phenotype [74]. These observations demonstrate how the MME can influence the metastatic potential and outcome of different types of cancer cells in different organs, suggesting that MME might also potentiate site-specific metastases in bladder cancer. Taken together, all these studies suggest the importance of understanding the metastatic potential and outcome of different types of cancer cells in different organs, through which we can further develop more effective therapeutic measures against bladder cancer.

NLR is a well-established poor prognostic factor in several cancers, independently of treatment type [25-27]. It could be considered as a surrogate marker of chronic inflammation and evasion of immune surveillance [75]. In our study, high NLR was associated with shorter median OS. A recent study by Valero et al showed poor response and survival outcomes, for 1917 patients with high NLR at diagnosis, treated with ICI for multiple cancer types [75]. Similar results were reported for melanoma and NSCLC [26, 76]. Banna et al explored the prognostic and predictive role of NLR and LDH in metastatic urothelial cancers treated with ICI and showed significant correlation with PFS and OS for high NLR [77]. For patients treated with ICI for mUC, a high NLR prior to first line chemotherapy and second line pembrolizumab was associated with worse survival outcomes [78]. NLR may represent an accessible low-cost predictive biomarker.

The present study has some strengths and limitations. It presents a relatively large multi-center cohort of patients with significant prognostic factors. On the other hand, this is a retrospective study, with heterogenous population and an investigator-based response assessment on clinical and radiological chart review. However, the observations made in this analysis are hypothesis generating. The evaluation of obesity and its relationship with ICI response warrants future prospective studies that incorporate better established measures of obesity and metabolic

For peer review only - http://bmjopen.bmj.com/site/about/guidelines.xhtml

syndrome. Moreover, little is known about how bladder cancer cells interact with the liver and bone TME and this warrants further investigation in preclinical models to develop optimal immunotherapies for patients with bone and liver mUC.

Conclusion

Our study identified elevated BMI, NLR and presence of bone metastases as potential biomarkers for ICI response and survival in mUC. Obesity was associated with improved survival and response rate, whereas bone metastases and high NLR are associated with lack of response and shorter survival. Prospective validation of these data is warranted, especially in the evolving landscape of therapeutic options for mUC with novel agents and combinations with ICI [79].

For peer review only

For peer review only - http://bmjopen.bmj.com/site/about/guidelines.xhtml

Contributorship statement

Guarantor of integrity of the entire study: April A.N Rose, Soumaya Labidi

Study concepts and design: Adrian G. Sacher, April A. N. Rose

Literature research: Soumaya Labidi, Mengqi Li

Data collection: Soumaya Labidi, Nicholas Meti, Reeta Barua

Statistical analysis: Soumaya Labidi, April A.N Rose

Manuscript preparation: Soumaya Labidi, Mengqi Li, April A.N Rose

Manuscript editing and critical analysis: Nicholas Meti, Di Maria Jiang, Srikala S. Sridhar, Sonia V. del Rincon, April A. N. Rose

All authors were involved in data interpretation, critical appraisal of the draft manuscript and gave final approval on the submitted version.

Competing interests

Nicholas Meti: Honoraria: Takeda, Novartis. Consulting: Takeda, Seagen, AstraZeneca, Pfizer

Srikala S. Sridhar: Consulting: Astellas, AstraZeneca, Bayer, Bicycle Therapeutics, BMS, Eisai, EMD Serono, Gilead, Ipsen, Janssen, Merck, Pfizer, Seagen
Research funding (Institution): Janssen, Seagen, EMD Serono

Rossanna C. Pezo: Honoraria: Pfizer, Novartis, BMS, Merck, Sanofi, Gilead, AstraZeneca
Research funding (Institution): Merck, Novartis, Pfizer, Taiho, Jazz Pharmaceuticals, ZymeWorks, AstraZeneca

Cristiano Ferrario: Honoraria: Pfizer, Bayer, Novartis, Roche, Bayer, Knight therapeutics, AstraZeneca, Merck, Astellas Pharma. Consulting: Merck, Novartis, AstraZeneca, Roche. Research funding (Institution): Merck, Novartis, Pfizer, Lilly, ZymeWorks, AstraZeneca, Janssen Oncology, Bayer, Astellas Pharma, Roche, Sanofi, Seattle Genetics, Semonix Pharmaceuticals, Bicycle Therapeutics, Immunomedics

Susanna Cheng: Consulting: AstraZeneca, Merck

April A.N. Rose: Consulting: EMD Serono, Advanced accelerator applications/Novartis. Research funding (Personal): Canadian Institutes of Health Research CIHR, Canadian Cancer Society, Conquer Cancer Foundation, Jewish General Hospital Foundation, TransMedTech Institute, Canada Foundation of Innovation. Research Funding (Institution): AstraZeneca, Merck, Pfizer, Seattle Genetics. Employment: Family member Merck

Soumaya Labidi, Mengqi Li, Sonia V. Del Rincon, Reeta Barua, Jamila Riromar, Di Maria Jiang, Nazanin Fallah-Rad, Adrian G. Sacher: No competing interests

Funding

April A.N. Rose acknowledges support from a Fonds de Recherche du Québec – Santé (FRQS) Clinical Research Scholar Award. This research was supported by a University of Toronto, Medical Oncology Strategic Planning Medical Oncology Innovation Fund to Adrian G. Sacher

and April A.N. Rose and funding from the Jewish General Hospital (JGH) Foundation to April A.N. Rose.

Data sharing statement

All data relevant to the study are included in the article or uploaded as supplementary information. The raw data can not be shared.

For peer review only

REFERENCES

1. Sung, H., et al., *Global Cancer Statistics 2020: GLOBOCAN Estimates of Incidence and Mortality Worldwide for 36 Cancers in 185 Countries.* CA Cancer J Clin, 2021. **71**(3): p. 209-249.
2. Humphrey, P.A., et al., *The 2016 WHO Classification of Tumours of the Urinary System and Male Genital Organs—Part B: Prostate and Bladder Tumours.* European Urology, 2016. **70**(1): p. 106-119.
3. Nadal, R. and J. Bellmunt, *Management of metastatic bladder cancer.* Cancer Treatment Reviews, 2019. **76**: p. 10-21.
4. Witjes, J.A., et al., *European Association of Urology Guidelines on Muscle-invasive and Metastatic Bladder Cancer: Summary of the 2020 Guidelines.* Eur Urol, 2021. **79**(1): p. 82-104.
5. Powles, T., et al., *Avelumab Maintenance Therapy for Advanced or Metastatic Urothelial Carcinoma.* N Engl J Med, 2020. **383**(13): p. 1218-1230.
6. Bellmunt, J., et al., *Pembrolizumab as Second-Line Therapy for Advanced Urothelial Carcinoma.* N Engl J Med, 2017. **376**(11): p. 1015-1026.
7. McCaffrey, J.A., et al., *Phase II trial of docetaxel in patients with advanced or metastatic transitional-cell carcinoma.* J Clin Oncol, 1997. **15**(5): p. 1853-7.
8. Dreicer, R., et al., *Paclitaxel in advanced urothelial carcinoma: its role in patients with renal insufficiency and as salvage therapy.* J Urol, 1996. **156**(5): p. 1606-8.
9. Ko, Y.J., et al., *Nanoparticle albumin-bound paclitaxel for second-line treatment of metastatic urothelial carcinoma: a single group, multicentre, phase 2 study.* Lancet Oncol, 2013. **14**(8): p. 769-76.
10. Bellmunt, J., et al., *Long-term survival results of a randomized phase III trial of vinflunine plus best supportive care versus best supportive care alone in advanced urothelial carcinoma patients after failure of platinum-based chemotherapy.* Ann Oncol, 2013. **24**(6): p. 1466-72.
11. Fradet, Y., et al., *Randomized phase III KEYNOTE-045 trial of pembrolizumab versus paclitaxel, docetaxel, or vinflunine in recurrent advanced urothelial cancer: results of >2 years of follow-up.* Ann Oncol, 2019. **30**(6): p. 970-976.
12. Rosenberg, J.E., et al., *Atezolizumab in patients with locally advanced and metastatic urothelial carcinoma who have progressed following treatment with platinum-based chemotherapy: a single-arm, multicentre, phase 2 trial.* Lancet, 2016. **387**(10031): p. 1909-20.
13. Powles, T., et al., *Atezolizumab versus chemotherapy in patients with platinum-treated locally advanced or metastatic urothelial carcinoma (IMvigor211): a multicentre, open-label, phase 3 randomised controlled trial.* Lancet, 2018. **391**(10122): p. 748-757.
14. Sharma, P., et al., *Nivolumab in metastatic urothelial carcinoma after platinum therapy (CheckMate 275): a multicentre, single-arm, phase 2 trial.* Lancet Oncol, 2017. **18**(3): p. 312-322.
15. Sharma, P., et al., *Nivolumab Alone and With Ipilimumab in Previously Treated Metastatic Urothelial Carcinoma: CheckMate 032 Nivolumab 1 mg/kg Plus Ipilimumab 3 mg/kg Expansion Cohort Results.* J Clin Oncol, 2019. **37**(19): p. 1608-1616.
16. McQuade, J.L., et al., *Association of body-mass index and outcomes in patients with metastatic melanoma treated with targeted therapy, immunotherapy, or chemotherapy: a retrospective, multicohort analysis.* Lancet Oncol, 2018. **19**(3): p. 310-322.
17. Kichenadasse, G., et al., *Association Between Body Mass Index and Overall Survival With Immune Checkpoint Inhibitor Therapy for Advanced Non-Small Cell Lung Cancer.* JAMA Oncol, 2020. **6**(4): p. 512-518.
18. Albiges, L., et al., *Body Mass Index and Metastatic Renal Cell Carcinoma: Clinical and Biological Correlations.* J Clin Oncol, 2016. **34**(30): p. 3655-3663.

For peer review only - http://bmjopen.bmj.com/site/about/guidelines.xhtml

19. Indini, A., et al., *Impact of BMI on Survival Outcomes of Immunotherapy in Solid Tumors: A Systematic Review.* Int J Mol Sci, 2021. **22**(5).
20. Ganesh, K. and J. Massague, *Targeting metastatic cancer.* Nat Med, 2021. **27**(1): p. 34-44.
21. Oliver, A.J., et al., *Tissue-Dependent Tumor Microenvironments and Their Impact on Immunotherapy Responses.* Front Immunol, 2018. **9**: p. 70.
22. Furubayashi, N., et al., *Organ-Specific Tumor Response to Pembrolizumab in Advanced Urothelial Carcinoma After Platinum-Based Chemotherapy.* Onco Targets Ther, 2021. **14**: p. 1981-1988.
23. Tumeh, P.C., et al., *Liver Metastasis and Treatment Outcome with Anti-PD-1 Monoclonal Antibody in Patients with Melanoma and NSCLC.* Cancer Immunol Res, 2017. **5**(5): p. 417-424.
24. Schmid, S., et al., *Organ-specific response to nivolumab in patients with non-small cell lung cancer (NSCLC).* Cancer Immunol Immunother, 2018. **67**(12): p. 1825-1832.
25. Guthrie, G.J., et al., *The systemic inflammation-based neutrophil-lymphocyte ratio: experience in patients with cancer.* Crit Rev Oncol Hematol, 2013. **88**(1): p. 218-30.
26. Cedres, S., et al., *Neutrophil to lymphocyte ratio (NLR) as an indicator of poor prognosis in stage IV non-small cell lung cancer.* Clin Transl Oncol, 2012. **14**(11): p. 864-9.
27. Templeton, A.J., et al., *Prognostic role of neutrophil-to-lymphocyte ratio in solid tumors: a systematic review and meta-analysis.* J Natl Cancer Inst, 2014. **106**(6): p. dju124.
28. Kim, S.I., C.R. Cassella, and K.T. Byrne, *Tumor Burden and Immunotherapy: Impact on Immune Infiltration and Therapeutic Outcomes.* Front Immunol, 2020. **11**: p. 629722.
29. Samstein, R.M., et al., *Tumor mutational load predicts survival after immunotherapy across multiple cancer types.* Nat Genet, 2019. **51**(2): p. 202-206.
30. Palmeri, M., et al., *Real-world application of tumor mutational burden-high (TMB-high) and microsatellite instability (MSI) confirms their utility as immunotherapy biomarkers.* ESMO Open, 2022. **7**(1): p. 100336.
31. O'Malley, D.M., et al., *Pembrolizumab in Patients With Microsatellite Instability-High Advanced Endometrial Cancer: Results From the KEYNOTE-158 Study.* J Clin Oncol, 2022. **40**(7): p. 752-761.
32. Maio, M., et al., *Pembrolizumab in microsatellite instability high or mismatch repair deficient cancers: updated analysis from the phase II KEYNOTE-158 study.* Ann Oncol, 2022. **33**(9): p. 929-938.
33. Jensen, M.D., et al., *2013 AHA/ACC/TOS guideline for the management of overweight and obesity in adults: a report of the American College of Cardiology/American Heart Association Task Force on Practice Guidelines and The Obesity Society.* Circulation, 2014. **129**(25 Suppl 2): p. S102-38.
34. Cortellini, A., et al., *A multicenter study of body mass index in cancer patients treated with anti-PD-1/PD-L1 immune checkpoint inhibitors: when overweight becomes favorable.* Journal for ImmunoTherapy of Cancer, 2019. **7**(1).
35. Yoo, S.K., et al., *Outcomes Among Patients With or Without Obesity and With Cancer Following Treatment With Immune Checkpoint Blockade.* JAMA Netw Open, 2022. **5**(2): p. e220448.
36. Martini, D.J., et al., *Adiposity may predict survival in patients with advanced stage cancer treated with immunotherapy in phase 1 clinical trials.* Cancer, 2020. **126**(3): p. 575-582.
37. Rutkowski, P., et al., *Body mass index (BMI) and outcome of metastatic melanoma patients receiving targeted therapy and immunotherapy: a multicenter international retrospective study.* Journal for ImmunoTherapy of Cancer, 2020. **8**(2): p. e001117.
38. Richtig, G., et al., *Body mass index may predict the response to ipilimumab in metastatic melanoma: An observational multi-centre study.* PLOS ONE, 2018. **13**(10): p. e0204729.
39. Lauby-Secretan, B., et al., *Body fatness and cancer—viewpoint of the IARC Working Group.* New England journal of medicine, 2016. **375**(8): p. 794-798.

For peer review only - http://bmjopen.bmj.com/site/about/guidelines.xhtml

40. Lennon, H., et al., *The obesity paradox in cancer: a review.* Current oncology reports, 2016. **18**: p. 1-8.
41. Wang, Z., et al., *Paradoxical effects of obesity on T cell function during tumor progression and PD-1 checkpoint blockade.* Nature medicine, 2019. **25**(1): p. 141-151.
42. Rebeles, J., et al., *Obesity-induced changes in T-cell metabolism are associated with impaired memory T-cell response to influenza and are not reversed with weight loss.* The Journal of Infectious Diseases, 2019. **219**(10): p. 1652-1661.
43. Gibson, J.T., et al., *Obesity-associated myeloid-derived suppressor cells promote apoptosis of tumor-infiltrating CD8 T cells and immunotherapy resistance in breast cancer.* Frontiers in immunology, 2020. **11**: p. 590794.
44. Ringel, A.E., et al., *Obesity shapes metabolism in the tumor microenvironment to suppress anti-tumor immunity.* Cell, 2020. **183**(7): p. 1848-1866. e26.
45. Wang, Z., A.M. Monjazeb, and W.J. Murphy, *The complicated effects of obesity on cancer and immunotherapy*. 2019, Future Medicine. p. 11-14.
46. Fang, H., et al., *Regulating the Obesity-Related Tumor Microenvironment to Improve Cancer Immunotherapy.* ACS nano, 2023. **17**(5): p. 4748-4763.
47. Shirakawa, K., et al., *Obesity accelerates T cell senescence in murine visceral adipose tissue.* The Journal of clinical investigation, 2016. **126**(12): p. 4626-4639.
48. Dyck, L., et al., *Suppressive effects of the obese tumor microenvironment on CD8 T cell infiltration and effector function.* Journal of Experimental Medicine, 2022. **219**(3).
49. Lau, J., et al., *Tumour and host cell PD-L1 is required to mediate suppression of anti-tumour immunity in mice.* Nature communications, 2017. **8**(1): p. 14572.
50. Weber, R., et al., *Myeloid-derived suppressor cells hinder the anti-cancer activity of immune checkpoint inhibitors.* Frontiers in immunology, 2018. **9**: p. 1310.
51. Hale, M., et al., *Obesity triggers enhanced MDSC accumulation in murine renal tumors via elevated local production of CCL2.* PloS one, 2015. **10**(3): p. e0118784.
52. Pingili, A.K., et al., *Immune checkpoint blockade reprograms systemic immune landscape and tumor microenvironment in obesity-associated breast cancer.* Cell reports, 2021. **35**(12): p. 109285.
53. Arnett, D.K., et al., *2019 ACC/AHA Guideline on the Primary Prevention of Cardiovascular Disease: A Report of the American College of Cardiology/American Heart Association Task Force on Clinical Practice Guidelines.* J Am Coll Cardiol, 2019. **74**(10): p. e177-e232.
54. Borga, M., et al., *Advanced body composition assessment: from body mass index to body composition profiling.* J Investig Med, 2018. **66**(5): p. 1-9.
55. Alqaisi, H.A., et al., *The prognostic impact of bone metastasis in patients with metastatic urothelial carcinoma treated with first-line platinum-based chemotherapy.* Ther Adv Med Oncol, 2022. **14**: p. 17588359221094879.
56. Necchi, A., et al., *Nomogram-based Prediction of Overall Survival in Patients with Metastatic Urothelial Carcinoma Receiving First-line Platinum-based Chemotherapy: Retrospective International Study of Invasive/Advanced Cancer of the Urothelium (RISC).* Eur Urol, 2017. **71**(2): p. 281-289.
57. Makrakis, D., et al., *Association Between Sites of Metastasis and Outcomes With Immune Checkpoint Inhibitors in Advanced Urothelial Carcinoma.* Clin Genitourin Cancer, 2022. **20**(5): p. e440-e452.
58. Shimizu, T., et al., *Organ-Specific and Mixed Responses to Pembrolizumab in Patients with Unresectable or Metastatic Urothelial Carcinoma: A Multicenter Retrospective Study.* Cancers (Basel), 2022. **14**(7).
59. Raggi, D., et al., *Role of Bone Metastases in Patients Receiving Immunotherapy for Pre-Treated Urothelial Carcinoma: The Multicentre, Retrospective Meet-URO-1 Bone Study.* Clin Genitourin Cancer, 2022. **20**(2): p. 155-164.

For peer review only - http://bmjopen.bmj.com/site/about/guidelines.xhtml

60. Balar, A.V., et al., *Atezolizumab as first-line treatment in cisplatin-ineligible patients with locally advanced and metastatic urothelial carcinoma: a single-arm, multicentre, phase 2 trial.* Lancet, 2017. **389**(10064): p. 67-76.
61. Balar, A.V., et al., *First-line pembrolizumab in cisplatin-ineligible patients with locally advanced and unresectable or metastatic urothelial cancer (KEYNOTE-052): a multicentre, single-arm, phase 2 study.* Lancet Oncol, 2017. **18**(11): p. 1483-1492.
62. Nassar, A.H., et al., *A model combining clinical and genomic factors to predict response to PD-1/PD-L1 blockade in advanced urothelial carcinoma.* Br J Cancer, 2020. **122**(4): p. 555-563.
63. Khaki, A.R., et al., *A New Prognostic Model in Patients with Advanced Urothelial Carcinoma Treated with First-line Immune Checkpoint Inhibitors.* Eur Urol Oncol, 2021. **4**(3): p. 464-472.
64. Sonpavde, G., et al., *Five-Factor Prognostic Model for Survival of Post-Platinum Patients with Metastatic Urothelial Carcinoma Receiving PD-L1 Inhibitors.* J Urol, 2020. **204**(6): p. 1173-1179.
65. Stellato, M., et al., *Bone metastases from urothelial carcinoma. The dark side of the moon.* J Bone Oncol, 2021. **31**: p. 100405.
66. Owari, T., et al., *External validation of a genitourinary cancer-specific prognostic scoring system to predict survival for patients with bone metastasis (modified B-FOM scoring model): Comparison with other scoring models in terms of accuracy.* J Bone Oncol, 2021. **26**: p. 100344.
67. Reinstein, Z.Z., et al., *Overcoming immunosuppression in bone metastases.* Crit Rev Oncol Hematol, 2017. **117**: p. 114-127.
68. Liu, Q., et al., *Factors involved in cancer metastasis: a better understanding to "seed and soil" hypothesis.* Molecular Cancer, 2017. **16**(1): p. 176.
69. Chen, Q., X.H.-F. Zhang, and J. Massagué, *Macrophage binding to receptor VCAM-1 transmits survival signals in breast cancer cells that invade the lungs.* Cancer cell, 2011. **20**(4): p. 538-549.
70. Yan, H.H., et al., *Gr-1+ CD11b+ myeloid cells tip the balance of immune protection to tumor promotion in the premetastatic lung.* Cancer research, 2010. **70**(15): p. 6139-6149.
71. Malanchi, I., et al., *Interactions between cancer stem cells and their niche govern metastatic colonization.* Nature, 2012. **481**(7379): p. 85-89.
72. Correia, A.L., et al., *Hepatic stellate cells suppress NK cell-sustained breast cancer dormancy.* Nature, 2021. **594**(7864): p. 566-571.
73. Zhao, E., et al., *Bone marrow and the control of immunity.* Cellular & molecular immunology, 2012. **9**(1): p. 11-19.
74. Eruslanov, E., et al., *Aberrant PGE2 metabolism in bladder tumor microenvironment promotes immunosuppressive phenotype of tumor-infiltrating myeloid cells.* International immunopharmacology, 2011. **11**(7): p. 848-855.
75. Valero, C., et al., *Pretreatment neutrophil-to-lymphocyte ratio and mutational burden as biomarkers of tumor response to immune checkpoint inhibitors.* Nat Commun, 2021. **12**(1): p. 729.
76. Capone, M., et al., *Baseline neutrophil-to-lymphocyte ratio (NLR) and derived NLR could predict overall survival in patients with advanced melanoma treated with nivolumab.* J Immunother Cancer, 2018. **6**(1): p. 74.
77. Banna, G.L., et al., *Neutrophil-to-lymphocyte ratio and lactate dehydrogenase as biomarkers for urothelial cancer treated with immunotherapy.* Clin Transl Oncol, 2020. **22**(11): p. 2130-2135.
78. Kobayashi, T., et al., *Pre-pembrolizumab neutrophil-to-lymphocyte ratio (NLR) predicts the efficacy of second-line pembrolizumab treatment in urothelial cancer regardless of the pre-chemo NLR.* Cancer Immunol Immunother, 2022. **71**(2): p. 461-471.

For peer review only - http://bmjopen.bmj.com/site/about/guidelines.xhtml

79. Hoimes, C.J., et al., *Enfortumab Vedotin Plus Pembrolizumab in Previously Untreated Advanced Urothelial Cancer.* J Clin Oncol, 2023. **41**(1): p. 22-31.

For peer review only

For peer review only - http://bmjopen.bmj.com/site/about/guidelines.xhtml

Table 1: Patients' characteristics

Clinical Characteristic	N (%)
Age	
Age ≤ 60 years	28 (20.7%)
Age > 60 years	107 (79.3%)
Gender	
Male	101 (74.8%)
Female	34 (25.2%)
Smoking status	
Ever smoker	69 (51.1%)
Never smoker	60 (44.4%)
Missing Data	6 (4.5%)
Primary Tumor Site	
Bladder	125 (92.6%)
Upper tract	10 (7.4%)
Pathology	
Urothelial	123 (91.1%)
Squamous	7 (5.2%)
Other	5 (3.7%)
ECOG[a]	
0-1	112 (83%)
≥ 2	15 (11.1%)
Missing data	8 (5.9%)
Metastatic sites[#]	
LN[b]	90 (66.7%)
Lung	48 (35.6%)
Bone	43 (31.9%)
Liver	37 (27.4%)
BMI[c]	
< 25	55 (40.7%)
25-30	43 (31.9%)
≥ 30	23 (17.0%)
Missing data	14 (10.4%)
ICI[d] *Line*	
First	51 (37.8%)
Second or later	84 (62.2%)

[a]Eastern Cooperative Oncology Group Performance Status Scale [b]Lymph Nodes [c]Body Mass Index [d]Immune Checkpoint Inhibitors

[#]Numbers do not add up to 100% because many patients had multiple sites of metastases

For peer review only - http://bmjopen.bmj.com/site/about/guidelines.xhtml

Table 2: Univariable and Multivariable analysis for overall survival

OS	Univariable			Multivariable		
	HR	95% CI	p-value	HR	95% CI	p-value
BMI[a] ≥ 30 vs < 30	0.63	0.45-0.88	0.012	0.40	0.17-0.96	0.040
Male vs Female	0.95	0.58-1.57	0.867			
Age ≥ 60 vs < 60	1.88	0.65-5.45	0.235			
ECOG[b] ≥ 2 vs 0-1	1.68	0.86-3.29	0.125	2.21	1.02-4.78	0.042
ICI[c] Line ≥ 2 vs 1	2.39	1.43-3.97	0.001	1.80	1.31-2.48	0.000
NLR[d] ≥ 4 vs < 4	2.46	1.53-3.94	0.000	2.66	1.58-4.49	0.000
Bone metastasis Present vs Absent	2.38	1.52-3.73	0.000	1.98	1.17-3.35	0.010
Lung metastasis Present vs Absent	1.88	1.21-2.93	0.005			
Liver metastasis Present vs Absent	1.92	1.2-3.09	0.006			

[a]Body mass index [b]Eastern cooperative oncology group performance status scale [c]Immune checkpoint inhibitor [d]Neutrophil-to-lymphocyte ratio

Figure 1 Overall response rate (A) and overall survival (B) according to body mass index BMI

Figure 2 Overall response rate according to metastatic sites (A) Bone (B) Liver (C) Lymph nodes (D) Lung

Figure 3 Overall survival according to metastatic sites (A) Bone (B) Liver (C) Lung (D) Lymph nodes

Supplemental Figure 1 Overall response rate according to neutrophil to lymphocyte ratio NLR

Supplemental Figure 2 Overall survival according to NLR

Supplemental Figure 3 Overall response rate and overall survival according to BMI (A), bone metastases (B) and NLR (C) in the cohort of patients treated with immune checkpoint inhibitors alone

For peer review only

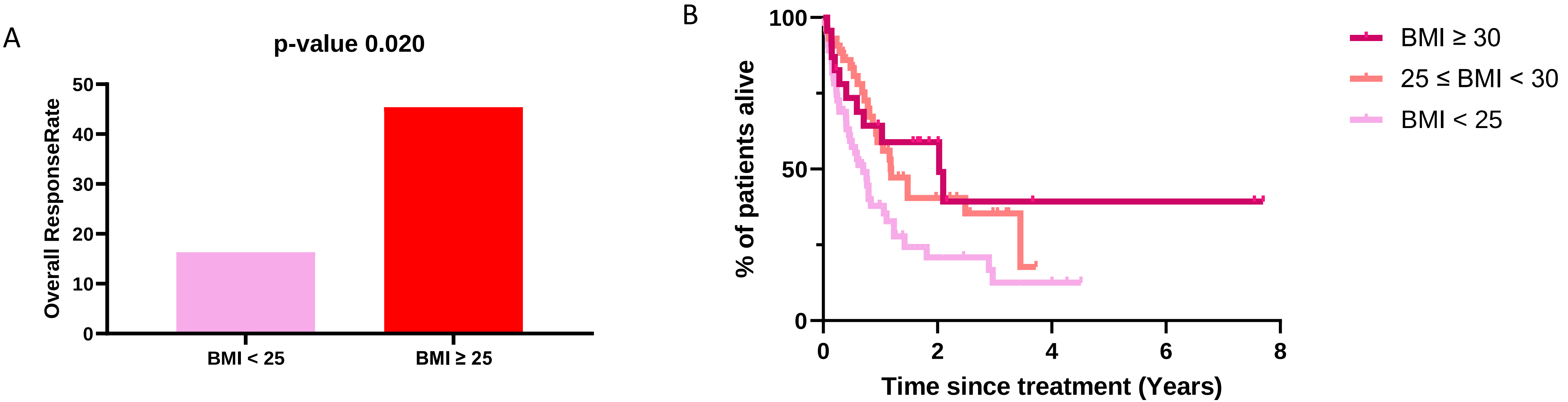

Number at risk					
BMI ≥ 30	23	6	2	2	0
25 ≤ BMI < 30	43	9	0	0	0
BMI < 25	55	5	0	0	0

Figure 1. Overall response rate and overall survival according to body mass index (BMI) groups

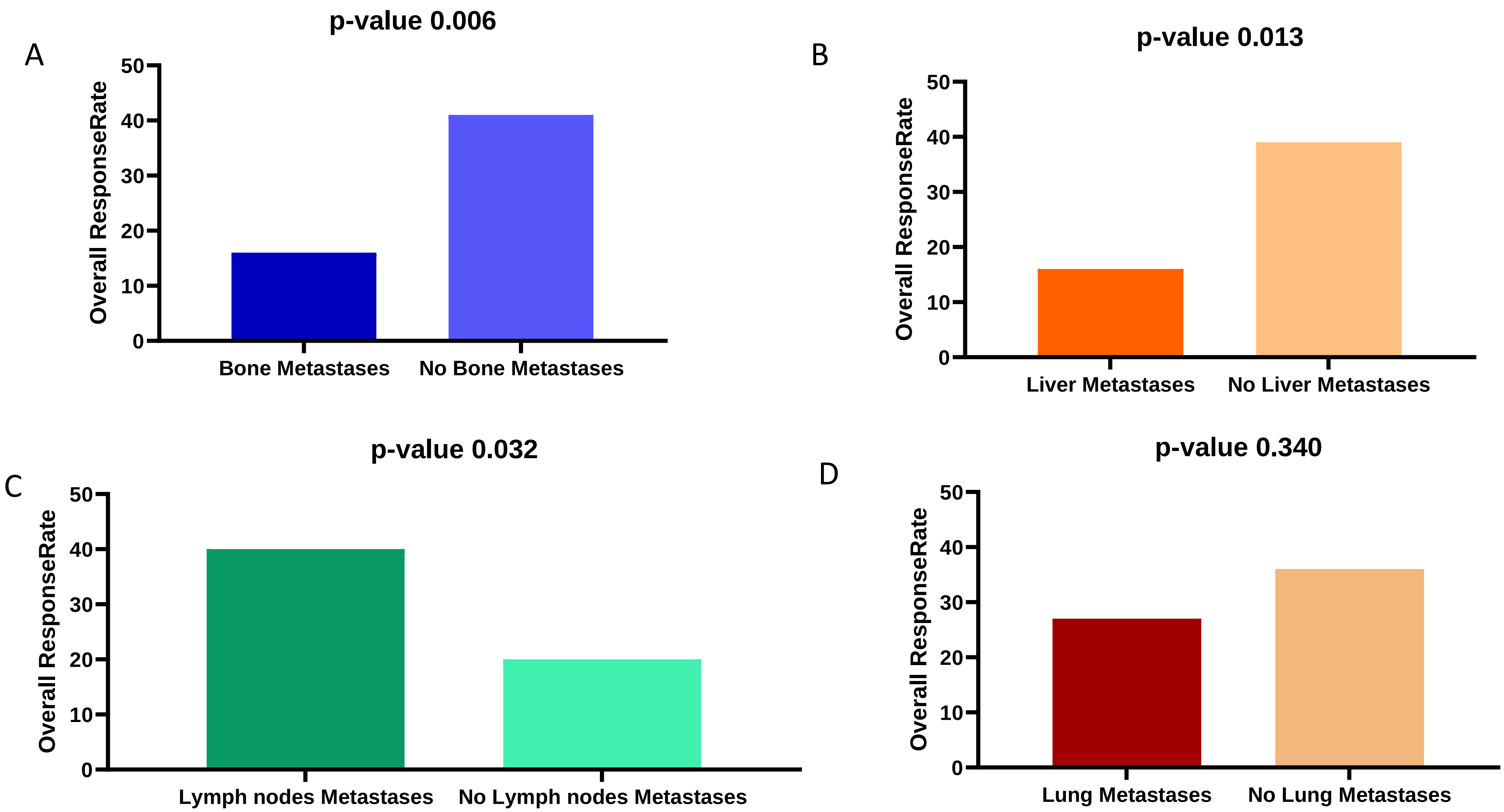

Figure 2. Overall response rate according to metastatic sites

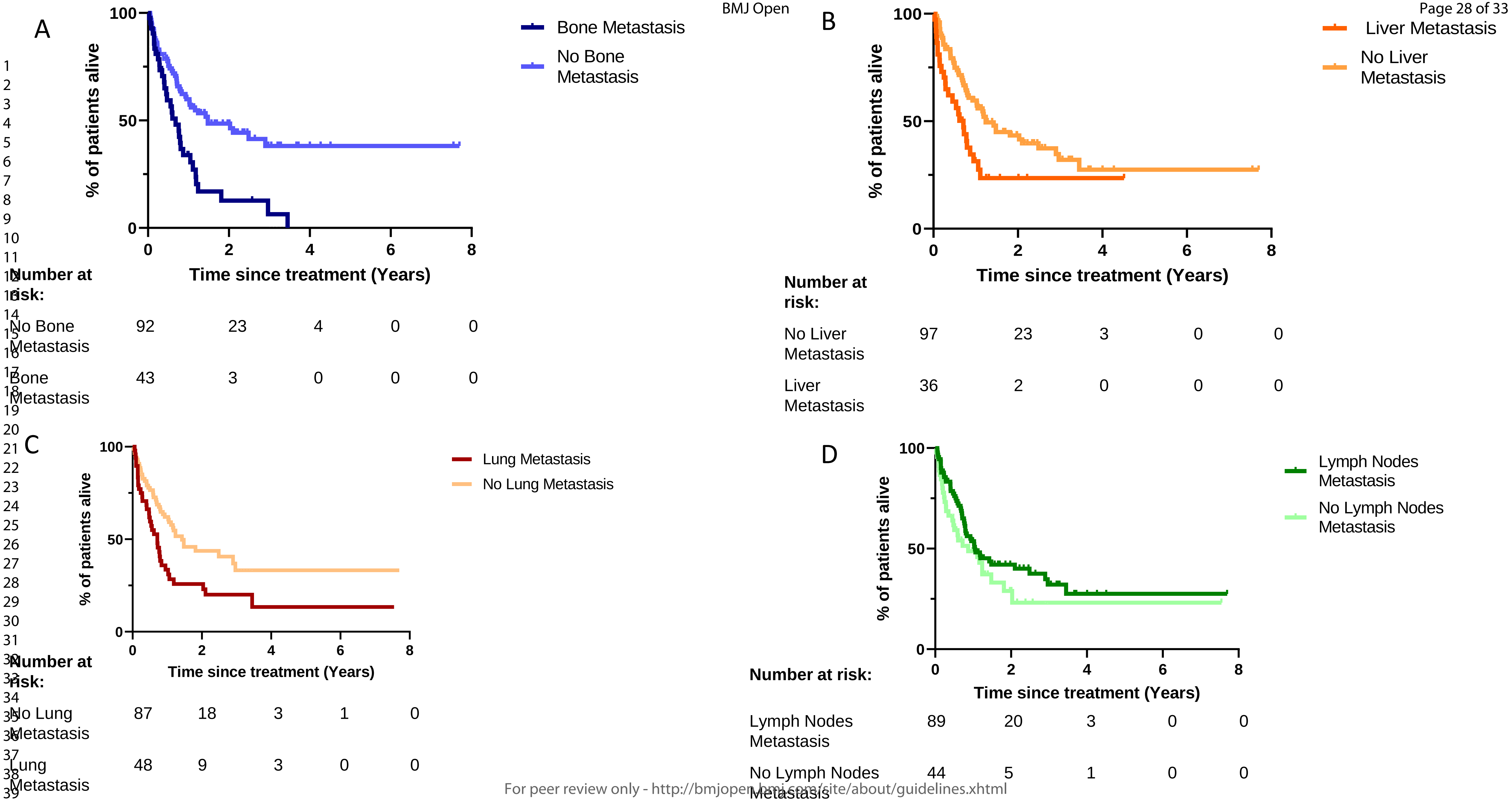

For peer review only - http://bmjopen.bmj.com/site/about/guidelines.xhtml

Figure 3 Overall survival according to metastatic sites

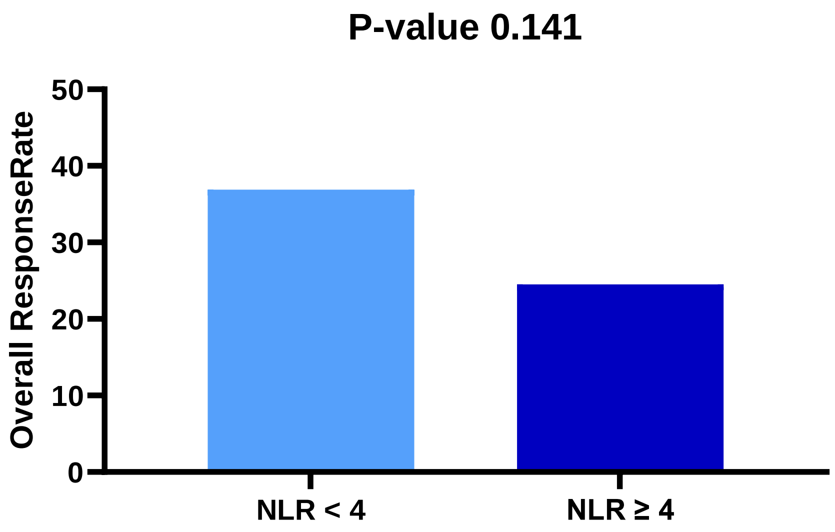

Supplemental Figure 1 Overall response rate according to neutrophil-to-lymphocytes ratio (NLR)

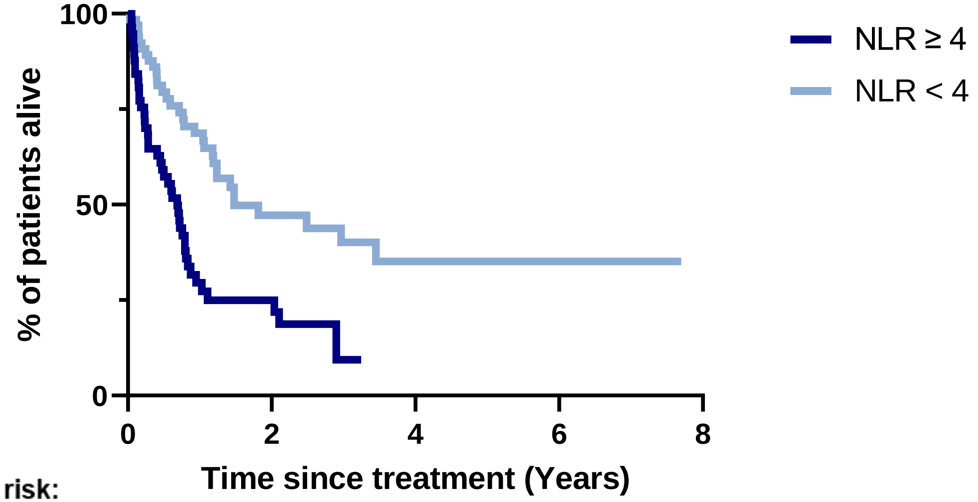

Number at risk:

NLR < 4	65	16	5	2	0
NLR ≥ 4	57	8	0	0	0

Supplemental Figure 2 Overall survival according to NLR

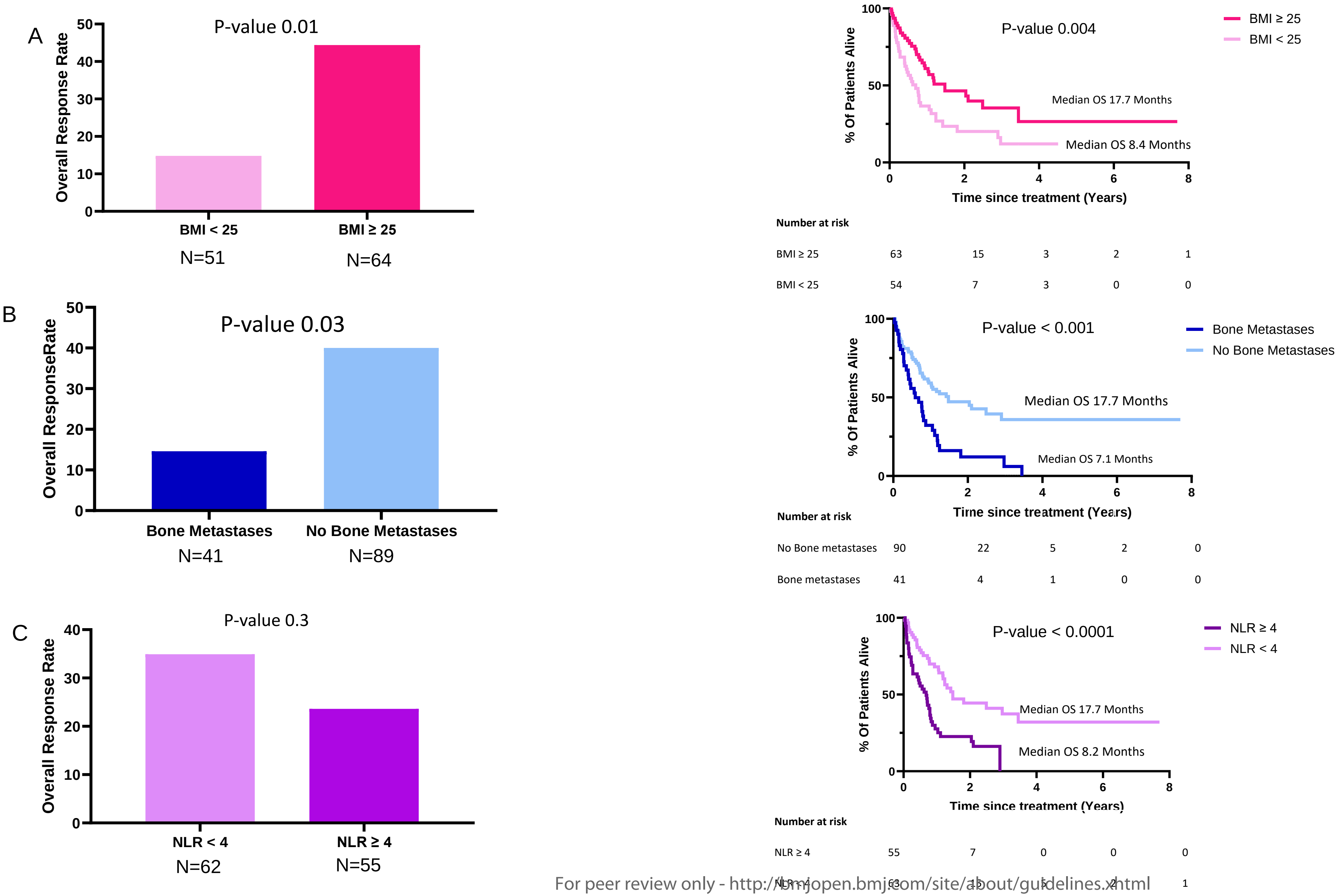

Supplemental Figure 3 ORR and OS according to BMI (A) Bone metastases (B) and NLR (C) in the cohort of patients treated with IO alone

	BMI[a] ≥ 25	BMI[a]< 25	Lymphnode	Lung	Bone	Liver
ORR[b] (CR[c]+PR[d]), %	45.4	16.3	40	27	16	16
CR, n (%)	8 (12.1)	1 (1.8)	7 (7.77%)	2 (4.16%)	1 (2.1%)	1 (2.5%)
PR, n (%)	22 (33.3)	8 (14.5)	29 (32.2%)	11 (22.9%)	6 (13.9%)	5 (13.5%)
SD[e], n (%)	7 (10.6)	10 (18.1)	10 (11.1%)	3 (6.25%)	6 (13.9%)	3 (8.1%)
PD[f], n (%)	24 (43.9)	36 (65.4)	44 (48.8%)	32 (66.6%)	30 (69.7%)	28 (75.6%)

[a]Body mass index [b]Overall response rate [c]Complete response [d]Partial response [e]Stable disease [f]Progressive disease

Supplemental Table: Responses according to clinical variables [a]BMI and metastatic sites

For peer review only

STROBE Statement—Checklist of items that should be included in reports of ***cohort studies***

	Item No	Recommendation
**Title and abstract**	1	(*a*) Indicate the study's design with a commonly used term in the title or the abstract
	✓	(*b*) Provide in the abstract an informative and balanced summary of what was done and what was found
**Introduction**		Abstract - Page 2
Background/rationale ✓	2	Explain the scientific background and rationale for the investigation being reported
Objectives ✓	3	State specific objectives, including any prespecified hypotheses
**Methods**		Page 3-4
Study design ✓	4	Present key elements of study design early in the paper Page 5
Setting ✓	5	Describe the setting, locations, and relevant dates, including periods of recruitment, exposure, follow-up, and data collection Page 5
Participants ✓	6	(*a*) Give the eligibility criteria, and the sources and methods of selection of participants. Describe methods of follow-up Page 5
		(*b*) For matched studies, give matching criteria and number of exposed and unexposed N/A
Variables ✓	7	Clearly define all outcomes, exposures, predictors, potential confounders, and effect modifiers. Give diagnostic criteria, if applicable Page 5
Data sources/ measurement ✓	8*	For each variable of interest, give sources of data and details of methods of assessment (measurement). Describe comparability of assessment methods if there is more than one group Page 5
Bias ✓	9	Describe any efforts to address potential sources of bias Page 13
Study size ✓	10	Explain how the study size was arrived at Page 5
Quantitative variables ✓	11	Explain how quantitative variables were handled in the analyses. If applicable, describe which groupings were chosen and why Page 5
Statistical methods ✓	12	(*a*) Describe all statistical methods, including those used to control for confounding
		(*b*) Describe any methods used to examine subgroups and interactions Page 5 & 6
		(*c*) Explain how missing data were addressed
		(*d*) If applicable, explain how loss to follow-up was addressed
		(*e*) Describe any sensitivity analyses
**Results**		
Participants ✓	13*	(a) Report numbers of individuals at each stage of study—eg numbers potentially eligible, examined for eligibility, confirmed eligible, included in the study, completing follow-up, and analysed Page 5
		(b) Give reasons for non-participation at each stage
		(c) Consider use of a flow diagram
Descriptive data ✓	14*	(a) Give characteristics of study participants (eg demographic, clinical, social) and information on exposures and potential confounders Page 6
		(b) Indicate number of participants with missing data for each variable of interest Page 18
		(c) Summarise follow-up time (eg, average and total amount) Page 6
Outcome data ✓	15*	Report numbers of outcome events or summary measures over time Page 6-7
Main results ✓	16	(*a*) Give unadjusted estimates and, if applicable, confounder-adjusted estimates and their precision (eg, 95% confidence interval). Make clear which confounders were adjusted for and why they were included Page 19
		(*b*) Report category boundaries when continuous variables were categorized Page 19
		(*c*) If relevant, consider translating estimates of relative risk into absolute risk for a meaningful time period N/A

For peer review only

Other analyses	✓	17	Report other analyses done—eg analyses of subgroups and interactions, and sensitivity analyses Page 6-7
**Discussion**			
Key results	✓	18	Summarise key results with reference to study objectives Page 8-12
Limitations	✓	19	Discuss limitations of the study, taking into account sources of potential bias or imprecision. Discuss both direction and magnitude of any potential bias Page 13
Interpretation	✓	20	Give a cautious overall interpretation of results considering objectives, limitations, multiplicity of analyses, results from similar studies, and other relevant evidence Page 8-13
Generalisability	✓	21	Discuss the generalisability (external validity) of the study results Page 13
**Other information**			
Funding	✓	22	Give the source of funding and the role of the funders for the present study and, if applicable, for the original study on which the present article is based Page 18

*Give information separately for exposed and unexposed groups.

Note: An Explanation and Elaboration article discusses each checklist item and gives methodological background and published examples of transparent reporting. The STROBE checklist is best used in conjunction with this article (freely available on the Web sites of PLoS Medicine at http://www.plosmedicine.org/, Annals of Internal Medicine at http://www.annals.org/, and Epidemiology at http://www.epidem.com/). Information on the STROBE Initiative is available at http://www.strobe-statement.org.

For peer review only

BMJ Open

Clinical variables associated with immune checkpoint inhibitor outcomes in patients with metastatic urothelial carcinoma: A multicenter retrospective cohort study

Journal:	*BMJ Open*
Manuscript ID	bmjopen-2023-081480.R2
Article Type:	Original research
Date Submitted by the Author:	27-Feb-2024
Complete List of Authors:	Labidi, Soumaya; Jewish General Hospital, Segal Cancer Centre; McGill University<br>Meti, Nicholas; McGill University<br>Barua, Reeta; Toronto East Health Network Michael Garron Hospital<br>Li, Mengqi; Lady Davis Institute for Medical Research; McGill University<br>Riromar, Jamila; The Royal Hospital, National Oncology Center; University Health Network<br>Jiang, Di Maria; University Health Network; University of Toronto<br>Fallah-Rad, Nazanin; University Health Network; University of Toronto<br>Sridhar, Srikala ; Princess Margaret Hospital Cancer Centre, Medical Oncology<br>Del Rincon, Sonia V.; Lady Davis Institute for Medical Research; McGill University, Oncology<br>Pezo, Rossanna C.; Sunnybrook Health Sciences Centre; University of Toronto<br>Ferrario, Cristiano; Jewish General Hospital, Segal Cancer Centre; McGill University<br>Cheng, Susanna; Sunnybrook Health Sciences Centre; University of Toronto<br>Sacher, Adrian; Princess Margaret Hospital Cancer Centre, Medical Oncology<br>Rose, April; Jewish General Hospital, Segal Cancer Centre; McGill University, Oncology
**Primary Subject Heading**:	Oncology
Secondary Subject Heading:	Oncology, Urology
Keywords:	Urological tumours < ONCOLOGY, Adult oncology < ONCOLOGY, Clinical Reasoning, EPIDEMIOLOGY, ONCOLOGY

SCHOLARONE™ Manuscripts

I, the Submitting Author has the right to grant and does grant on behalf of all authors of the Work (as defined in the below author licence), an exclusive licence and/or a non-exclusive licence for contributions from authors who are: i) UK Crown employees; ii) where BMJ has agreed a CC-BY licence shall apply, and/or iii) in accordance with the terms applicable for US Federal Government officers or employees acting as part of their official duties; on a worldwide, perpetual, irrevocable, royalty-free basis to BMJ Publishing Group Ltd ("BMJ") its licensees and where the relevant Journal is co-owned by BMJ to the co-owners of the Journal, to publish the Work in this journal and any other BMJ products and to exploit all rights, as set out in our licence.

The Submitting Author accepts and understands that any supply made under these terms is made by BMJ to the Submitting Author unless you are acting as an employee on behalf of your employer or a postgraduate student of an affiliated institution which is paying any applicable article publishing charge ("APC") for Open Access articles. Where the Submitting Author wishes to make the Work available on an Open Access basis (and intends to pay the relevant APC), the terms of reuse of such Open Access shall be governed by a Creative Commons licence – details of these licences and which Creative Commons licence will apply to this Work are set out in our licence referred to above.

Other than as permitted in any relevant BMJ Author's Self Archiving Policies, I confirm this Work has not been accepted for publication elsewhere, is not being considered for publication elsewhere and does not duplicate material already published. I confirm all authors consent to publication of this Work and authorise the granting of this licence.

For peer review only

Clinical variables associated with immune checkpoint inhibitor outcomes in patients with metastatic urothelial carcinoma: A multicenter retrospective cohort study

Soumaya Labidi[1,2], Nicholas Meti[3], Reeta Barua[4], Mengqi Li[5,6], Jamila Riromar[7], Di Maria Jiang[8], Nazanin Fallah-Rad[8], Srikala S. Sridhar[8], Sonia V. del Rincon[1,5,6], Rossanna C. Pezo[9], Cristiano Ferrario[1,2], Susanna Cheng[9], Adrian G. Sacher[8]*, April A. N. Rose[1,2,5,6]*

1. Gerald Bronfman Department of Oncology, McGill University, Montréal, QC. Canada
2. Segal Cancer Center, Jewish General Hospital. Montréal, QC, Canada
3. St. Mary's Hospital, Montréal, QC. Canada
4. Michael Garron Hospital. Toronto East Health Network. Toronto, ON. Canada
5. Lady Davis Institute, Jewish General Hospital, Montréal, QC, Canada
6. Division of Experimental Medicine, Faculty of Medicine, McGill University, Montréal, QC, Canada
7. Royal Hospital National Oncology Center, Sultanat, Oman
8. Princess Margaret Cancer Center. Toronto, ON, Canada
9. Odette Cancer Center. Sunnybrook Health Sciences Center. Toronto, ON, Canada

*Equal contribution

Corresponding Author:

April Rose, MD, PhD, FRCPC

3755 Chemin de la Côte-Sainte-Catherine

Office E-444

Montreal, QC, Canada H3T 1E2

Phone: (514) 340-8222 xt 27343

Email: april.rose@mcgill.ca

Running head: Immunotherapy outcomes in urothelial cancer

For peer review only - http://bmjopen.bmj.com/site/about/guidelines.xhtml

Abstract

Objectives: Immune checkpoint inhibitors (ICI) are indicated for metastatic urothelial cancer (mUC), but predictive and prognostic factors are lacking. We investigated clinical variables associated with ICI outcomes.

Methods: We performed a multicenter retrospective cohort study of 135 patients who received ICI for mUC, 2016-2021, at 3 Canadian centres. Clinical characteristics, body mass index (BMI), metastatic sites, neutrophil-to-lymphocyte ratio (NLR), response, and survival were abstracted from chart review.

Results: We identified 135 patients and 62% had received ICI as a second line or later treatment for mUC. A BMI ≥ 25 was significantly correlated to a higher overall response rate (ORR) (45.4% vs 16.3%, P = 0.020). Patients with BMI ≥ 30 experienced longer median overall survival (OS) 24.8 vs. 14.4 for 25 ≤ BMI < 30 and 8.5 months for BMI < 25 (P-value = 0.012). The ORR was lower in the presence of bone metastases 16% vs 41% P-value = 0.006, and liver metastases 16% vs 39% P-value = 0.013. Metastatic lymph nodes were correlated with higher ORR 40% vs 20% P = 0.032. The median OS for bone metastases was 7.3 vs 18 months (P-value < 0.001). Patients with liver metastases had a median OS of 8.6 vs 15 months (P-value = 0.006). No difference for lymph nodes metastases (13.5 vs 12.7 months P-value = 0.175). NLR ≥ 4 had worse OS (8.2 vs 17.7 months P-value = 0.0001). In multivariate analysis, BMI ≥ 30, bone metastases and NLR ≥ 4, performance status ≥ 2 and line of ICI ≥ 2 were independent factors for OS.

Conclusions: Our data identified BMI and bone metastases as novel clinical biomarkers that were independently associated with ICI outcomes in mUC. External and prospective validation are warranted.

For peer review only

For peer review only - http://bmjopen.bmj.com/site/about/guidelines.xhtml

Strengths and limitations

- This is a multicenter cohort study, with a large number of patients, treated with ICI for mUC at 3 Canadian institutions.
- We identified reproductible clinical prognostic factors related with response and survival outcomes – including bone metastases for which there is limited data available from prospective studies.
- The retrospective study design may lead to selection bias related to population studied.

For peer review only - http://bmjopen.bmj.com/site/about/guidelines.xhtml

Introduction

Bladder cancer represents the 10th most common cancer in the world, with approximately 550,000 new cases annually, and accounts for 2.1% of all cancer deaths according to GLOBOCAN 2020[1]. There is a male predominance and smoking is the most common risk factor. In most cases, urothelial carcinomas arise from the urothelium of the bladder but can also develop from the upper urinary tract (renal pelvis and ureters). Tumors invading the detrusor muscle, ie., muscle invasive tumors, account with upfront metastatic disease in 25% of the cases [2]. Despite multimodality management for non-metastatic muscle invasive bladder cancer, 50% of these patients will relapse, with distant metastases in most of the cases [3, 4]. Platinum-based chemotherapy remains the standard first line treatment for metastatic disease [3, 4] . Patients who respond or have stable disease following chemotherapy are eligible for subsequent maintenance therapy with the anti-programmed death ligand 1 (PD-L1) immune checkpoint inhibitor, Avelumab [5], while those who fail to respond to chemotherapy subsequently can receive the anti-programmed death 1 (PD-1) immune checkpoint inhibitor, Pembrolizumab [6]. Other treatment options in the platinum-refractory setting include Enfortumab Vedotin and Erdafitinib in patients with susceptible Fibroblast Growth Factor Receptor (FGFR) alterations, and chemotherapy with taxanes (Paclitaxel and Docetaxel) or Vinflunine [7-10]. In the KEYNOTE-045 randomized phase 3 trial, Pembrolizumab showed a significantly longer OS of 10.3 months vs 7.4 months in patients with platinum refractory mUC compared to chemotherapy (Docetaxel, Paclitaxel or Vinflunine) (Hazard ratio HR 0.73; 95%Confidence interval CI, 0.59 to 0.91 P-value = 0.002), with a lower rate of any grade adverse events (60.9% vs 90.2%) [6]. Long term results confirmed an OS benefit and a favorable safety profile [11]. The PD-L1 checkpoint inhibitor Atezolizumab was also associated with durable objective responses in the IMvigpr210 phase 2 trial, with favorable safety profile, [12] but failed to show survival benefit over chemotherapy in the IMvigor211

For peer review only - http://bmjopen.bmj.com/site/about/guidelines.xhtml

phase 3 trial [13]. In phase 1/2 trials CheckMate275 and CheckMate032, Nivolumab, a PD-1 inhibitor, achieved objective responses alone or in combination with Ipilimumab, with a manageable adverse events profile [14, 15]. Despite responses seen with ICI in the platinum-refractory setting, not all patients will derive benefit and there is a lack of valid prognostic and predictive biomarkers.

In this study, we aimed to evaluate if clinical prognostic markers, that have been defined for other cancer types, are relevant to mUC. In fact, several retrospective studies have addressed the relationship between obesity and outcomes in patients receiving ICI. In melanoma, non-small cell lung cancer (NSCLC) and renal cell cancer (RCC), obesity appears to be linked to better progression free survival (PFS) and OS [16-18]. And a recent systematic review of 18 retrospective studies across different cancer types, by Indini at al., also found an association between high BMI and improved ICI outcomes; yet a strong positive correlation could not be concluded due to the heterogeneity of the studies [19]. As such, the relationship between BMI and immunotherapy outcomes in urothelial cancer remains poorly understood.

The tumor immune microenvironment of different metastatic locations may also affect the response to ICI [20, 21]. Retrospective studies reported differences in organ specific responses and organ specific OS with immunotherapy in several types of cancer [22-24].

Neutrophil-to-lymphocyte ratio (NLR) is an available marker of systemic response to inflammation, derived from the absolute counts of neutrophils and lymphocytes on a blood count [25]. It is a well-established poor prognostic factor in several cancers, independently of treatment type [25-27].

The aim of this retrospective analysis was to assess the association between high BMI, site of metastases, NLR and outcome in terms of response and OS in a population of patients with mUC treated with ICI.

For peer review only - http://bmjopen.bmj.com/site/about/guidelines.xhtml

Patients and methods

Patient population, characteristics and outcome

We performed a multi-center retrospective cohort study analysis of mUC patients, from 3 Canadian cancer centers: Segal Cancer Center, Jewish General Hospital (JGH); Princess Margaret Cancer Center, University Health Network (PM-UHN) and Odette Cancer Center, Sunnybrook Health Sciences Center (SHSC). Patients with histologically proven metastatic urothelial cancer, who received at least one dose of anti-PD-1/anti-PD-L1 ICI, between December 2016 and January 2021, were included regardless of gender, age and performance status. ICI at any line of treatment for metastatic disease, alone or in combination with chemotherapy or another ICI, was allowed. Patients' characteristics were abstracted from chart review. The following clinical characteristics were collected: age, gender, smoking status, comorbidities, primary tumor location and histology, type and line of ICI treatment, number and type of previous treatment lines received, and sites of metastases. BMI at diagnosis, prior to ICI treatment and at progression was assessed. We selected 3 groups according to BMI at start of ICI: BMI < 25, $25 \leq$ BMI < 30 and BMI ≥ 30. We collected the following outcomes criteria: ICI ORR and OS. ICI ORR was determined by investigator assessment of radiologic response as per Response evaluation criteria in solid tumors (RECIST) and was the sum of complete (CR) and partial response (PR). OS was calculated from the start of ICI treatment to death or last follow-up.

Ethics approval

Ethics approval for this study was obtained from the Clinical Trials Ontario (CTO Project ID: 2067) after review by the UHN Research Ethics Board (REB) on August 19, 2020, for PM-UHN and SHSC sites. Ethics approval for the JGH site was approved by the Integrated

For peer review only - http://bmjopen.bmj.com/site/about/guidelines.xhtml

University Health And Social Services Centres (CIUSSS) West Central Montreal REB (Project 2022-2888) on October 28, 2022.

Statistical analysis

Fisher's exact test and chi square test were used to assess differences in response rates between the predefined groups. OS was assessed using Kaplan Meier. Log-rank was used to compare groups and Cox regression models were used to perform univariable and multivariable analysis. P-value < 0.05 were considered statistically significant. Clinical variables that were associated with a p-value<0.05 in the univariate analysis were included in the multivariable model. The final multivariable model included all variables with a p-value <0.05 in the multivariate analysis. The survival analysis was performed with IBM SPSS V.23 (IBM Corp, Armonk, New York, USA). Multivariable analyses were performed with Stata Statistical Software/MP Release 17, College Station, TX: StataCorp LLC. The figures were created using the GraphPad Prism version 10.1.2 for Windows, GraphPad Software (www.graphpad.com).

Patient and public involvement

Patients and/or the public were not involved in the design, conduct, reporting and dissemination plans of this research.

Results

Patients' characteristics

We identified 135 patients, who received at least one dose of ICI for mUC. The median age was 70 years (26-91 years). Most of the patients had a primary bladder cancer (n=125, 92.6%). Most patients (n=84, 62%) received ICI as a second line or later treatment for mUC. The median follow-up period was 14.5 months. Patients' characteristics for the entire cohort are shown in **Table 1**.

For peer review only - http://bmjopen.bmj.com/site/about/guidelines.xhtml

BMI data were available for 121 patients. A BMI ≥ 25 was observed in 48.8% of the patients. Median BMI was 20.9, 26.6 and 34.6 in groups BMI < 25, 25 ≤ BMI < 30 and BMI ≥ 30 respectively. At the time ICI treatment was initiated, metastatic sites were found as follows: lymph nodes 66.7% (n=90), lung 35.6% (n=48), bones 31.9% (n=43) and liver 27.4% (n=37).

The relationship between BMI and ICI response and survival outcomes

We observed differences in ORR according to the BMI category of the patient. The ORR was 45.4% in the BMI ≥ 25 group, versus 16.3% in the BMI < 25 group (P-value = 0.020) (**Figure 1a**) In the BMI ≥ 25 and BMI < 25 groups, we observed a higher proportion of CR to ICI (8 CR vs 1, 22 PR vs 8 and 7 SD vs 10) (**Supplemental table**). Patients with BMI ≥ 30 experienced the longest median OS (24.8 months) compared with those with 25 ≤ BMI < 30 (14.4 months) and BMI < 25 (8.5 months) (P-value = 0.012). (**Figure 1b**). BMI≥ 30 remained an independent prognostic variable in multivariable analysis (HR = 0.40; 95% CI 0.17-0.96; P-value=0.040) (**Table 2**). These results were confirmed for patients treated with ICI alone **(Supplemental Figure 3a)**.

Metastatic sites' response and survival outcomes

The ORR for the entire cohort was 32.2%. The ORR was significantly lower in patients with bone metastases 16% vs 41% (P = 0.006) (**Figure 2a, Supplemental Figure 3b**), or liver metastases 16% vs 39% (P-value = 0.013) (**Figure 2b**). Conversely, the presence of metastatic disease involving the lymph nodes was significantly correlated with higher ORR 40% vs 20% (P-value = 0.032) (**Figure 2c**). The difference in ORR for lung metastases was not statistically significant 27% vs 36% (P-value = 0.340) (**Figure 2d**). Detailed responses are listed in **supplemental table**. The median OS was evaluated for the entire cohort, for each metastatic site and for each BMI group. In the entire cohort, median OS was 12.7 months. Bone, liver and lung metastases correlated with significantly shorter survival. The median OS for patients

For peer review only - http://bmjopen.bmj.com/site/about/guidelines.xhtml

with bone metastases was 7.3 months compared to 18 months in the absence of bone metastases (P-value < 0.001) (**Figure 3a, Supplemental Figure 3b**). Patients with liver metastases had a median OS of 8.6 months compared to 15 months (P-value= 0.006) (**Figure 3b**), and 8.7 months compared to 17.3 months for those with lung metastases (P-value = 0.004) (**Figure 3c**). The presence of lymph node metastases was not significantly associated with OS: 13.5 months vs 12.7 months (P-value = 0.175) (**Figure 3d**). Although bone, lung and liver metastases were all associated with significantly worse OS in univariable analyses, bone metastases were the only metastatic site that remained significantly associated with shorter OS in multi-variable analysis (HR = 1.98; 95%CI 1.17-3.35; P-value = 0.010) (**Table 2**).

NLR response and survival outcomes

Next, we evaluated the relationship between the NLR and clinical outcomes with ICI in this cohort. The ORR was not statistically different for patients with NLR ≥ 4 compared to those with NLR < 4, respectively 24.5% and 36.9% (P-value = 0.141) (**Supplemental Figure 1, Supplemental Figure 3c**). However, NLR < 4 was correlated with better OS, with a median of 17.7 months versus 8.2 months for NLR ≥ 4 (P-value < 0.001) (**Supplemental Figure 2, Supplemental Figure 3c**). The survival benefit associated with a low NLR at the time of initiating ICI therapy was independent of other prognostic variable in multivariable analysis (**Table 2**). We assessed whether there was a relationship between NLR and BMI, but we did not observe any significant correlation (data not shown).

Discussion

ICI are approved for a wide-array of cancers based on tumor type (melanoma, NSCLC, urothelial cancer) and/or in a tumor type agnostic manner based on the presence of molecular biomarkers such as high tumor mutation burden [28-30] and microsatellite instability [30-32]. However, in most cases, many patients who are eligible for immunotherapies based on tumor

For peer review only - http://bmjopen.bmj.com/site/about/guidelines.xhtml

type or biomarker status will either not respond to treatment or will eventually become resistant to treatment. In this analysis, we identified several important clinical biomarkers that associate with better (high BMI) or worse (bone metastases, high NLR) outcomes with ICI in patients with mUC. These biomarkers are easily assessed without any additional costly molecular testing. Moreover, ICIs are expensive, potentially toxic and in mUC only 30% of the patients benefit from ICIs. As such, we believe that these data may be clinically useful to guide treatment decisions.

According to the World Health Organization (WHO) obesity, classified as a BMI greater than 30 kg/m^2, is a rising epidemic [33]. Our data showed strong correlation between elevated BMI and improved outcome in terms of response as well as survival. To the best of our knowledge, the present study is the largest to report data of ICI outcomes correlation with BMI in mUC [19, 34, 35]. A favorable prognostic role of high BMI was also reported in RCC, NSCLC and melanoma [17, 34, 36-38]. A pooled post hoc analysis of individual participant data from 4 prospective trials with Atezolizumab in metastatic NSCLC showed significant difference in survival between normal weight, overweight and obese patients with improved OS for patients with obesity and overweight compared to normal weight. [17] In a retrospective multicohort analysis of 1918 metastatic melanoma patients treated with chemotherapy, targeted therapies and ICI, McQuade et al studied association between BMI and outcome, as well as interactions between BMI and gender and type of treatment. Obesity was associated with improved OS and PFS with a benefit restricted to patients treated with targeted therapies and ICI [16].

There is increasing evidence that excess body weight is a modifiable risk factor for several malignancies, including pancreas, kidney, colorectal, postmenopausal breast, ovarian, gallbladder and thyroid cancers [39]. However, obesity is not an established risk factor for bladder cancer and the impact of obesity on cancer survival is less clear. Some studies have reported that obesity is associated with improved survival in some, but not all, patients with

For peer review only - http://bmjopen.bmj.com/site/about/guidelines.xhtml

cancer, which is referred to the "obesity paradox" [40]. Numerous studies have shown that obesity can cause immune cell dysfunction [41-44], while others argue that the chronic low-grade inflammation underpinning obesity creates a pro-inflammatory state that synergizes with immunotherapy [45, 46].

Pre-clinical and translational research has provided insight into how immune checkpoint blockade can benefit obese patients. Obesity appears to influence T cell function and phenotype. For example, leptin-associated T cell dysfunction due to obesity has been observed across different species and tumor models and was found to enhance the immune response to anti-PD-1 therapy [41]. In a mouse study, Shirakawa et al. reported that obesity caused a preferential increase and accumulation of senescent $CD44^{hi}CD62L^{lo}CD4+$ T cells that constitutively express PD-1 and CD153 [47]. Moreover, a study in a colon carcinoma mouse model showed that obesity impaired the infiltration, cytokine production and metabolic activity of tumor-infiltrating CD8 T cells, while anti-PD-1 restored CD8 T cells infiltration, proinflammatory cytokine production and metabolic activity, leading to complete tumor eradication and immune memory [48]. Obesity may also affect other immune cells in the tumor microenvironment (TME), such as myeloid-derived suppressor cells (MDSCs), which are known to suppress T cell responses. MDSCs can suppress T cell activity through many mechanisms, one of which is expressing PD-L1 to induce T cell exhaustion [49, 50]. In a pre-clinical study, inhibiting PD-L1 on MDSCs decreased their ability to suppress T cell activity, suggesting that obesity could also accelerate tumor progression by promoting the expression of PD-L1 on MDSCs [51]. Finally, a preclinical study in a breast cancer model showed that although obesity accelerated tumor progression, anti-PD-1 treatment significantly reduced tumor burden by reshaping the local TME landscape [52].

For peer review only

The relationship between obesity and ICI is complex and depends on various of factors, including the types of cancer and ICI involved. However, BMI may be an imperfect marker and its evaluation based only on weight prior to ICI start can be limiting. Assessment of the weight loss or change over the time may be a better indicator of disease prognosis. As BMI cannot distinguish body fat from muscle, it may not be the best tool to assess obesity [53]. Other measurement methods could be used for better assessment such as dual energy x-ray absorptiometry or fat referenced quantitative MRI [54].

Further studies are needed to understand the molecular mechanisms underlying the interaction between obesity and cancer immunity and to identify potential targets for effective interventions.

Bone metastases are a validated negative prognostic factor in mUC treated with platinum based chemotherapy [55, 56]. In our study, poor response and survival outcome of bone and liver metastases was significant, while lymph nodes metastases correlated with higher ORR. These results are consistent with published data in the literature for patients treated with ICI [57-59]. Makrakis et al. showed lower response rates and shorter OS for bone and liver metastases on retrospective data from 917 mUC treated with ICI as 1st or $\geq 2^{nd}$ line, but higher ORR for lymph node confined metastases [57]. A retrospective multicentric Japanese study reported shorter ORR for bone metastases in a cohort of patients treated with Pembrolizumab for mUC [58]. Similar results have also been reported from prospective trials [60, 61]. In the IMvigor210 phase 2 trial, the ORR on Atezolizumab was 32% for lymph nodes confined metastases, and only 8% for liver metastases, data for bone metastases were not reported [60]. Higher ORR for lymph nodes metastases (47%) compared to liver metastases (23%) was also reported in the Keynote-052 trial [61]. Prognostic models for mUC patients treated with ICI identified liver or visceral metastases as poor prognostic factors [62, 63]. Sonpavde et al. developed a five-factor prognostic model for survival for mUC treated with Atezolizumab, Avelumab or Durvalumab,

For peer review only - http://bmjopen.bmj.com/site/about/guidelines.xhtml

based on the data from phase 1 and 2 trials. [64] They identified 3 risk groups based on performance status, liver metastases, platelet count, NLR and lactate dehydrogenase levels (LDH) as prognostic factors. [64] The 1-year Kaplan-Meier survival estimates of those in the low, intermediate and poor risk groups were 76.2%, 33.8 % and 8.6%, respectively. [64] The authors developed an interactive web-based tool to calculate the expected survival probabilities according to the five-factors. [64] In our study, LDH levels were not uniformly assessed prior to ICI treatment at all sites, and we did not collect data for platelet count. Therefore, we could not compare the prognostic utility of this tool in our cohort of patients. Most prospective trials of ICI in mUC do not use presence/absence of bone metastases as a stratification factor, despite the fact that bone metastases are associated with poor response to chemotherapy and impact quality of life [55, 56, 65]. Owari et al validated a specific prognostic scoring system, B-FOM, to predict survival for patients with bone metastasis from different genito-urinary cancers based on 5 prognostic factors: primary tumor (prostate, renal or urothelial cancers), poor performance status, visceral metastases, high Glasgow-prognostic score and elevated NLR [66]. This prediction tool may be helpful to individualize optimal treatment strategy for the patients. A better understanding of the bone microenvironment is crucial to help the development of novel therapeutic strategies and improve outcomes [67].

The distant organ microenvironment, also known as metastatic microenvironment (MME), plays an important role in site-specific metastases [68]. For instance, macrophages, which are abundant immune cells in the lungs, can bind to cancer cells via receptor VCAM-1 transmit, signaling a chain of events leading to lung-specific metastases in breast cancer [69]. It has been demonstrated that myeloid cells can remodel the pre-metastatic lung from an immune protective state to a state favoring tumor progression, thereby promoting lung metastases [70]. Furthermore, lung stromal cells can also promote tumor colonization and metastasis by secreting periostin [71]. In the liver, there is a significant presence of circulating and resident

For peer review only - http://bmjopen.bmj.com/site/about/guidelines.xhtml

natural killer (NK) cells, which serve as the primary effectors of liver immune function. This NK cell maintained the breast cancer cells dormant in the liver by secreting Interferonγ (IFNγ), while sustaining NK cell abundance with Interleukine15 (IL15) based immunotherapy succeeded to prevent liver metastases and prolong the survival in preclinical models [72]. Conversely, bone forms an immunosuppressive environment mainly due to immature NK cells, a small number of cytotoxic T cells, a large number of myeloid progenitors and Treg [73]. In a human bladder tumor xenografts model, researchers observed a high infiltration of bone marrow-derived host CD11b myeloid cells. They further demonstrated that enhanced tumor-associated in the TME promote an immunosuppressive pro-tumoral myeloid phenotype [74]. These observations demonstrate how the MME can influence the metastatic potential and outcome of different types of cancer cells in different organs, suggesting that MME might also potentiate site-specific metastases in bladder cancer. Taken together, all these studies suggest the importance of understanding the metastatic potential and outcome of different types of cancer cells in different organs, through which we can further develop more effective therapeutic measures against bladder cancer.

NLR is a well-established poor prognostic factor in several cancers, independently of treatment type [25-27]. It could be considered as a surrogate marker of chronic inflammation and evasion of immune surveillance [75]. In our study, high NLR was associated with shorter median OS. A recent study by Valero et al showed poor response and survival outcomes, for 1917 patients with high NLR at diagnosis, treated with ICI for multiple cancer types [75]. Similar results were reported for melanoma and NSCLC [26, 76]. Banna et al explored the prognostic and predictive role of NLR and LDH in metastatic urothelial cancers treated with ICI and showed significant correlation with PFS and OS for high NLR [77]. For patients treated with ICI for mUC, a high NLR prior to first line chemotherapy and second line pembrolizumab was

For peer review only - http://bmjopen.bmj.com/site/about/guidelines.xhtml

associated with worse survival outcomes [78]. NLR may represent an accessible low-cost predictive biomarker.

The present study has some strengths and limitations. It presents a relatively large multi-center cohort of patients with significant prognostic factors. On the other hand, this is a retrospective study, with heterogenous population and an investigator-based response assessment on clinical and radiological chart review. However, the observations made in this analysis are hypothesis generating. The evaluation of obesity and its relationship with ICI response warrants future prospective studies that incorporate better established measures of obesity and metabolic syndrome. Moreover, little is known about how bladder cancer cells interact with the liver and bone TME and this warrants further investigation in preclinical models to develop optimal immunotherapies for patients with bone and liver mUC.

Conclusion

Our study identified elevated BMI, NLR and presence of bone metastases as potential biomarkers for ICI response and survival in mUC. Obesity was associated with improved survival and response rate, whereas bone metastases and high NLR are associated with lack of response and shorter survival. Prospective validation of these data is warranted, especially in the evolving landscape of therapeutic options for mUC with novel agents and combinations with ICI [79].

For peer review only

For peer review only - http://bmjopen.bmj.com/site/about/guidelines.xhtml

Contributorship statement

Guarantor of integrity of the entire study: April A.N Rose, Soumaya Labidi

Study concepts and design: Adrian G. Sacher, April A. N. Rose

Literature research: Soumaya Labidi, Mengqi Li

Data collection: Soumaya Labidi, Nicholas Meti, Reeta Barua

Statistical analysis: Soumaya Labidi, April A.N Rose

Manuscript preparation: Soumaya Labidi, Mengqi Li, April A.N Rose

Manuscript editing and critical analysis: Nicholas Meti, Di Maria Jiang, Srikala S. Sridhar, Sonia V. del Rincon, April A. N. Rose

All authors were involved in data interpretation, critical appraisal of the draft manuscript and gave final approval on the submitted version.

Competing interests

Nicholas Meti: Honoraria: Takeda, Novartis. Consulting: Takeda, Seagen, AstraZeneca, Pfizer

Srikala S. Sridhar: Consulting: Astellas, AstraZeneca, Bayer, Bicycle Therapeutics, BMS, Eisai, EMD Serono, Gilead, Ipsen, Janssen, Merck, Pfizer, Seagen
Research funding (Institution): Janssen, Seagen, EMD Serono

Rossanna C. Pezo: Honoraria: Pfizer, Novartis, BMS, Merck, Sanofi, Gilead, AstraZeneca
Research funding (Institution): Merck, Novartis, Pfizer, Taiho, Jazz Pharmaceuticals, ZymeWorks, AstraZeneca

Cristiano Ferrario: Honoraria: Pfizer, Bayer, Novartis, Roche, Bayer, Knight therapeutics, AstraZeneca, Merck, Astellas Pharma. Consulting: Merck, Novartis, AstraZeneca, Roche. Research funding (Institution): Merck, Novartis, Pfizer, Lilly, ZymeWorks, AstraZeneca, Janssen Oncology, Bayer, Astellas Pharma, Roche, Sanofi, Seattle Genetics, Semonix Pharmaceuticals, Bicycle Therapeutics, Immunomedics

Susanna Cheng: Consulting: AstraZeneca, Merck

April A.N. Rose: Consulting: EMD Serono, Advanced accelerator applications/Novartis. Research funding (Personal): Canadian Institutes of Health Research CIHR, Canadian Cancer Society, Conquer Cancer Foundation, Jewish General Hospital Foundation, TransMedTech Institute, Canada Foundation of Innovation. Research Funding (Institution): AstraZeneca, Merck, Pfizer, Seattle Genetics. Employment: Family member Merck

Soumaya Labidi, Mengqi Li, Sonia V. Del Rincon, Reeta Barua, Jamila Riromar, Di Maria Jiang, Nazanin Fallah-Rad, Adrian G. Sacher: No competing interests

Funding

April A.N. Rose acknowledges support from a Fonds de Recherche du Québec – Santé (FRQS) Clinical Research Scholar Award. This research was supported by a University of Toronto, Medical Oncology Strategic Planning Medical Oncology Innovation Fund to Adrian G. Sacher

For peer review only - http://bmjopen.bmj.com/site/about/guidelines.xhtml

and April A.N. Rose and funding from the Jewish General Hospital (JGH) Foundation to April A.N. Rose.

Data sharing statement

All data relevant to the study are included in the article or uploaded as supplementary information. The raw data can not be shared.

For peer review only

For peer review only - http://bmjopen.bmj.com/site/about/guidelines.xhtml

REFERENCES

1. Sung, H., et al., *Global Cancer Statistics 2020: GLOBOCAN Estimates of Incidence and Mortality Worldwide for 36 Cancers in 185 Countries.* CA Cancer J Clin, 2021. **71**(3): p. 209-249.
2. Humphrey, P.A., et al., *The 2016 WHO Classification of Tumours of the Urinary System and Male Genital Organs—Part B: Prostate and Bladder Tumours.* European Urology, 2016. **70**(1): p. 106-119.
3. Nadal, R. and J. Bellmunt, *Management of metastatic bladder cancer.* Cancer Treatment Reviews, 2019. **76**: p. 10-21.
4. Witjes, J.A., et al., *European Association of Urology Guidelines on Muscle-invasive and Metastatic Bladder Cancer: Summary of the 2020 Guidelines.* Eur Urol, 2021. **79**(1): p. 82-104.
5. Powles, T., et al., *Avelumab Maintenance Therapy for Advanced or Metastatic Urothelial Carcinoma.* N Engl J Med, 2020. **383**(13): p. 1218-1230.
6. Bellmunt, J., et al., *Pembrolizumab as Second-Line Therapy for Advanced Urothelial Carcinoma.* N Engl J Med, 2017. **376**(11): p. 1015-1026.
7. McCaffrey, J.A., et al., *Phase II trial of docetaxel in patients with advanced or metastatic transitional-cell carcinoma.* J Clin Oncol, 1997. **15**(5): p. 1853-7.
8. Dreicer, R., et al., *Paclitaxel in advanced urothelial carcinoma: its role in patients with renal insufficiency and as salvage therapy.* J Urol, 1996. **156**(5): p. 1606-8.
9. Ko, Y.J., et al., *Nanoparticle albumin-bound paclitaxel for second-line treatment of metastatic urothelial carcinoma: a single group, multicentre, phase 2 study.* Lancet Oncol, 2013. **14**(8): p. 769-76.
10. Bellmunt, J., et al., *Long-term survival results of a randomized phase III trial of vinflunine plus best supportive care versus best supportive care alone in advanced urothelial carcinoma patients after failure of platinum-based chemotherapy.* Ann Oncol, 2013. **24**(6): p. 1466-72.
11. Fradet, Y., et al., *Randomized phase III KEYNOTE-045 trial of pembrolizumab versus paclitaxel, docetaxel, or vinflunine in recurrent advanced urothelial cancer: results of >2 years of follow-up.* Ann Oncol, 2019. **30**(6): p. 970-976.
12. Rosenberg, J.E., et al., *Atezolizumab in patients with locally advanced and metastatic urothelial carcinoma who have progressed following treatment with platinum-based chemotherapy: a single-arm, multicentre, phase 2 trial.* Lancet, 2016. **387**(10031): p. 1909-20.
13. Powles, T., et al., *Atezolizumab versus chemotherapy in patients with platinum-treated locally advanced or metastatic urothelial carcinoma (IMvigor211): a multicentre, open-label, phase 3 randomised controlled trial.* Lancet, 2018. **391**(10122): p. 748-757.
14. Sharma, P., et al., *Nivolumab in metastatic urothelial carcinoma after platinum therapy (CheckMate 275): a multicentre, single-arm, phase 2 trial.* Lancet Oncol, 2017. **18**(3): p. 312-322.
15. Sharma, P., et al., *Nivolumab Alone and With Ipilimumab in Previously Treated Metastatic Urothelial Carcinoma: CheckMate 032 Nivolumab 1 mg/kg Plus Ipilimumab 3 mg/kg Expansion Cohort Results.* J Clin Oncol, 2019. **37**(19): p. 1608-1616.
16. McQuade, J.L., et al., *Association of body-mass index and outcomes in patients with metastatic melanoma treated with targeted therapy, immunotherapy, or chemotherapy: a retrospective, multicohort analysis.* Lancet Oncol, 2018. **19**(3): p. 310-322.
17. Kichenadasse, G., et al., *Association Between Body Mass Index and Overall Survival With Immune Checkpoint Inhibitor Therapy for Advanced Non-Small Cell Lung Cancer.* JAMA Oncol, 2020. **6**(4): p. 512-518.
18. Albiges, L., et al., *Body Mass Index and Metastatic Renal Cell Carcinoma: Clinical and Biological Correlations.* J Clin Oncol, 2016. **34**(30): p. 3655-3663.

For peer review only - http://bmjopen.bmj.com/site/about/guidelines.xhtml

19. Indini, A., et al., *Impact of BMI on Survival Outcomes of Immunotherapy in Solid Tumors: A Systematic Review.* Int J Mol Sci, 2021. **22**(5).
20. Ganesh, K. and J. Massague, *Targeting metastatic cancer.* Nat Med, 2021. **27**(1): p. 34-44.
21. Oliver, A.J., et al., *Tissue-Dependent Tumor Microenvironments and Their Impact on Immunotherapy Responses.* Front Immunol, 2018. **9**: p. 70.
22. Furubayashi, N., et al., *Organ-Specific Tumor Response to Pembrolizumab in Advanced Urothelial Carcinoma After Platinum-Based Chemotherapy.* Onco Targets Ther, 2021. **14**: p. 1981-1988.
23. Tumeh, P.C., et al., *Liver Metastasis and Treatment Outcome with Anti-PD-1 Monoclonal Antibody in Patients with Melanoma and NSCLC.* Cancer Immunol Res, 2017. **5**(5): p. 417-424.
24. Schmid, S., et al., *Organ-specific response to nivolumab in patients with non-small cell lung cancer (NSCLC).* Cancer Immunol Immunother, 2018. **67**(12): p. 1825-1832.
25. Guthrie, G.J., et al., *The systemic inflammation-based neutrophil-lymphocyte ratio: experience in patients with cancer.* Crit Rev Oncol Hematol, 2013. **88**(1): p. 218-30.
26. Cedres, S., et al., *Neutrophil to lymphocyte ratio (NLR) as an indicator of poor prognosis in stage IV non-small cell lung cancer.* Clin Transl Oncol, 2012. **14**(11): p. 864-9.
27. Templeton, A.J., et al., *Prognostic role of neutrophil-to-lymphocyte ratio in solid tumors: a systematic review and meta-analysis.* J Natl Cancer Inst, 2014. **106**(6): p. dju124.
28. Kim, S.I., C.R. Cassella, and K.T. Byrne, *Tumor Burden and Immunotherapy: Impact on Immune Infiltration and Therapeutic Outcomes.* Front Immunol, 2020. **11**: p. 629722.
29. Samstein, R.M., et al., *Tumor mutational load predicts survival after immunotherapy across multiple cancer types.* Nat Genet, 2019. **51**(2): p. 202-206.
30. Palmeri, M., et al., *Real-world application of tumor mutational burden-high (TMB-high) and microsatellite instability (MSI) confirms their utility as immunotherapy biomarkers.* ESMO Open, 2022. **7**(1): p. 100336.
31. O'Malley, D.M., et al., *Pembrolizumab in Patients With Microsatellite Instability-High Advanced Endometrial Cancer: Results From the KEYNOTE-158 Study.* J Clin Oncol, 2022. **40**(7): p. 752-761.
32. Maio, M., et al., *Pembrolizumab in microsatellite instability high or mismatch repair deficient cancers: updated analysis from the phase II KEYNOTE-158 study.* Ann Oncol, 2022. **33**(9): p. 929-938.
33. Jensen, M.D., et al., *2013 AHA/ACC/TOS guideline for the management of overweight and obesity in adults: a report of the American College of Cardiology/American Heart Association Task Force on Practice Guidelines and The Obesity Society.* Circulation, 2014. **129**(25 Suppl 2): p. S102-38.
34. Cortellini, A., et al., *A multicenter study of body mass index in cancer patients treated with anti-PD-1/PD-L1 immune checkpoint inhibitors: when overweight becomes favorable.* Journal for ImmunoTherapy of Cancer, 2019. **7**(1).
35. Yoo, S.K., et al., *Outcomes Among Patients With or Without Obesity and With Cancer Following Treatment With Immune Checkpoint Blockade.* JAMA Netw Open, 2022. **5**(2): p. e220448.
36. Martini, D.J., et al., *Adiposity may predict survival in patients with advanced stage cancer treated with immunotherapy in phase 1 clinical trials.* Cancer, 2020. **126**(3): p. 575-582.
37. Rutkowski, P., et al., *Body mass index (BMI) and outcome of metastatic melanoma patients receiving targeted therapy and immunotherapy: a multicenter international retrospective study.* Journal for ImmunoTherapy of Cancer, 2020. **8**(2): p. e001117.
38. Richtig, G., et al., *Body mass index may predict the response to ipilimumab in metastatic melanoma: An observational multi-centre study.* PLOS ONE, 2018. **13**(10): p. e0204729.
39. Lauby-Secretan, B., et al., *Body fatness and cancer—viewpoint of the IARC Working Group.* New England journal of medicine, 2016. **375**(8): p. 794-798.

For peer review only - http://bmjopen.bmj.com/site/about/guidelines.xhtml

40. Lennon, H., et al., *The obesity paradox in cancer: a review.* Current oncology reports, 2016. **18**: p. 1-8.
41. Wang, Z., et al., *Paradoxical effects of obesity on T cell function during tumor progression and PD-1 checkpoint blockade.* Nature medicine, 2019. **25**(1): p. 141-151.
42. Rebeles, J., et al., *Obesity-induced changes in T-cell metabolism are associated with impaired memory T-cell response to influenza and are not reversed with weight loss.* The Journal of Infectious Diseases, 2019. **219**(10): p. 1652-1661.
43. Gibson, J.T., et al., *Obesity-associated myeloid-derived suppressor cells promote apoptosis of tumor-infiltrating CD8 T cells and immunotherapy resistance in breast cancer.* Frontiers in immunology, 2020. **11**: p. 590794.
44. Ringel, A.E., et al., *Obesity shapes metabolism in the tumor microenvironment to suppress anti-tumor immunity.* Cell, 2020. **183**(7): p. 1848-1866. e26.
45. Wang, Z., A.M. Monjazeb, and W.J. Murphy, *The complicated effects of obesity on cancer and immunotherapy*. 2019, Future Medicine. p. 11-14.
46. Fang, H., et al., *Regulating the Obesity-Related Tumor Microenvironment to Improve Cancer Immunotherapy.* ACS nano, 2023. **17**(5): p. 4748-4763.
47. Shirakawa, K., et al., *Obesity accelerates T cell senescence in murine visceral adipose tissue.* The Journal of clinical investigation, 2016. **126**(12): p. 4626-4639.
48. Dyck, L., et al., *Suppressive effects of the obese tumor microenvironment on CD8 T cell infiltration and effector function.* Journal of Experimental Medicine, 2022. **219**(3).
49. Lau, J., et al., *Tumour and host cell PD-L1 is required to mediate suppression of anti-tumour immunity in mice.* Nature communications, 2017. **8**(1): p. 14572.
50. Weber, R., et al., *Myeloid-derived suppressor cells hinder the anti-cancer activity of immune checkpoint inhibitors.* Frontiers in immunology, 2018. **9**: p. 1310.
51. Hale, M., et al., *Obesity triggers enhanced MDSC accumulation in murine renal tumors via elevated local production of CCL2.* PloS one, 2015. **10**(3): p. e0118784.
52. Pingili, A.K., et al., *Immune checkpoint blockade reprograms systemic immune landscape and tumor microenvironment in obesity-associated breast cancer.* Cell reports, 2021. **35**(12): p. 109285.
53. Arnett, D.K., et al., *2019 ACC/AHA Guideline on the Primary Prevention of Cardiovascular Disease: A Report of the American College of Cardiology/American Heart Association Task Force on Clinical Practice Guidelines.* J Am Coll Cardiol, 2019. **74**(10): p. e177-e232.
54. Borga, M., et al., *Advanced body composition assessment: from body mass index to body composition profiling.* J Investig Med, 2018. **66**(5): p. 1-9.
55. Alqaisi, H.A., et al., *The prognostic impact of bone metastasis in patients with metastatic urothelial carcinoma treated with first-line platinum-based chemotherapy.* Ther Adv Med Oncol, 2022. **14**: p. 17588359221094879.
56. Necchi, A., et al., *Nomogram-based Prediction of Overall Survival in Patients with Metastatic Urothelial Carcinoma Receiving First-line Platinum-based Chemotherapy: Retrospective International Study of Invasive/Advanced Cancer of the Urothelium (RISC).* Eur Urol, 2017. **71**(2): p. 281-289.
57. Makrakis, D., et al., *Association Between Sites of Metastasis and Outcomes With Immune Checkpoint Inhibitors in Advanced Urothelial Carcinoma.* Clin Genitourin Cancer, 2022. **20**(5): p. e440-e452.
58. Shimizu, T., et al., *Organ-Specific and Mixed Responses to Pembrolizumab in Patients with Unresectable or Metastatic Urothelial Carcinoma: A Multicenter Retrospective Study.* Cancers (Basel), 2022. **14**(7).
59. Raggi, D., et al., *Role of Bone Metastases in Patients Receiving Immunotherapy for Pre-Treated Urothelial Carcinoma: The Multicentre, Retrospective Meet-URO-1 Bone Study.* Clin Genitourin Cancer, 2022. **20**(2): p. 155-164.

For peer review only - http://bmjopen.bmj.com/site/about/guidelines.xhtml

60. Balar, A.V., et al., *Atezolizumab as first-line treatment in cisplatin-ineligible patients with locally advanced and metastatic urothelial carcinoma: a single-arm, multicentre, phase 2 trial.* Lancet, 2017. **389**(10064): p. 67-76.
61. Balar, A.V., et al., *First-line pembrolizumab in cisplatin-ineligible patients with locally advanced and unresectable or metastatic urothelial cancer (KEYNOTE-052): a multicentre, single-arm, phase 2 study.* Lancet Oncol, 2017. **18**(11): p. 1483-1492.
62. Nassar, A.H., et al., *A model combining clinical and genomic factors to predict response to PD-1/PD-L1 blockade in advanced urothelial carcinoma.* Br J Cancer, 2020. **122**(4): p. 555-563.
63. Khaki, A.R., et al., *A New Prognostic Model in Patients with Advanced Urothelial Carcinoma Treated with First-line Immune Checkpoint Inhibitors.* Eur Urol Oncol, 2021. **4**(3): p. 464-472.
64. Sonpavde, G., et al., *Five-Factor Prognostic Model for Survival of Post-Platinum Patients with Metastatic Urothelial Carcinoma Receiving PD-L1 Inhibitors.* J Urol, 2020. **204**(6): p. 1173-1179.
65. Stellato, M., et al., *Bone metastases from urothelial carcinoma. The dark side of the moon.* J Bone Oncol, 2021. **31**: p. 100405.
66. Owari, T., et al., *External validation of a genitourinary cancer-specific prognostic scoring system to predict survival for patients with bone metastasis (modified B-FOM scoring model): Comparison with other scoring models in terms of accuracy.* J Bone Oncol, 2021. **26**: p. 100344.
67. Reinstein, Z.Z., et al., *Overcoming immunosuppression in bone metastases.* Crit Rev Oncol Hematol, 2017. **117**: p. 114-127.
68. Liu, Q., et al., *Factors involved in cancer metastasis: a better understanding to "seed and soil" hypothesis.* Molecular Cancer, 2017. **16**(1): p. 176.
69. Chen, Q., X.H.-F. Zhang, and J. Massagué, *Macrophage binding to receptor VCAM-1 transmits survival signals in breast cancer cells that invade the lungs.* Cancer cell, 2011. **20**(4): p. 538-549.
70. Yan, H.H., et al., *Gr-1+ CD11b+ myeloid cells tip the balance of immune protection to tumor promotion in the premetastatic lung.* Cancer research, 2010. **70**(15): p. 6139-6149.
71. Malanchi, I., et al., *Interactions between cancer stem cells and their niche govern metastatic colonization.* Nature, 2012. **481**(7379): p. 85-89.
72. Correia, A.L., et al., *Hepatic stellate cells suppress NK cell-sustained breast cancer dormancy.* Nature, 2021. **594**(7864): p. 566-571.
73. Zhao, E., et al., *Bone marrow and the control of immunity.* Cellular & molecular immunology, 2012. **9**(1): p. 11-19.
74. Eruslanov, E., et al., *Aberrant PGE2 metabolism in bladder tumor microenvironment promotes immunosuppressive phenotype of tumor-infiltrating myeloid cells.* International immunopharmacology, 2011. **11**(7): p. 848-855.
75. Valero, C., et al., *Pretreatment neutrophil-to-lymphocyte ratio and mutational burden as biomarkers of tumor response to immune checkpoint inhibitors.* Nat Commun, 2021. **12**(1): p. 729.
76. Capone, M., et al., *Baseline neutrophil-to-lymphocyte ratio (NLR) and derived NLR could predict overall survival in patients with advanced melanoma treated with nivolumab.* J Immunother Cancer, 2018. **6**(1): p. 74.
77. Banna, G.L., et al., *Neutrophil-to-lymphocyte ratio and lactate dehydrogenase as biomarkers for urothelial cancer treated with immunotherapy.* Clin Transl Oncol, 2020. **22**(11): p. 2130-2135.
78. Kobayashi, T., et al., *Pre-pembrolizumab neutrophil-to-lymphocyte ratio (NLR) predicts the efficacy of second-line pembrolizumab treatment in urothelial cancer regardless of the pre-chemo NLR.* Cancer Immunol Immunother, 2022. **71**(2): p. 461-471.

For peer review only - http://bmjopen.bmj.com/site/about/guidelines.xhtml

79. Hoimes, C.J., et al., *Enfortumab Vedotin Plus Pembrolizumab in Previously Untreated Advanced Urothelial Cancer.* J Clin Oncol, 2023. **41**(1): p. 22-31.

For peer review only

For peer review only - http://bmjopen.bmj.com/site/about/guidelines.xhtml

Table 1: Patients' characteristics

Clinical Characteristic	N (%)
Age	
Age ≤ 60 years	28 (20.7%)
Age > 60 years	107 (79.3%)
Gender	
Male	101 (74.8%)
Female	34 (25.2%)
Smoking status	
Ever smoker	69 (51.1%)
Never smoker	60 (44.4%)
Missing Data	6 (4.5%)
Primary Tumor Site	
Bladder	125 (92.6%)
Upper tract	10 (7.4%)
Pathology	
Urothelial	123 (91.1%)
Squamous	7 (5.2%)
Other	5 (3.7%)
ECOG[a]	
0-1	112 (83.0%)
≥ 2	15 (11.1%)
Missing data	8 (5.9%)
Metastatic sites[#]	
LN[b]	90 (66.7%)
Lung	48 (35.6%)
Bone	43 (31.9%)
Liver	37 (27.4%)
BMI[c]	
< 25	55 (40.7%)
25-30	43 (31.9%)
≥ 30	23 (17.0%)
Missing data	14 (10.4%)
ICI[d] *Line*	
First	51 (37.8%)
Second or later	84 (62.2%)

[a]Eastern Cooperative Oncology Group Performance Status Scale [b]Lymph Nodes [c]Body Mass Index [d]Immune Checkpoint Inhibitors

[#]Numbers do not add up to 100% because many patients had multiple sites of metastases

For peer review only - http://bmjopen.bmj.com/site/about/guidelines.xhtml

Table 2: Univariable and Multivariable analysis for overall survival

OS	Univariable			Multivariable		
	HR	95% CI	p-value	HR	95% CI	p-value
BMI[a] ≥ 30 vs < 30	0.63	0.45-0.88	0.012	0.40	0.17-0.96	0.040
Male vs Female	0.95	0.58-1.57	0.867			
Age ≥ 60 vs < 60	1.88	0.65-5.45	0.235			
ECOG[b] ≥ 2 vs 0-1	1.68	0.86-3.29	0.125	2.21	1.02-4.78	0.042
ICI[c] Line ≥ 2 vs 1	2.39	1.43-3.97	0.001	1.80	1.31-2.48	0.000
NLR[d] ≥ 4 vs < 4	2.46	1.53-3.94	0.000	2.66	1.58-4.49	0.000
Bone metastasis Present vs Absent	2.38	1.52-3.73	0.000	1.98	1.17-3.35	0.010
Lung metastasis Present vs Absent	1.88	1.21-2.93	0.005			
Liver metastasis Present vs Absent	1.92	1.2-3.09	0.006			

[a]Body mass index [b]Eastern cooperative oncology group performance status scale [c]Immune checkpoint inhibitor [d]Neutrophil-to-lymphocyte ratio

Figure 1 Overall response rate (A) and overall survival (B) according to body mass index BMI

Figure 2 Overall response rate according to metastatic sites (A) Bone (B) Liver (C) Lymph nodes (D) Lung

Figure 3 Overall survival according to metastatic sites (A) Bone (B) Liver (C) Lung (D) Lymph nodes

Supplemental Figure 1 Overall response rate according to neutrophil to lymphocyte ratio NLR

Supplemental Figure 2 Overall survival according to NLR

Supplemental Figure 3 Overall response rate and overall survival according to BMI (A), bone metastases (B) and NLR (C) in the cohort of patients treated with immune checkpoint inhibitors alone

For peer review only

For peer review only - http://bmjopen.bmj.com/site/about/guidelines.xhtml

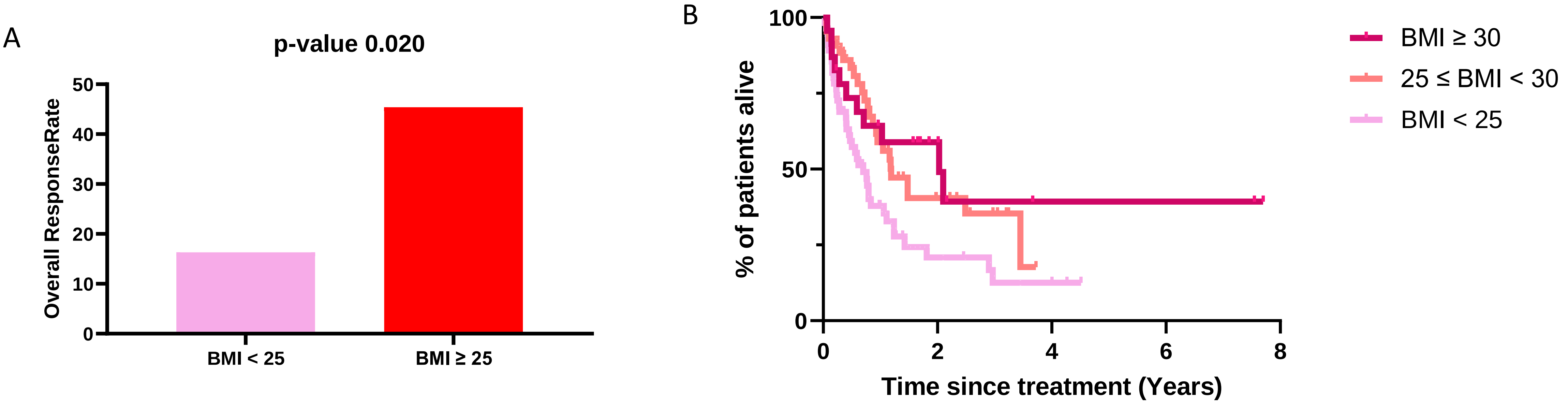

Number at risk

BMI ≥ 30	23	6	2	2	0
25 ≤ BMI < 30	43	9	0	0	0
BMI < 25	55	5	0	0	0

Figure 1. Overall response rate and overall survival according to body mass index (BMI) groups

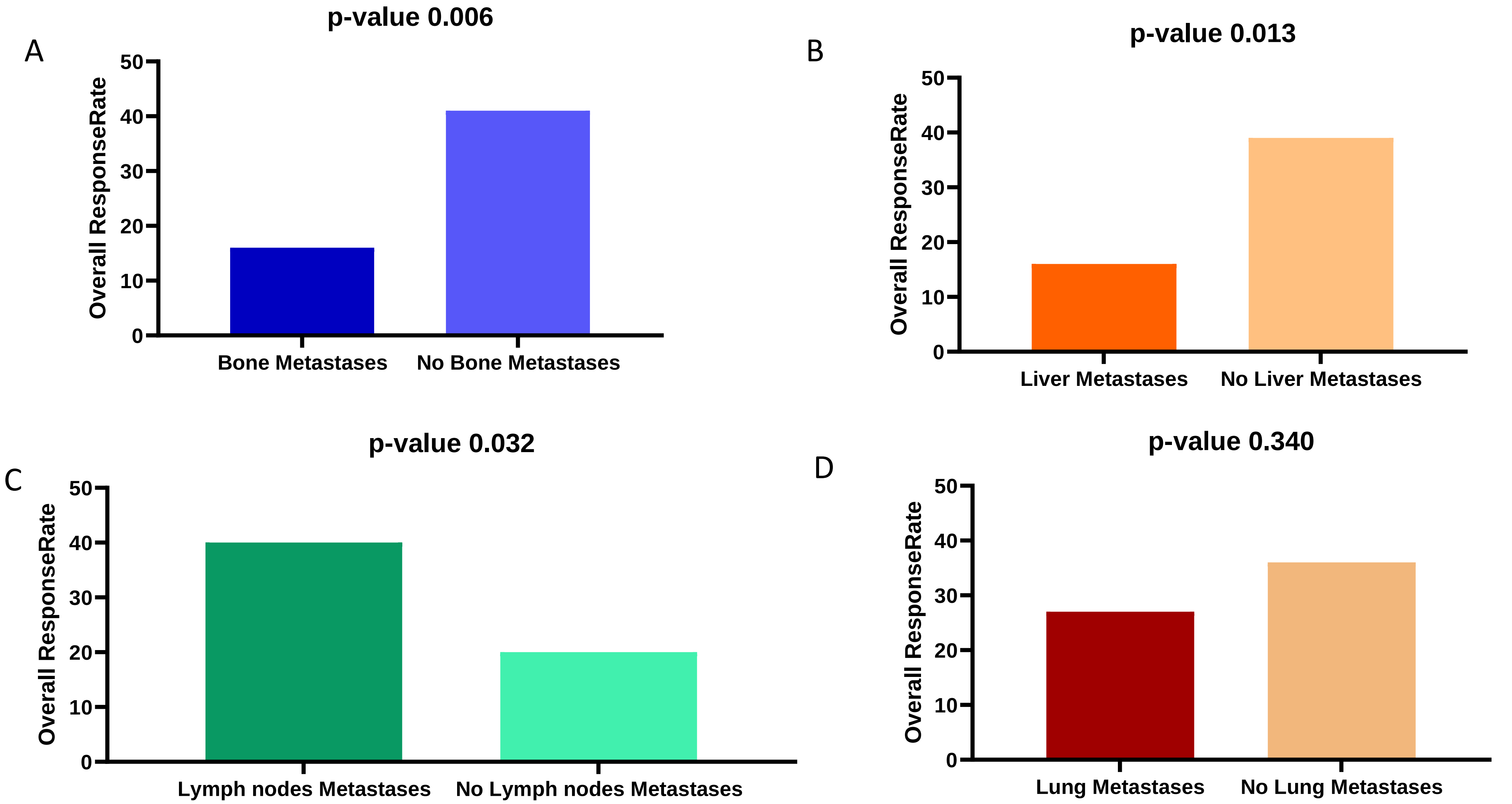

Figure 2. Overall response rate according to metastatic sites

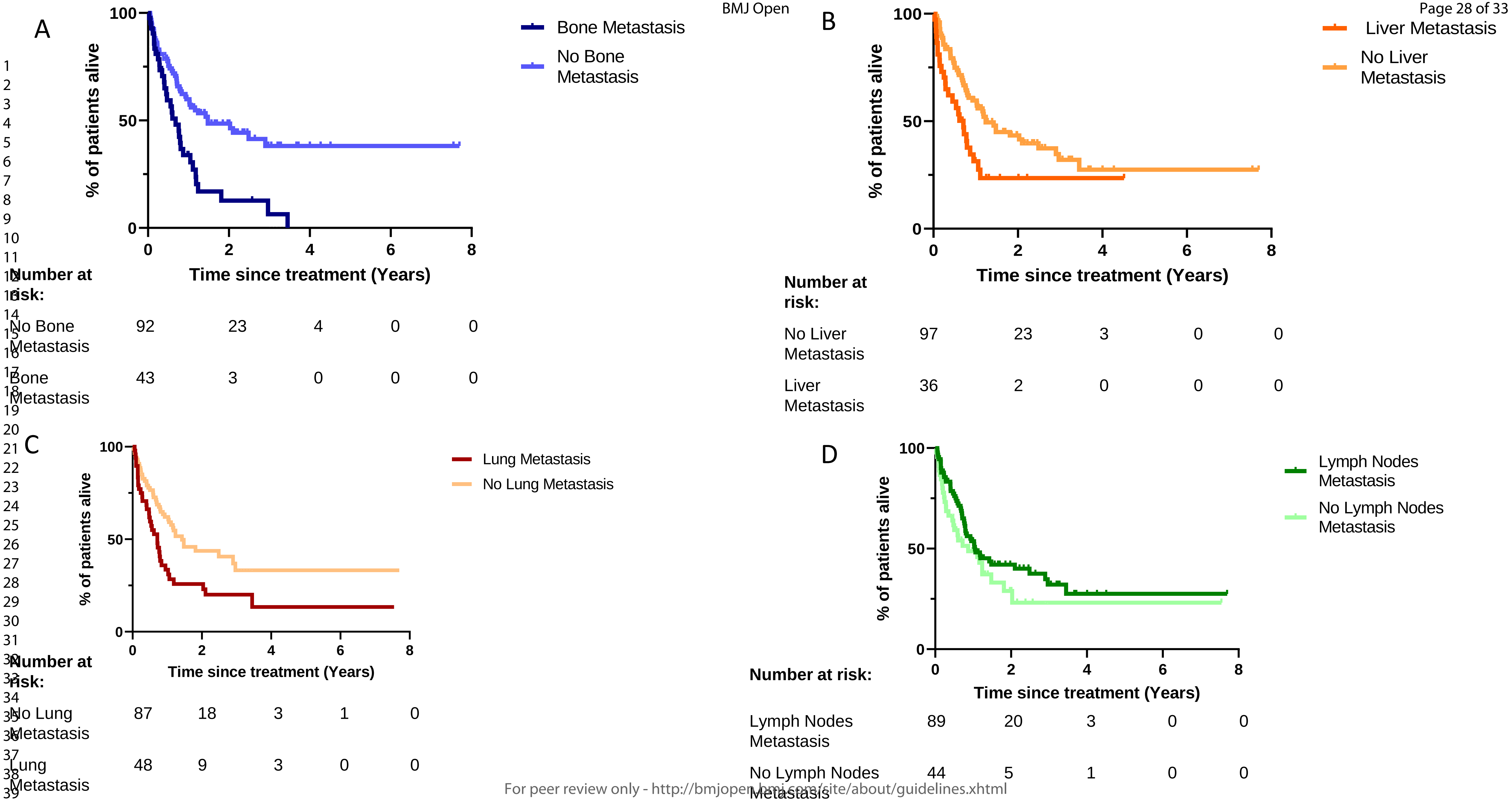

For peer review only - http://bmjopen.bmj.com/site/about/guidelines.xhtml

Figure 3 Overall survival according to metastatic sites

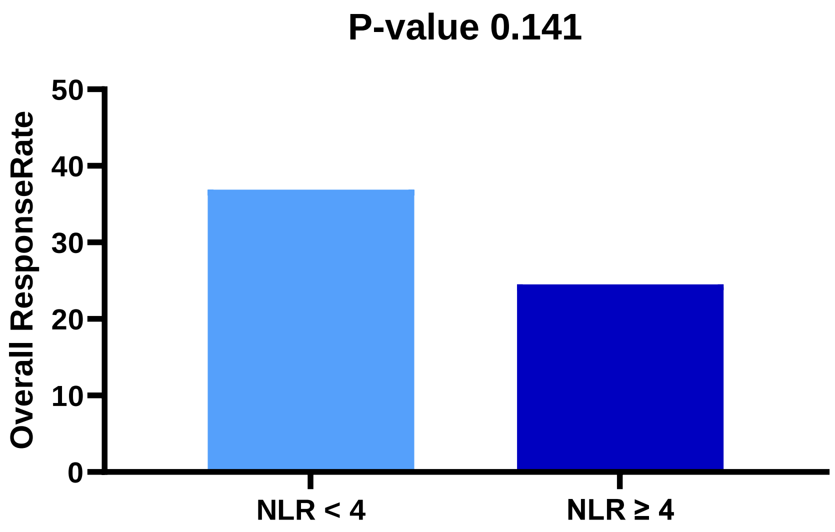

Supplemental Figure 1 Overall response rate according to neutrophil-to-lymphocytes ratio (NLR)

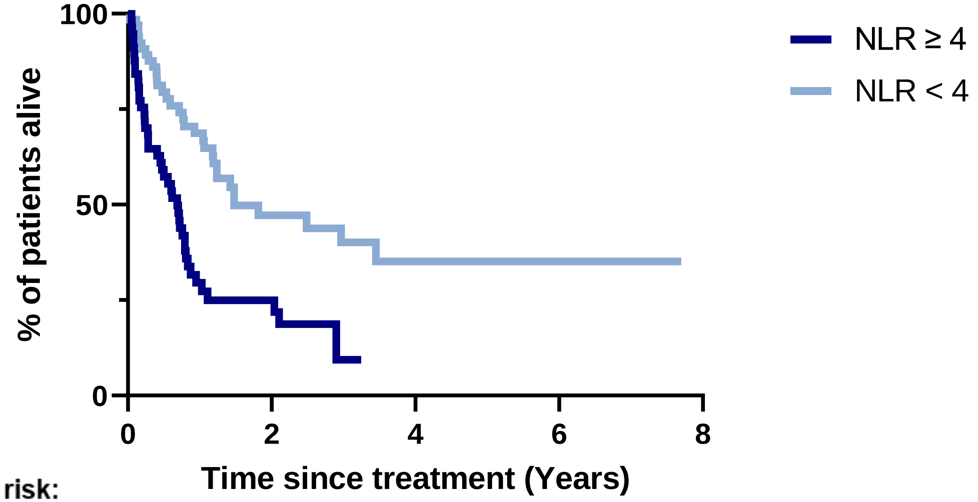

Number at risk:

NLR < 4	65	16	5	2	0
NLR ≥ 4	57	8	0	0	0

Supplemental Figure 2 Overall survival according to NLR

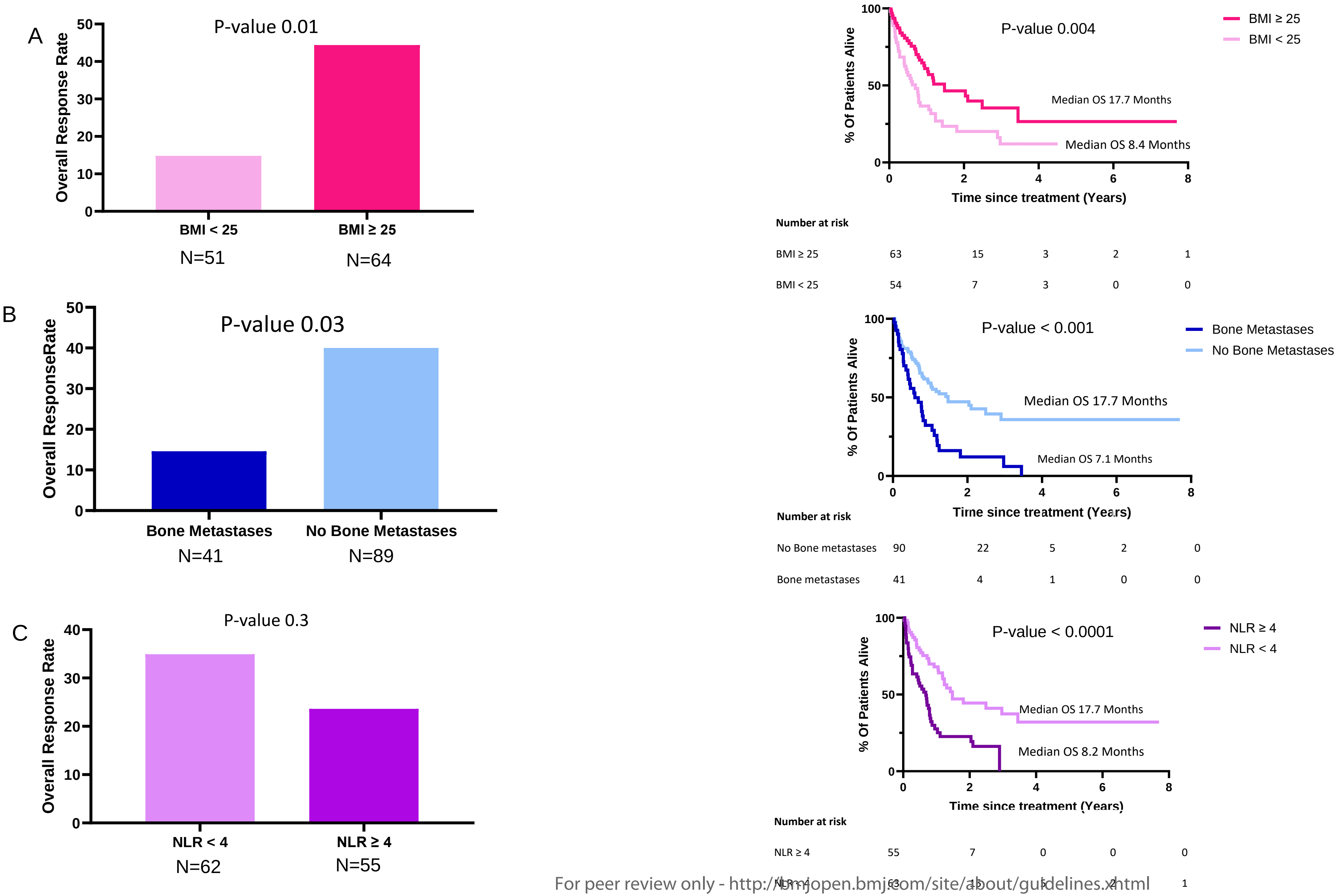

For peer review only - http://bmjopen.bmj.com/site/about/guidelines.xhtml

Supplemental Figure 3 ORR and OS according to BMI (A) Bone metastases (B) and NLR (C) in the cohort of patients treated with IO alone

	$BMI^a \geq 25$	$BMI^a < 25$	Lymphnode	Lung	Bone	Liver
ORR^b (CR^c+PR^d), %	45.4	16.3	40	27	16	16
CR, n (%)	8 (12.1)	1 (1.8)	7 (7.77%)	2 (4.16%)	1 (2.1%)	1 (2.5%)
PR, n (%)	22 (33.3)	8 (14.5)	29 (32.2%)	11 (22.9%)	6 (13.9%)	5 (13.5%)
SD^e, n (%)	7 (10.6)	10 (18.1)	10 (11.1%)	3 (6.25%)	6 (13.9%)	3 (8.1%)
PD^f, n (%)	24 (43.9)	36 (65.4)	44 (48.8%)	32 (66.6%)	30 (69.7%)	28 (75.6%)

[a]Body mass index [b]Overall response rate [c]Complete response [d]Partial response [e]Stable disease [f]Progressive disease

Supplemental Table: Responses according to clinical variables [a]BMI and metastatic sites

For peer review only

STROBE Statement—Checklist of items that should be included in reports of ***cohort studies***

	Item No	Recommendation
**Title and abstract**	1 ✓	(*a*) Indicate the study's design with a commonly used term in the title or the abstract
		(*b*) Provide in the abstract an informative and balanced summary of what was done and what was found
**Introduction**		Abstract - Page 2
Background/rationale ✓	2	Explain the scientific background and rationale for the investigation being reported
Objectives ✓	3	State specific objectives, including any prespecified hypotheses
**Methods**		Page 3-4
Study design ✓	4	Present key elements of study design early in the paper Page 5
Setting ✓	5	Describe the setting, locations, and relevant dates, including periods of recruitment, exposure, follow-up, and data collection Page 5
Participants ✓	6	(*a*) Give the eligibility criteria, and the sources and methods of selection of participants. Describe methods of follow-up Page 5
		(*b*) For matched studies, give matching criteria and number of exposed and unexposed N/A
Variables ✓	7	Clearly define all outcomes, exposures, predictors, potential confounders, and effect modifiers. Give diagnostic criteria, if applicable Page 5
Data sources/ measurement ✓	8*	For each variable of interest, give sources of data and details of methods of assessment (measurement). Describe comparability of assessment methods if there is more than one group Page 5
Bias ✓	9	Describe any efforts to address potential sources of bias Page 13
Study size ✓	10	Explain how the study size was arrived at Page 5
Quantitative variables ✓	11	Explain how quantitative variables were handled in the analyses. If applicable, describe which groupings were chosen and why Page 5
Statistical methods ✓	12	(*a*) Describe all statistical methods, including those used to control for confounding
		(*b*) Describe any methods used to examine subgroups and interactions Page 5 & 6
		(*c*) Explain how missing data were addressed
		(*d*) If applicable, explain how loss to follow-up was addressed
		(*e*) Describe any sensitivity analyses
**Results**		
Participants ✓	13*	(a) Report numbers of individuals at each stage of study—eg numbers potentially eligible, examined for eligibility, confirmed eligible, included in the study, completing follow-up, and analysed Page 5
		(b) Give reasons for non-participation at each stage
		(c) Consider use of a flow diagram
Descriptive data ✓	14*	(a) Give characteristics of study participants (eg demographic, clinical, social) and information on exposures and potential confounders Page 6
		(b) Indicate number of participants with missing data for each variable of interest Page 18
		(c) Summarise follow-up time (eg, average and total amount) Page 6
Outcome data ✓	15*	Report numbers of outcome events or summary measures over time Page 6-7
Main results ✓	16	(*a*) Give unadjusted estimates and, if applicable, confounder-adjusted estimates and their precision (eg, 95% confidence interval). Make clear which confounders were adjusted for and why they were included Page 19
		(*b*) Report category boundaries when continuous variables were categorized Page 19
		(*c*) If relevant, consider translating estimates of relative risk into absolute risk for a meaningful time period N/A

For peer review only

Other analyses	✓	17	Report other analyses done—eg analyses of subgroups and interactions, and sensitivity analyses	Page 6-7
**Discussion**				
Key results	✓	18	Summarise key results with reference to study objectives	Page 8-12
Limitations	✓	19	Discuss limitations of the study, taking into account sources of potential bias or imprecision. Discuss both direction and magnitude of any potential bias	Page 13
Interpretation	✓	20	Give a cautious overall interpretation of results considering objectives, limitations, multiplicity of analyses, results from similar studies, and other relevant evidence	Page 8-13
Generalisability	✓	21	Discuss the generalisability (external validity) of the study results	Page 13
**Other information**				
Funding	✓	22	Give the source of funding and the role of the funders for the present study and, if applicable, for the original study on which the present article is based	Page 18

*Give information separately for exposed and unexposed groups.

Note: An Explanation and Elaboration article discusses each checklist item and gives methodological background and published examples of transparent reporting. The STROBE checklist is best used in conjunction with this article (freely available on the Web sites of PLoS Medicine at http://www.plosmedicine.org/, Annals of Internal Medicine at http://www.annals.org/, and Epidemiology at http://www.epidem.com/). Information on the STROBE Initiative is available at http://www.strobe-statement.org.

For peer review only
